# The success story of *Labiobaetis* Novikova & Kluge in the Philippines (Ephemeroptera, Baetidae), with description of 18 new species

**DOI:** 10.3897/zookeys.1002.58017

**Published:** 2020-12-10

**Authors:** Thomas Kaltenbach, Jhoana M. Garces, Jean-Luc Gattolliat

**Affiliations:** 1 Museum of Zoology, Palais de Rumine, Place Riponne 6, CH-1005 Lausanne, Switzerland Museum of Zoology Lausanne Switzerland; 2 University of Lausanne (UNIL), Department of Ecology and Evolution, CH-1015 Lausanne, Switzerland University of Lausanne (UNIL), Department of Ecology and Evolution Lausanne Switzerland; 3 Department of Biology, School of Science and Engineering, Ateneo de Manila University, Quezon City, 1108 Metro Manila, Philippines Ateneo de Manila University Quezon City Philippines

**Keywords:** COI, genetic distance, integrated taxonomy, Molecular Operational Taxonomic Unit (MOTU), Southeast Asia

## Abstract

Material collected between 1994 and 2020 in the Philippines, covering most main islands like Luzon, Mindoro, Palawan, Negros, Cebu, Leyte, and Mindanao and some smaller islands, substantially increased our knowledge of *Labiobaetis* Novikova & Kluge in this archipelago. Only three species were previously reported: *L. molawinensis* (Müller-Liebenau, 1982) and *L. sumigarensis* (Müller-Liebenau, 1982) from larvae and *L. boettgeri* (Ulmer, 1924) from adults. Eighteen new species have been identified using a combination of morphology and genetic distance (COI, Kimura 2-parameter). They are described and illustrated based on their larvae and a key to all species in the Philippines is provided. The total number of *Labiobaetis* in the Philippines has increased to 21 species. Additional diversity of *Labiobaetis* based on molecular evidence only is presented as Molecular Operational Taxonomic Units (MOTUs) without description. The interspecific K2P distances in the Philippines are between 15% and 27%, the intraspecific distances are usually between 0% and 3%. The total number of *Labiobaetis* species worldwide is augmented to 144.

## Introduction

The family Baetidae has the highest species diversity among mayflies, comprising 1,070 species in 110 genera (Sartori and Brittain 2015; Jacobus et al. 2019), which is approx. one quarter of all mayfly species worldwide (Gattolliat and Nieto 2009; Jacobus et al. 2019). They have a cosmopolitan distribution except Antarctica and New Zealand. Investigations of the molecular phylogeny of the Order Ephemeroptera revealed the relatively primitive status of the family (Ogden and Whiting 2005; Ogden et al. 2009).

The genus *Labiobaetis* Novikova & Kluge (Novikova and Kluge 1987) is one of the richest genera of mayflies with 126 previously described species (Barber-James et al. 2013; Webb 2013; Kubendran et al. 2014, 2015; Shi and Tong 2014; Gattolliat et al. 2018; Kaltenbach and Gattolliat 2018, 2019, 2020). The distribution of *Labiobaetis* is nearly worldwide, except for the Neotropical realm, New Zealand and New Caledonia. The status and validity of the genus has often been a subject of controversy for a long time, but nowadays *Labiobaetis* is widely accepted as a valid genus (Gattolliat 2001; Fujitani et al. 2003; Fujitani 2008; McCafferty et al. 2010; Gattolliat and Staniczek 2011; Kluge and Novikova 2011, 2014, 2016; Kluge 2012; Webb 2013; Kubendran et al. 2014, 2015; Shi and Tong 2014). The history and concept of the genus *Labiobaetis* were recently summarized in detail (Shi and Tong 2014; Kaltenbach and Gattolliat 2018). Recently, Kluge and Novikova (2016) established a new tribe Labiobaetini including the genera *Labiobaetis* and *Pseudopannota* Waltz & McCafferty, 1987, based on a unique combination of imaginal and larval characters. All Oriental species previously transferred to *Pseudocloeon* (Lugo-Ortiz et al. 1999) were formerly reassigned to *Labiobaetis* by Shi and Tong (2014); the concept of *Pseudocloeon* is therefore limited to the type species *P. kraepelini* Klapálek, 1905 from Java. Molecular reconstructions indicated that the concept of *Labiobaetis* is probably at least diphyletic (Monaghan et al. 2005; Gattolliat et al. 2008).

Recently, integrative taxonomy was done on collections from the diverse and poorly explored Southeast Asia and New Guinea regions where 47 species were described and named (Kaltenbach and Gattolliat 2018, 2019, 2020). This contribution will focus on the Philippines archipelago, one of the highly diverse parts of the Oriental region.

The megadiversity of the Philippines is partly attributed to the complex biogeographic history and isolation of the archipelago. The discussion of the biogeographic history of the Philippine archipelago includes landmass movements, collisions between landmasses of different origin in Miocene, and temporary Pleistocene land bridges which were possible colonization pathways of species. Originally part of the Eurasian continent, the oldest landmasses of the current Philippines are parts of Palawan, Mindoro, Romblon and Panay. Whether these landmasses were entirely submerged during the drift is still a matter of debate (Vane-Wright 1990; Turner et al. 2001; Hall 2002; Zamoras and Matsuoka 2004; Siler et al. 2012; Heaney et al. 2013), but undoubtedly these current islands are of different origin and age compared to the rest of the country which is of oceanic origin. Hence, it is expected that the adjacent areas (continental Southeast Asia, Taiwan, Borneo and Sulawesi) differ from most of the archipelago. Moreover, when the sea level was low during the Pleistocene, land bridges were formed interconnecting groups of Philippine islands, and the Sundaic landmasses with the western Philippine island (Greater Palawan) (Ong et al. 2002; Welton et al. 2014; Zettel and Freitag 2014; Freitag et al. 2016). This was the major basis of the intra-Philippine biogeographic region (Ong et al. 2002) and is subsequently reflected up to a certain extent on current species distribution (Siler et al. 2012; Zettel and Freitag 2014; Freitag et al. 2016), but not always (Heads 2013 and references therein). In his review of the biogeography of the Philippines, Heads (2013) outlined different species affinities and sister-group relationships of various taxa based on published records and available data – Philippine clades recovered sister to: Madagascar-Asia-Central America, Africa-Asia, Madagascar-Mascarenes-Asia, Indochina/China, Sundaland, Borneo, Sulawesi and further east. Several Philippine-endemic clades indeed have diverse sister groups with widespread intercontinental distributions, as opposed to simply coming from Borneo, Sulawesi or Taiwan as once previously thought. In addition, recent data suggest that even biogeographic regions previously categorized as one single unit (e.g., Greater Luzon) are in fact composed of distinct centres of endemism that correlate with tectonic features (Vallejo 2014), further exemplifying high endemism and niche specialization of species found in the country.

The diversity of *Labiobaetis* in the Philippines was poorly known, as only two species were previously reported from larvae (*L. molawinensis* and *L. sumigarensis* by Müller-Liebenau 1982) and one species from adults only (*L. boettgeri*). Here, we increase the total number of *Labiobaetis* species in the Philippines to 21, based on material collected between 1994 and 2020 on several islands (Figs 48, 49). We describe 18 new species of *Labiobaetis* based on larval stage only. The characters of some of the species groups are complemented based on the results of this study. Additionally, we have new reports of *L. molawinensis* and *L. sumigarensis*. We are also presenting cryptic diversity as Molecular Operational Taxonomic Units (MOTUs) based on molecular evidence only (COI), without description of species (Floyd et al. 2002; Blaxter et al. 2005; Morard et al. 2016).

## Materials and methods

All specimens were collected between 1994 and 2020 by Dr. Hendrik Freitag and his team (Ateneo de Manila University) and preserved in 70%–96% ethanol.

The dissection of larvae was done in Cellosolve (2-Ethoxyethanol) with subsequent mounting on slides with Euparal liquid, using an Olympus SZX7 stereomicroscope.

The DNA of part of the specimens was extracted using non-destructive methods allowing subsequent morphological analysis (see Vuataz et al. 2011 for details). We amplified a 658 bp fragment of the mitochondrial gene cytochrome oxidase subunit 1 (COI) using the primers LCO 1490 and HCO 2198 (Folmer et al. 1994; see Kaltenbach and Gattolliat 2020 for details). Sequencing was done with Sanger’s method (Sanger et al. 1977). The genetic variability between specimens was estimated using Kimura-2-parameter distances (K2P, Kimura 1980), calculated with the program MEGA 7 (Kumar et al. 2016, http://www.megasoftware.net).

The GenBank accession numbers are given in Table 1, nomenclature of gene sequences follows Chakrabarty et al. (2013).

**Table 1.** Sequenced specimens.

Species	Species group	Locality	Specimens catalog #	GenBank #	GenSeq
				(COI)	Nomenclature
*L. dalisay* sp. nov.	*dendrisetis* gr.	Philippines: Luzon	GBIFCH 00763649	MT830940	genseq-2 COI
*L. acei* sp. nov.	*numeratus* gr.	Philippines: Luzon	GBIFCH 00763643	MT830941	genseq-1 COI
			GBIFCH 00763645	MT830942	genseq-2 COI
			GBIFCH 00763651	MT830943	genseq-2 COI
*L. aldabae* sp. nov.	*numeratus* gr.	Philippines: Luzon	GBIFCH 00654913	MT830944	genseq-1 COI
			GBIFCH 00654908	MT830945	genseq-2 COI
			GBIFCH 00763646	MT830946	genseq-2 COI
			GBIFCH 00763648	MT830947	genseq-2 COI
		Philippines: Negros	GBIFCH 00654889	MT830948	genseq-2 COI
*L. camiguinensis* sp. nov.	*numeratus* gr.	Philippines: Camiguin	GBIFCH 00654915	MT830949	genseq-1 COI
*L. lachicae* sp. nov.	*numeratus* gr.	Philippines: Mindanao	GBIFCH 00654891	MT830950	genseq-1 COI
*L. palawano* sp. nov.	*numeratus* gr.	Philippines: Busuanga	GBIFCH 00763688	MT830987	genseq-1 COI
		Philippines: Palawan	GBIFCH 00763679	MT830988	genseq-2 COI
*L. sabordoi* sp. nov.	*numeratus* gr.	Philippines: Negros	GBIFCH 00654878	MT830951	genseq-2 COI
		Philippines: Romblon	GBIFCH 00763674	MT830952	genseq-2 COI
*L. gamay* sp. nov.	*operosus* gr.	Philippines: Mindoro	GBIFCH 00654922	MT830953	genseq-2 COI
			GBIFCH 00763637	MT830954	genseq-2 COI
			GBIFCH 00763639	MT830955	genseq-2 COI
		Philippines: Luzon	GBIFCH 00763655	MT830956	genseq-2 COI
			GBIFCH 00763657	MT830957	genseq-2 COI
			GBIFCH 00763658	MT830958	genseq-2 COI
*L. pangantihoni* sp. nov.	*operosus* gr.	Philippines: Palawan	GBIFCH 00763684	MT830959	genseq-2 COI
*L. tagbanwa* sp. nov.	*operosus* gr.	Philippines: Palawan	GBIFCH 00654885	MT830960	genseq-2 COI
			GBIFCH 00763681	MT830961	genseq-2 COI
			GBIFCH 00763680	MT830962	genseq-2 COI
*L. valdezorum* sp. nov.	*operosus* gr.	Philippines: Negros	GBIFCH 00654888	MT830963	genseq-1 COI
			GBIFCH 00654882	MT830964	genseq-2 COI
			GBIFCH 00654879	MT830965	genseq-2 COI
			GBIFCH 00654880	MT830966	genseq-2 COI
		Philippines: Cebu	GBIFCH 00763671	MT830967	genseq-2 COI
*L. wantzeni* sp. nov.	*operosus* gr.	Philippines: Camiguin	GBIFCH 00654898	MT830968	genseq-1 COI
			GBIFCH 00654897	MT830969	genseq-2 COI
			GBIFCH 00763641	MT830970	genseq-2 COI
			GBIFCH 00763642	MT830971	genseq-2 COI
			GBIFCH 00654896	MT830972	genseq-2 COI
			GBIFCH 00654900	MT830973	genseq-2 COI
*L. baganii* sp. nov.	*sumigarensis* gr.	Philippines: Mindanao	GBIFCH 00654895	MT830974	genseq-1 COI
		Philippines: Camiguin	GBIFCH 00654899	MT830975	genseq-2 COI
*L. delocadoi* sp. nov.	*sumigarensis* gr.	Philippines: Cebu	GBIFCH 00654886	MT830976	genseq-1 COI
		Philippines: Leyte	GBIFCH 00763668	MT830977	genseq-2 COI
*L. freitagi* sp. nov.	*sumigarensis* gr.	Philippines: Palawan	GBIFCH 00763677	MT830978	genseq-2 COI
			GBIFCH 00763678	MT830979	genseq-2 COI
			GBIFCH 00763682	MT830980	genseq-2 COI
			GBIFCH 00763683	MT830981	genseq-2 COI
*L. pelingeni* sp. nov.	*sumigarensis* gr.	Philippines: Negros	GBIFCH 00654901	MT830982	genseq-2 COI
		Philippines: Cebu	GBIFCH 00763672	MT830983	genseq-2 COI
*L. giselae* sp. nov.	*vallus* gr.	Philippines: Luzon	GBIFCH 00654911	MT830984	genseq-2 COI
*L. mendozai* sp. nov.	*vallus* gr.	Philippines: Mindanao	GBIFCH 00654894	MT830985	genseq-2 COI

The nomenclature used for Molecular Operational Taxonomic Units (MOTUs) is somewhat different as the one proposed by Morard et al. (2016).

**Figure 1.**
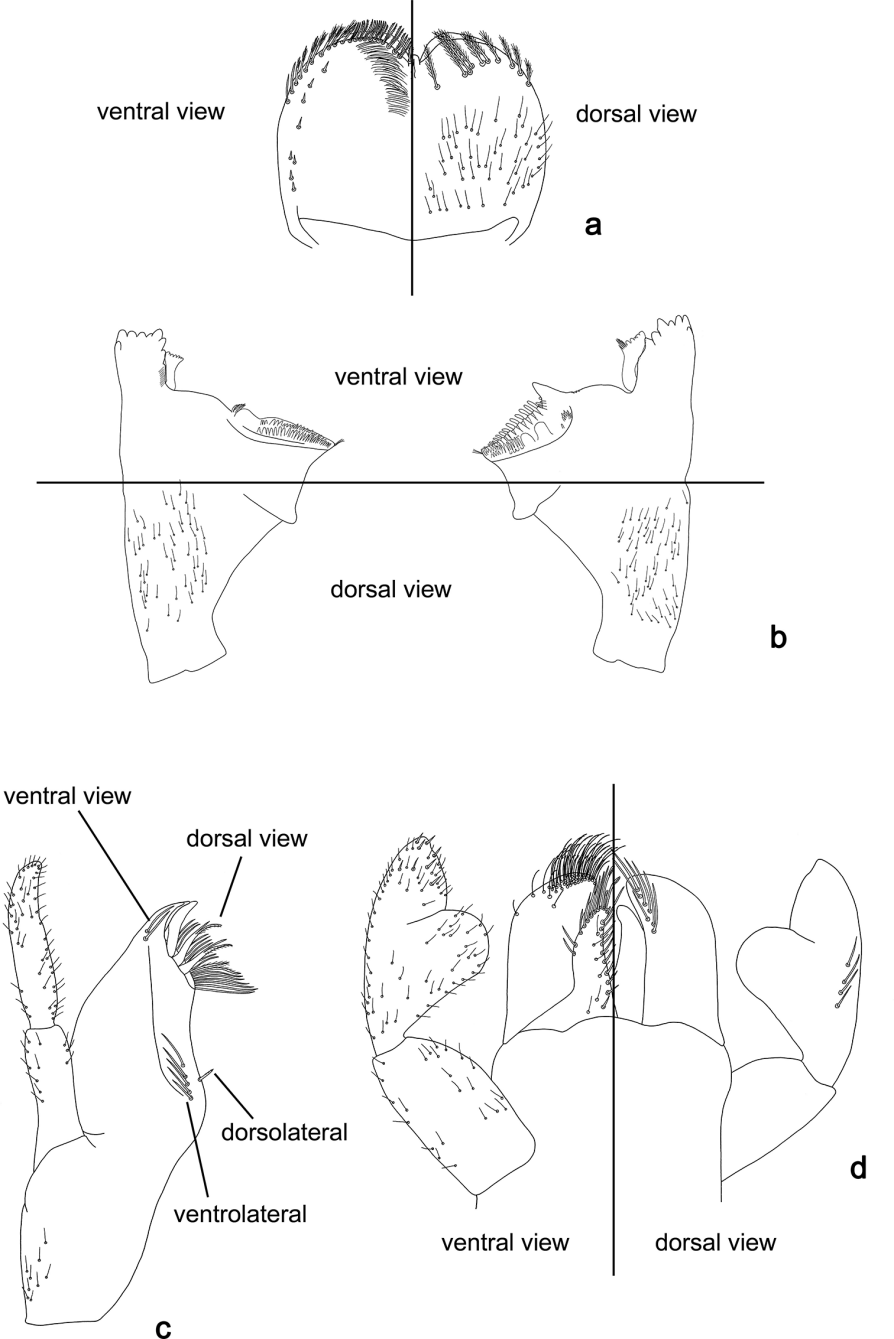
Explanation of drawings **a** labrum **b** mandibles **c** maxilla **d** labium.

Drawings were made using an Olympus BX43 microscope. To facilitate the determination of species and the comparison of important structures, we partly used a combination of dorsal and ventral aspects in one drawing. Explanations are given in Fig. 1.

Photographs of larvae were taken using a Canon EOS 6D camera and the Visionary Digital Passport imaging system (http://www.duninc.com) and processed with Adobe Photoshop Lightroom (http://www.adobe.com) and Helicon Focus version 5.3 (http://www.heliconsoft.com). Photographs were subsequently enhanced with Adobe Photoshop Elements 13.

The distribution maps were generated with SimpleMappr (https://simplemappr.net, Shorthouse 2010). Google Earth (http://www.google.com/earth/download/ge/) was used to attribute approximate GPS coordinates to sample locations of Müller-Liebenau (1982).

The taxonomic descriptions were generated with a DELTA (Dallwitz 1980; Dallwitz et al. 1999; Coleman et al. 2010) database containing the morphological states of characters of the *Labiobaetis* species of the Philippines.

The dichotomous key was elaborated with the support of DKey version 1.3.0 (http://drawwing.org/dkey, Tofilski 2018).

**Figure 2.**
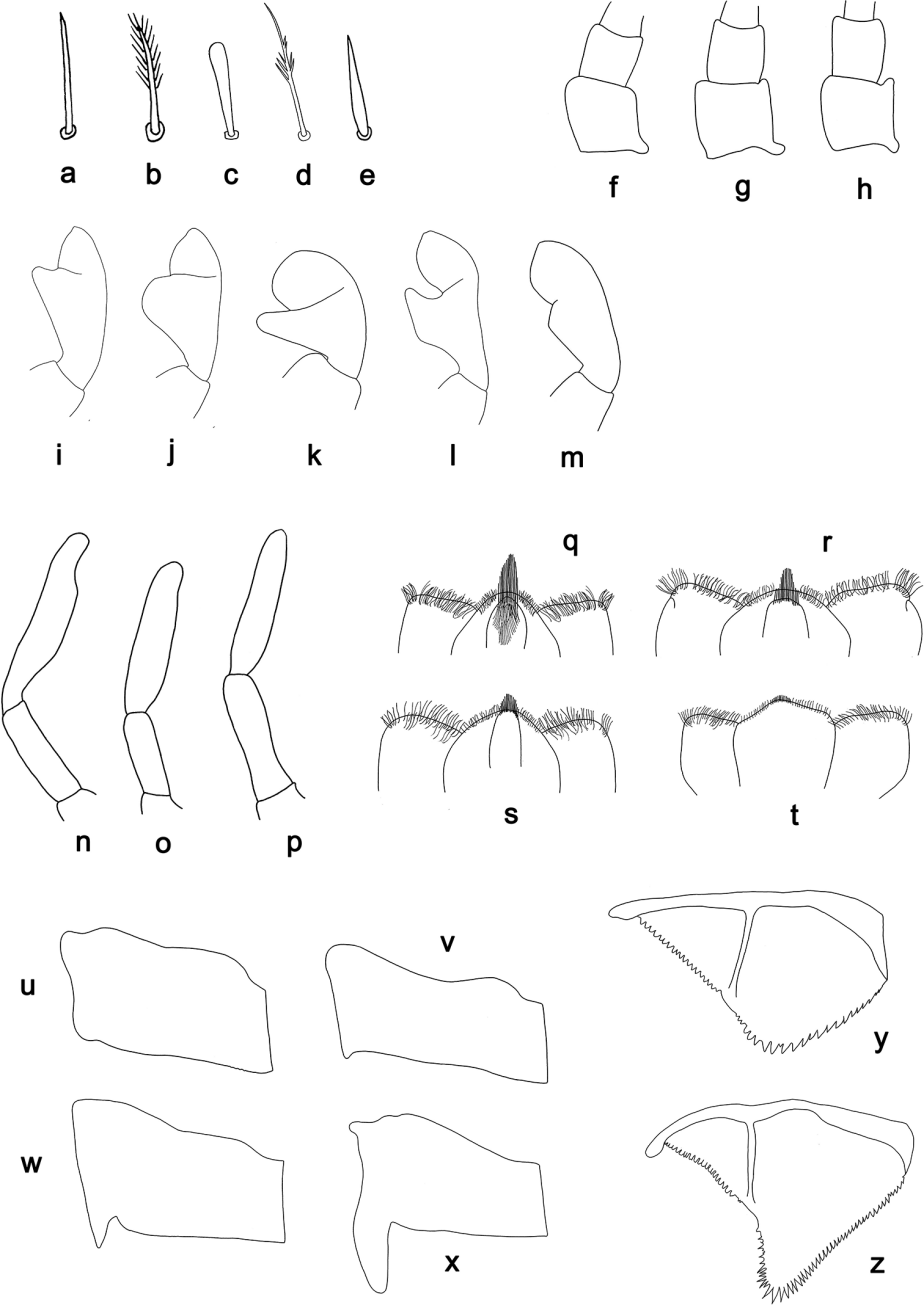
*Labiobaetis*, character states of selected characters **a–e** setae of the submarginal arc on the dorsal surface of the labrum: **a** simple **b** feathered **c** clavate **d** dendritic **e** lanceolate **f–h** distolateral process at scape of antenna: **f** absent **g** poorly developed **h** well developed **i–m** labial palp, distomedial protuberance of segment II: **i** thumb-like **j** broad thumb-like **k** slender thumb-like **l** hook-like **m** small thumb-like **n–p** distolateral excavation at maxillary palp segment II: **n** well developed **o** poorly developed **p** absent **q–t** hypopharynx, medial tuft of stout setae: **q** well developed, long **r** well developed, average length **s** well developed, short **t** poorly developed **u–x** hind protoptera: **u** absent **v** minute **w** small **x** well developed **y, z** paraproct: **y** distally not expanded **z** distally expanded.

The terminology follows Hubbard (1995) and Kluge (2004). The character states of some of the characters are depicted in Fig. 2.

Abbreviations:

**AdMU** Ateneo de Manila University, Quezon City (Philippines)

**MZL** Musée de Zoologie Lausanne (Switzerland)

**PCSD** Palawan Council for Sustainable Development, Puerto Princesa, Palawan (Philippines)

**PNM**Museum of Natural History of the Philippine National Museum, Manila (Philippines)

**ZSM**Zoologische Staatssammlung München (Germany).

## Results

### List of *Labiobaetis* species from the Philippines

*dendrisetis* group

1. *L. dalisay* sp. nov.

*numeratus* group

2. *L. acei* sp. nov.

3. *L. aldabae* sp. nov.

4. *L. camiguinensis* sp. nov.

5. *L. lachicae* sp. nov.

6. *L. palawano* sp. nov.

7. *L. sabordoi* sp. nov.

*operosus* group

8. *L. gamay* sp. nov.

9. *L. pangantihoni* sp. nov.

10. *L. tagbanwa* sp. nov.

11. *L. valdezorum* sp. nov.

12. *L. wantzeni* sp. nov.

*sumigarensis* group

13. *L. molawinensis* (Müller-Liebenau, 1982)

14. *L. sumigarensis* (Müller-Liebenau, 1982)

15. *L. baganii* sp. nov.

16. *L. delocadoi* sp. nov.

17. *L. freitagi* sp. nov.

18. *L. pelingeni* sp. nov.

*vallus* group

19. *L. giselae* sp. nov.

20. *L. mendozai* sp. nov.

Not assigned to a group

21. *L. boettgeri* (Ulmer, 1924), no further treatment in this study.

### *Labiobaetis dendrisetis* group of species (new group of species)

Following combination of characters: A) dorsal surface of labrum with submarginal arc of dendritic setae; B) labial palp segment II slender or small thumb-like; C) labial palp segment III wide; D) maxillary palp shorter than galea-lacinia, rather thick; E) seven pairs of gills.

#### Labiobaetis dalisay sp. nov.

Taxon classificationAnimaliaEphemeropteraBaetidae

AC1E7D76-CA66-56BB-A87D-934B80CBD129

http://zoobank.org/0CF1B9F8-0208-4EF9-BF95-379DBFAA2FF0

**Figure 3.**
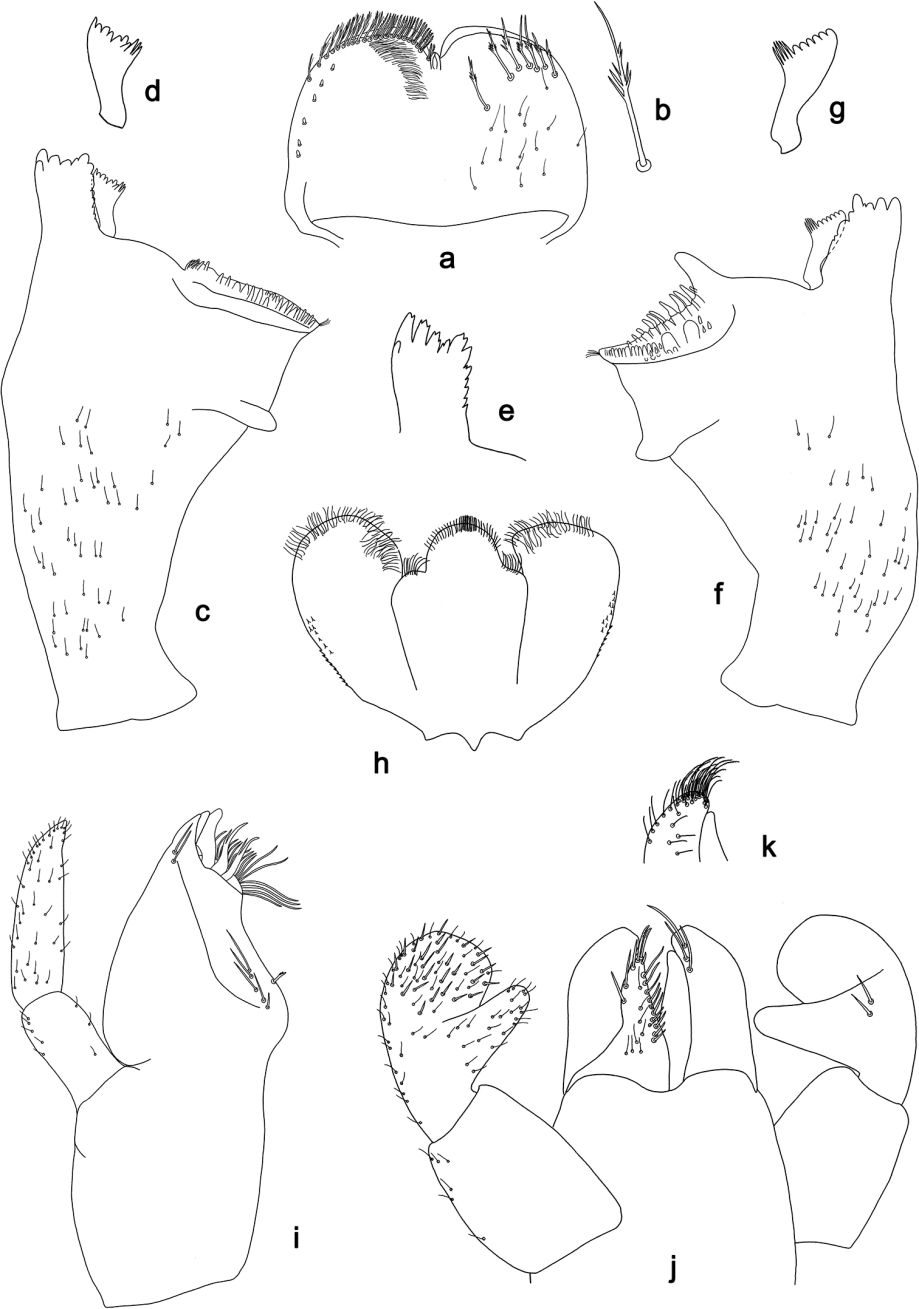
*Labiobaetis dalisay* sp. nov., larva morphology **a** labrum **b** seta of arc on dorsal surface of labrum **c** right mandible **d** right prostheca **e** right incisor and kinetodontium **f** left mandible **g** left prostheca **h** hypopharynx and superlinguae **i** maxilla **j** labium **k** apex of paraglossa.

**Figure 4.**
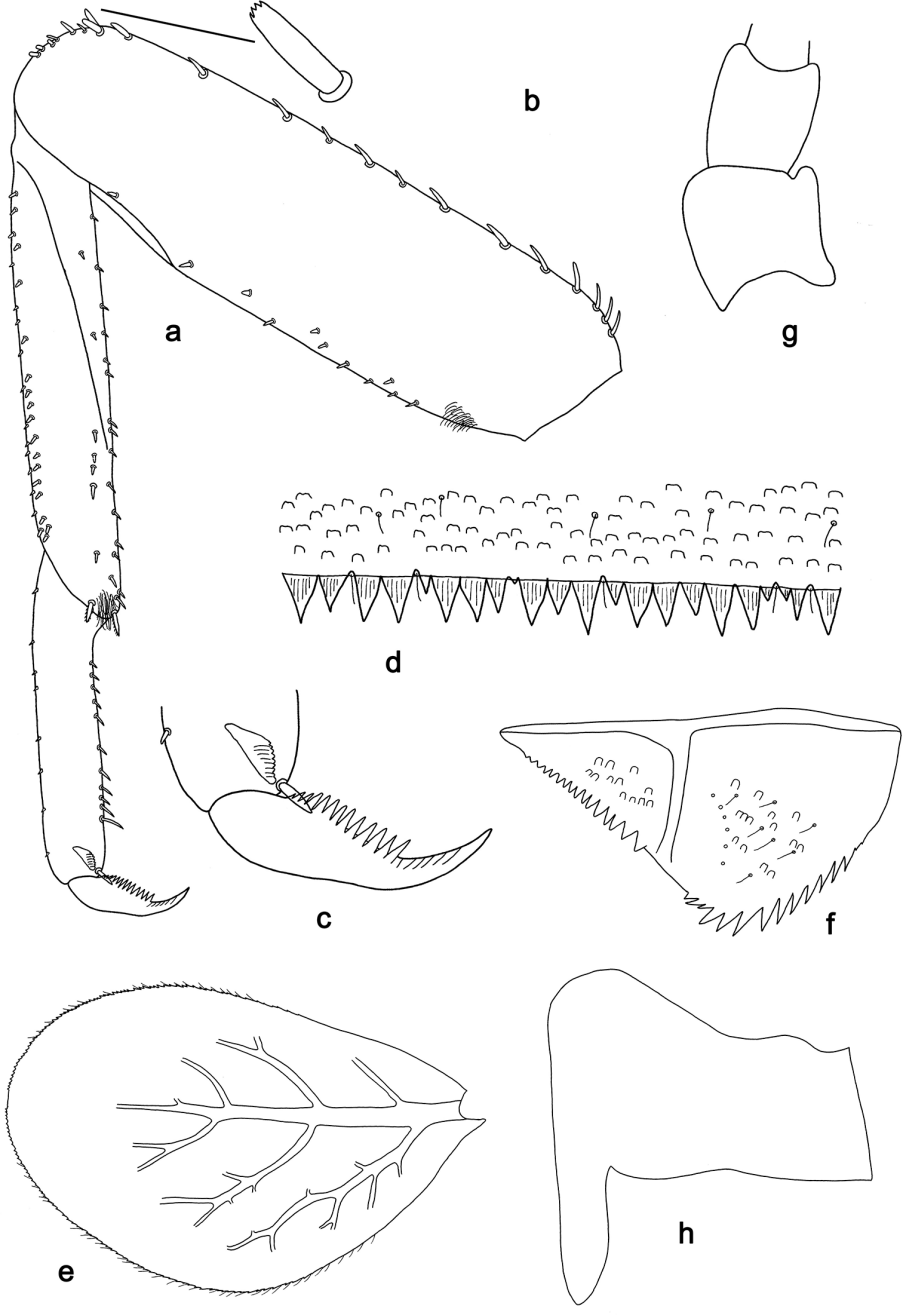
*Labiobaetis dalisay* sp. nov., larva morphology **a** foreleg **b** seta at apex of femur **c** fore claw **d** tergum IV **e** gill IV **f** paraproct **g** antennal scape **h** metanotum.

Figures 3 , 4 , 41a , 48b

##### Diagnosis.

**Larva.** Following combination of characters: A) dorsal surface of labrum with 6–8 long, dendritic setae; B) labial palp segment II with a slender thumb-like distomedial protuberance, segment III subrectangular; C) right mandible without row of thin setae at inner margin of innermost denticle; D) fore femur length 3.4 × maximum width, dorsal margin with 10–19 curved, spine-like setae; E) hind protoptera well developed; F) paraproct distally not expanded, with ca. 15 stout, marginal spines.

##### Description.

**Larva** (Figs 3, 4, 41a). Body length 4.8–6.5 mm. Cerci ca. ½ of body length. Paracercus ca. 2/3 of cerci length. Antenna approx. twice as long as head length.

***Colouration*.** Head, thorax, and abdomen dorsally brown with bright pattern as in Fig. 41a, fore protoptera light brown with darker striation. Head, thorax, and abdomen ventrally light brown. Legs light brown, femur basally and apically brown with a distomedial brown spot and a brown streak distomedially along dorsal margin, tibia basally with brown area, tarsus medially brown. Caudalii light brown with a brown band both at base and at ca. 1/3 of cerci length, cerci distally brown.

***Antenna*** (Fig. 4g) with scape and pedicel subcylindrical, with well-developed distolateral process at scape.

***Labrum*** (Fig. 3a, b). Rectangular, length 0.7 × maximum width. Distal margin with medial emargination and a small process. Dorsally with medium, fine, simple setae scattered over surface; submarginal arc of setae composed of 6–8 long, dendritic setae. Ventrally with marginal row of setae composed of anterolateral long, feathered setae and medial long, bifid, pectinate setae; ventral surface with ca. six short, spine-like setae near lateral and anterolateral margin.

***Right mandible*** (Fig. 3c, d, e). Incisor and kinetodontium fused. Incisor with five denticles; kinetodontium with three denticles, inner margin of innermost denticle without a row of thin setae; denticles of both incisor and kinetodontium of unused mandibles with secondary dentation. Prostheca robust, apically denticulate. Margin between prostheca and mola slightly convex. Tuft of setae at apex of mola present.

***Left mandible*** (Fig. 3f, g). Incisor and kinetodontium fused. Incisor with four denticles; kinetodontium with three denticles. Prostheca robust, apically with small denticles and comb-shaped structure. Margin between prostheca and mola slightly convex. Subtriangular process long and slender, above level of area between prostheca and mola. Denticles of mola apically constricted. Tuft of setae at apex of mola present.

Both mandibles with lateral margins slightly convex. Basal half with fine, simple setae scattered over dorsal surface.

***Hypopharynx and superlinguae*** (Fig. 3h). Lingua approx. as long as superlinguae. Lingua longer than broad; medial tuft of stout setae poorly developed; distal half laterally expanded. Superlinguae distally rounded; lateral margin rounded; fine, long, simple setae along distal margin.

***Maxilla*** (Fig. 3i). Galea-lacinia ventrally with two simple, apical setae under canines. Inner dorsal row of setae with three denti-setae, distal denti-seta tooth-like, middle and proximal denti-setae slender, bifid and pectinate. Medially with one bipectinate, spine-like seta and five medium to long, simple setae. Maxillary palp approx. as long as length of galea-lacinia; 2-segmented; palp segment II 1.6 × length of segment I; setae on maxillary palp fine, simple, scattered over surface of segments I and II; apex of last segment slightly pointed, with slight excavation at inner distolateral margin.

***Labium*** (Fig. 3j, k). Glossa basally broad, narrowing toward apex; shorter than paraglossa; inner margin with ca. ten spine-like setae increasing in length distally; apex with two long, robust, pectinate setae and one short, robust seta; outer margin with three spine-like setae; ventral surface with fine, simple, scattered setae. Paraglossa sub-rectangular, curved inward; apex rounded; with three rows of long, robust, distally pectinate setae in apical area and 4–6 medium, simple setae in anteromedial area; outer, anterolateral margin with some long, spine-like setae; dorsally with a row of three long, spine-like setae near inner margin. Labial palp with segment I 0.9 × length of segments II and III combined. Segment I ventrally with short, fine, simple setae. Segment II with slender, thumb-like distomedial protuberance; distomedial protuberance 0.5 × width of base of segment III; ventral surface with short, fine, simple setae; dorsally with two or three spine-like, simple setae near outer margin. Segment III subrectangular; length 0.9 × width; ventrally covered with short, spine-like, simple setae and short, fine, simple setae.

***Hind protoptera*** (Fig. 4h) well developed.

***Foreleg*** (Fig. 4a–c). Ratio of foreleg segments 1.4:1.0:0.7:0.3. ***Femur***. Length ca. 3 × maximum width. Dorsal margin with a row of 10–19 curved, spine-like setae, apically rounded and sometimes with minute dentation; length of setae 0.13 × maximum width of femur. Apex rounded, with a pair of spine-like setae, apically rounded and sometimes with minute dentation and some short, stout setae. Many stout, lanceolate setae scattered along ventral margin; femoral patch present. ***Tibia*.** Dorsal margin with two rows of short, spine-like setae. Ventral margin with a row of short, curved, spine-like setae, on apex some longer, partly bipectinate, spine-like setae and a tuft of fine, simple setae. Anterior surface scattered with stout, lanceolate setae. Patellotibial suture present on basal 2/3 area. ***Tarsus*.** Dorsal margin with one or two rows of short, stout setae. Ventral margin with a row of curved, spine-like setae. Claw with one row of 13–15 denticles; distally pointed; with five or six stripes; subapical setae absent.

***Terga*** (Fig. 4d). Surface with irregular rows of W-shaped scale bases and scattered fine, simple setae. Posterior margin of tergum IV with triangular spines, longer than wide.

***Gills*** (Fig. 4e). Present on segments I–VII. Margin with small denticles intercalating fine simple setae. Tracheae extending from main trunk to inner and outer margins. Gill I ca. 2/3 length of segment II. Gill IV as long as length of segments V and 2/3 VI combined. Gill VII as long as length of segments VIII and 1/3 IX combined.

***Paraproct*** (Fig. 4f). Distally not expanded, with ca. 15 stout, marginal spines. Surface scattered with U-shaped scale bases and fine, simple setae. Cercotractor with numerous small, marginal spines.

##### Etymology.

Named after the Filipino word *dalisay* meaning pristine, which describes the localities where the species was collected.

##### Distribution.

Philippines: Luzon (Fig. 48b).

##### Biological aspects.

The specimens were collected at altitudes from 60 m to 400 m, mainly in pristine areas.

##### Type material.

***Holotype*.** Philippines • larva; Luzon, Maria Aurora, Wenceslao, Bingwangan River; 15°45'48"N, 121°25'21"E; 60 m; 05.II.1998; leg. Mendoza; on slide; GBIFCH 00592279; PNM. ***Paratypes*.** Philippines • larva; Luzon, Nueva Ecija, Pantabangan, Candaclan River; 15°46'48"N, 121°13'17"E; 240 m; 05.II.1998; leg. Mendoza; on slide; GBIFCH 00654909; ZSM • 6 larvae; Luzon, Benguet, Tuba, Taloy Sur; 16°21'33"N, 120°30'31"E; 400 m; XI. 1997; leg. Mey; 1 on slide; GenBank: MT830940; GBIFCH 00763649; AdMU; 5 in alcohol; GBIFCH 00515405; MZL.

### *Labiobaetis numeratus* group of species (Kaltenbach and Gattoliat 2019)

Following combination of characters: A) dorsal surface of labrum with submarginal arc of simple setae, 1^st^ and 2^nd^ setae after submedian seta close together; B) labial palp segment II with thumb-like distomedial protuberance; C) glossae with robust setae at inner margin; D) paraglossae with setae at anterolateral and lateral outer margin; E) right mandible with a pronounced hump between prostheca and mola, thin setae at base of mola; F) left mandible with convex margin between prostheca and mola; G) maxillary palp segment II much longer vs. segment I, bent; H) superlinguae sclerotized along margins (Fig. 47d); I) six pairs of gills, mostly oblong; J) hind protoptera present, minute; K) distolateral process at scape absent; L) spines at posterior margin of tergum IV always partly merged, mostly rounded and wider than long; M) femur dorsal margin with a partial second row of spine-like setae; N) ventral margin of tibia with a longer, spine-like seta distally at patellotibial suture.

#### Labiobaetis acei sp. nov.

Taxon classificationAnimaliaEphemeropteraBaetidae

7EA631F0-D6A1-5943-B03C-E861A9E73B25

http://zoobank.org/256AE781-83A3-41C7-A109-78AB9EFA3906

**Figure 5.**
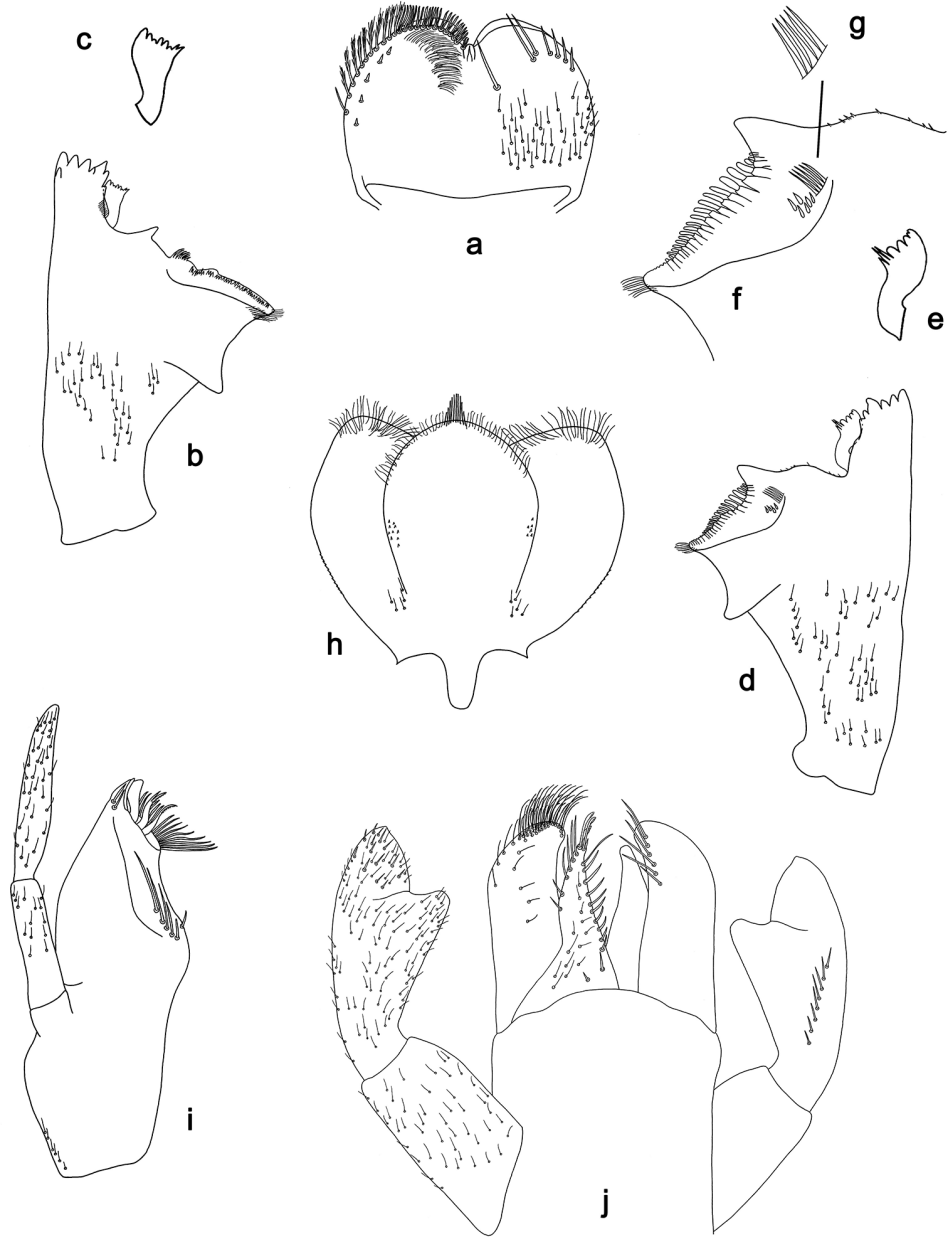
*Labiobaetis acei* sp. nov., larva morphology **a** labrum **b** right mandible **c** right prostheca **d** left mandible **e** left prostheca **f** detail of left mola **g** comb-shaped structure at left mola **h** hypopharynx and superlinguae **i** maxilla **j** labium.

**Figure 6.**
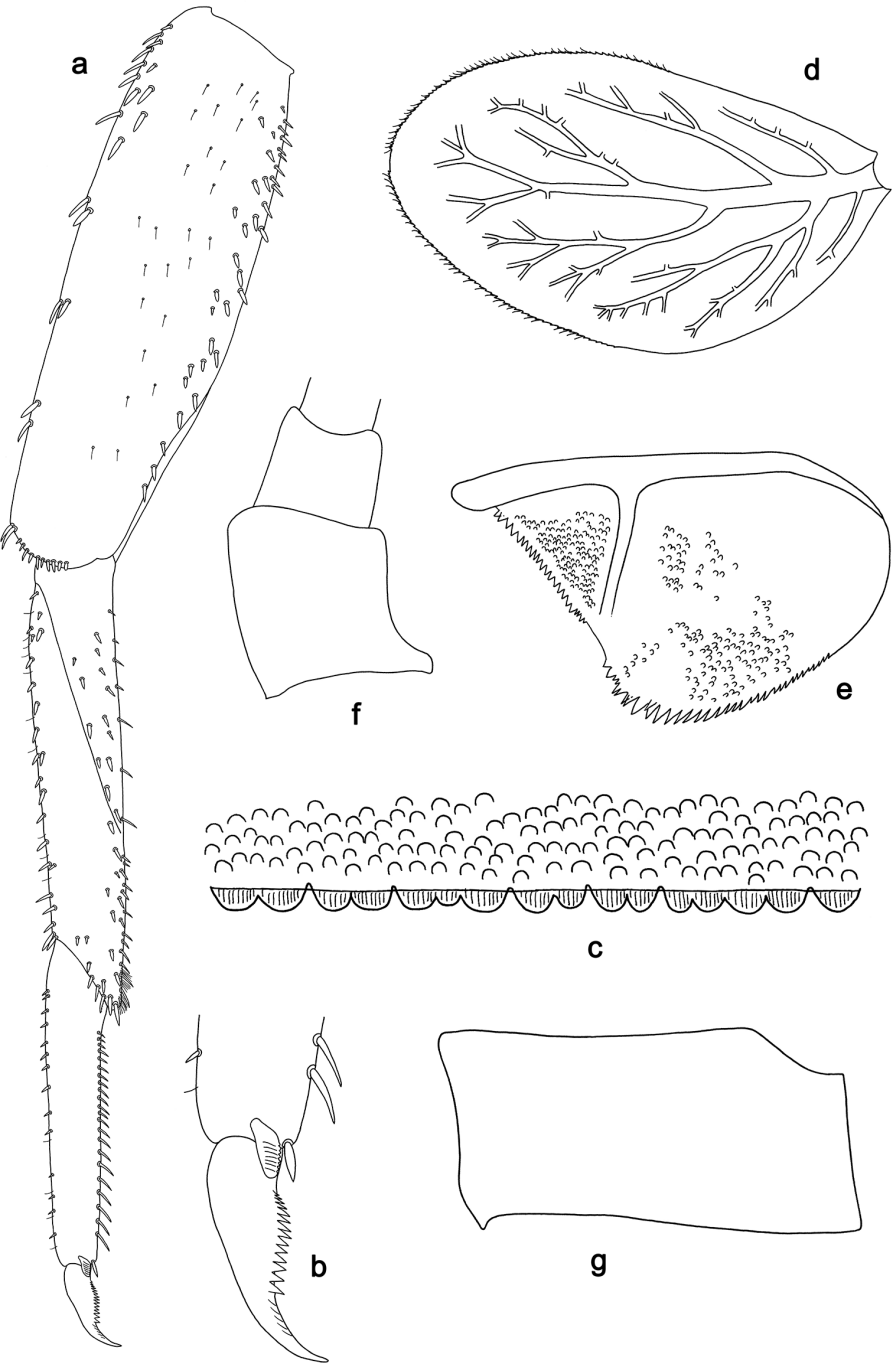
*Labiobaetis acei* sp. nov., larva morphology **a** foreleg **b** fore claw **c** tergum IV **d** gill IV **e** paraproct **f** antennal scape **g** metanotum.

Figures 5 , 6 , 41b , 46a , 48c

##### Diagnosis.

**Larva.** Following combination of characters: A) dorsal surface of labrum with submarginal arc of six or seven long, simple setae; B) labial palp segment II with a thumb-like distomedial protuberance, segment III conical; C) left mandible with a comb-shaped structure at base of mola; D) fore femur rather broad, length ca. 3 × maximum width, dorsal margin with 9–12 curved, spine-like setae and a partial second row near margin; E) tergum IV with rounded, partly fused spines at posterior margin, surface with irregular, dense rows of U-shaped scale bases; F) paraproct distally not expanded, with 29–34 stout, marginal spines.

##### Description.

**Larva** (Figs 5, 6, 41b, 46a). Body length 4.2–10.3 mm. Cerci ca. 2/3 of body length. Paracercus ca. 2/3 of cerci length. Antenna approx. twice as long as head length.

***Colouration*.** Head, thorax, and abdomen dorsally brown, with pattern as in Fig. 41b. Fore protoptera brown with dark brown striation. Head, thorax, and abdomen ventrally brown, abdominal segment IX ecru (Fig. 46a). Legs light brown, femur with a distomedial brown spot and a brown apex. Caudalii light brown with a dark brown band at ca. 1/3 of cerci length.

***Antenna*** (Fig. 6f) with scape and pedicel subcylindrical, without distolateral process at scape.

***Labrum*** (Fig. 5a). Rectangular, length 0.7 × maximum width. Distal margin with medial emargination and a small process. Dorsally with medium, fine, simple setae scattered over surface; submarginal arc of setae composed of one plus six or seven long, simple setae, the first two setae after the submedian seta are close together. Ventrally with marginal row of setae composed of lateral and anterolateral long, feathered setae and medial long, bifid, pectinate setae; ventral surface with ca. five short, spine-like setae near lateral and anterolateral margin.

***Right mandible*** (Fig. 5b, c). Incisor and kinetodontium fused. Incisor with five denticles; kinetodontium with three denticles, inner margin of innermost denticle with a row of thin setae. Prostheca robust, apically denticulate. Margin between prostheca and mola with a pronounced hump. Tuft of setae at apex of mola present and many thin setae distally at base of mola.

***Left mandible*** (Fig. 5d–g). Incisor and kinetodontium fused. Incisor with three denticles; kinetodontium with three denticles. Prostheca robust, apically with small denticles and comb-shaped structure. Margin between prostheca and mola slightly convex, with minute denticles toward subtriangular process. Subtriangular process long and slender, above level of area between prostheca and mola. Denticles of mola apically constricted. Tuft of setae at apex of mola present. Comb-shaped structure at base of mola present.

Both mandibles with lateral margins almost straight. Basal half with fine, simple setae scattered over dorsal surface.

***Hypopharynx and superlinguae*** (Fig. 5h). Lingua approx. as long as superlinguae. Lingua longer than broad; medial tuft of stout setae well developed, short; distal half laterally expanded. Superlinguae distally rounded; lateral margin rounded; fine, long, simple setae along distal margin.

***Maxilla*** (Fig. 5i). Galea-lacinia ventrally with two simple, apical setae under canines. Inner dorsal row of setae with three denti-setae, distal denti-seta tooth-like, middle and proximal denti-setae slender, bifid and pectinate. Medially with one bipectinate, spine-like seta and 5–7 medium to long, simple setae. Maxillary palp 1.4 × as long as length of galea-lacinia; 2-segmented; palp segment II 1.4 × length of segment I; setae on maxillary palp fine, simple, scattered over surface of segments I and II; apex of last segment slightly pointed, without excavation at inner distolateral margin.

***Labium*** (Fig. 5j). Glossa basally broad, narrowing toward apex; shorter than paraglossa; inner margin with ca. ten short, stout, spine-like setae plus distalmost one much longer, less robust, spine-like seta; apex with two long and one medium, robust, pectinate setae and one short, robust seta; outer margin with six spine-like setae increasing in length distally; ventral surface with fine, simple, scattered setae. Paraglossa sub-rectangular, curved inward; apex rounded; with three rows of long, robust, distally pectinate setae in apical area and four or five medium, simple setae in anteromedial area; outer margin with some long, spine-like setae; dorsally with a row of six long, spine-like setae near inner margin. Labial palp with segment I 0.7 × length of segments II and III combined. Segment I ventrally with short, fine, simple setae. Segment II with thumb-like distomedial protuberance; distomedial protuberance 0.5 × width of base of segment III; ventral surface with short, fine, simple setae; dorsally with a row of 7–9 medium, spine-like, simple setae near outer margin. Segment III conical; apex slightly pointed; length 1.0 × width; ventrally covered with short, spine-like, simple setae and short, fine, simple setae.

***Hind protoptera*** (Fig. 6g) minute.

***Foreleg*** (Fig. 6a, b). Ratio of foreleg segments 1.4:1.0:0.8:0.2. ***Femur*.** Length ca. 3 × maximum width. Dorsal margin with a row of 9–12 curved, spine-like setae, a partial row of spine-like setae and some additional spine-like setae near margin; length of setae 0.16 × maximum width of femur. Apex rounded, with a pair of curved, spine-like setae and some short, stout setae. Many stout, lanceolate setae scattered along ventral margin; femoral patch absent. ***Tibia*.** Dorsal margin with a row of short to medium, spine-like setae and fine, simple setae, on apex two longer, spine-like setae. Ventral margin with a row of short to medium curved, spine-like setae, distally of patellotibial suture one longer, curved, spine-like seta, on apex some longer, partly bipectinate, spine-like setae and a tuft of fine, simple setae. Anterior surface scattered with stout, lanceolate setae. Patellotibial suture present on basal half. ***Tarsus*.** Dorsal margin with a row of short, spine-like setae and fine, simple setae. Ventral margin with a row of curved, spine-like setae. Claw with one row of 12–14 denticles; distally pointed; with ca. five stripes; subapical setae absent.

***Terga*** (Fig. 6c). Surface with dense, irregular rows of U-shaped scale bases. Posterior margin of tergum IV with rounded, partly fused spines, wider than long.

***Gills*** (Fig. 6d). Present on segments II–VII. Margin with small denticles intercalating fine simple setae. Tracheae extending from main trunk to inner and outer margins. Gill IV as long as length of segments V and VI combined. Gill VII as long as length of segments VIII and half of IX combined.

***Paraproct*** (Fig. 6e). Distally not expanded, with 29–34 stout, marginal spines. Surface scattered with U-shaped scale bases. Cercotractor with numerous small, marginal spines.

##### Etymology.

Dedicated to Mr. Ace Kevin Amarga (Philippines/Taiwan), an outstanding collector and entomologist, for loaning some precious material to one of the authors (JG).

##### Distribution.

Philippines: Luzon (Fig. 48c).

##### Biological aspects.

The specimens were collected at altitudes of 1700 m and 1820 m, either on rock surface or bottom gravel in riffles or runs, or in root packs or grass bunches in riffles or runs.

##### Type material.

***Holotype*.** Philippines • larva; Luzon, Mountain Province, Bauko, mineral rich mountain creek; 17°03'53"N, 121°05'10"E; 1820 m; XI.1997; leg. Mey; on slide; GenBank: MT830941; GBIFCH 00763643; PNM. ***Paratypes*.** Philippines • 41 larvae; same data as holotype; 2 on slides; GBIFCH 00592360, GBIFCH 00592361; MZL; 13 in alcohol; GBIFCH 00515445; MZL; 25 in alcohol; GBIFCH 00515449; AdMU; 1 in alcohol; GBIFCH 00515452; MZL • 64 larvae; Luzon, Ifugao, Banaue, Sumigar Bridge; 16°59'37"N, 121°02'51"E; 1700 m; IX.1997; leg. Mey; 3 on slides; GenBank: MT830942, MT830943; GBIFCH 00592329, GBIFCH 00763645, GBIFCH 00763651; MZL; 31 in alcohol; GBIFCH 00515446, GBIFCH 00515447, GBIFCH 00515453; MZL; 30 in alcohol; GBIFCH 00515448, GBIFCH 00515450; AdMU.

#### Labiobaetis aldabae sp. nov.

Taxon classificationAnimaliaEphemeropteraBaetidae

80A0EC53-D5F9-5327-A7B1-DEB91BA054E7

http://zoobank.org/9B9F4A30-2C5A-4B83-9649-175F52920FC1

**Figure 7.**
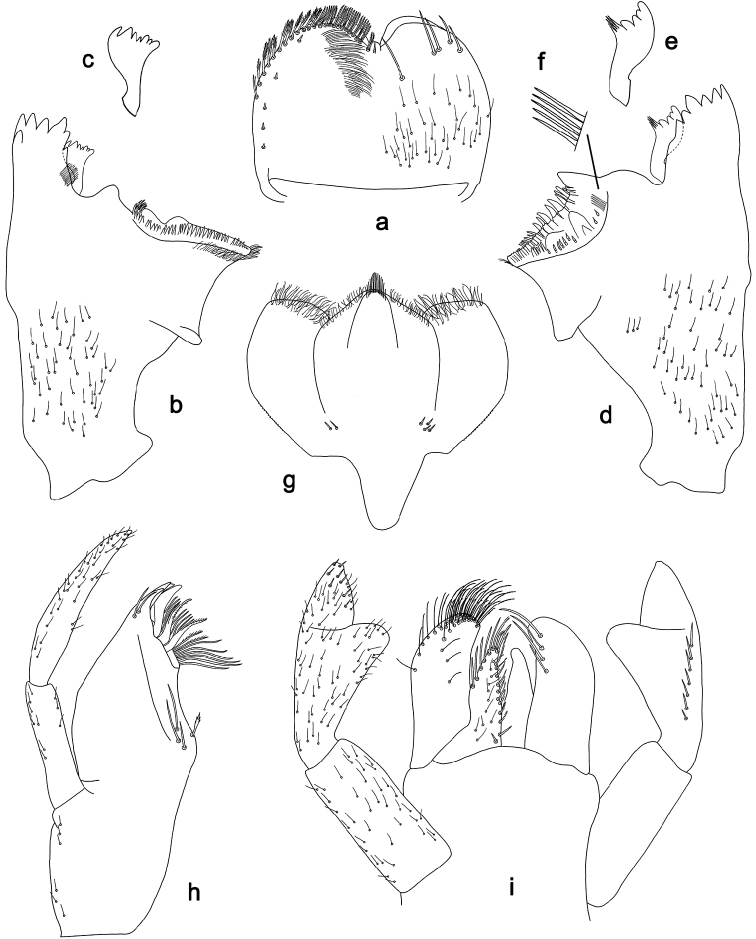
*Labiobaetis aldabae* sp. nov., larva morphology **a** labrum **b** right mandible **c** right prostheca **d** left mandible **e** left prostheca **f** comb-shaped structure at left mola **g** hypopharynx and superlinguae **h** maxilla **i** labium.

**Figure 8.**
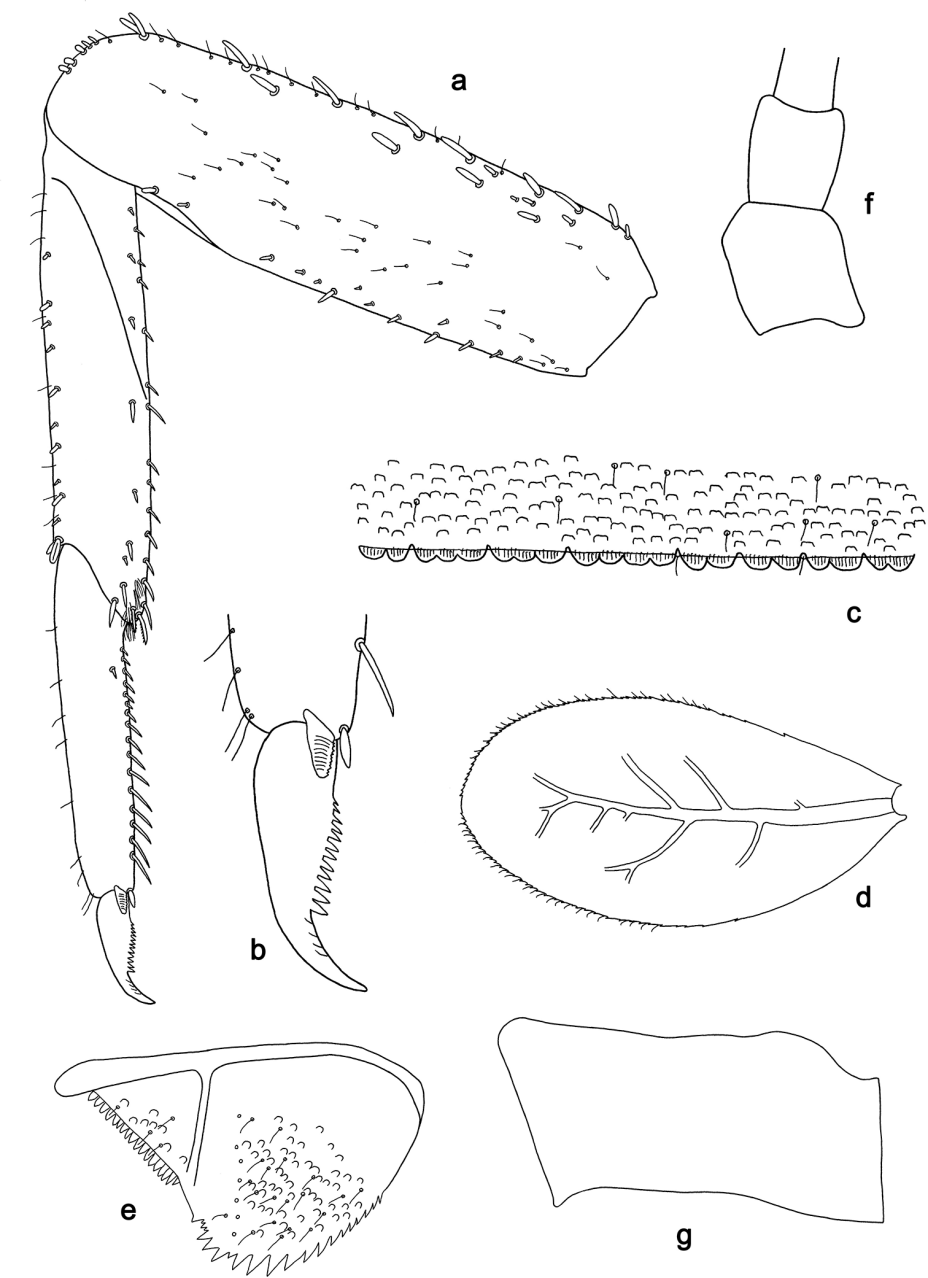
*Labiobaetis aldabae* sp. nov., larva morphology **a** foreleg **b** fore claw **c** tergum IV **d** gill IV **e** paraproct **f** antennal scape **g** metanotum.

Figures 7 , 8 , 41c , 46b , 48c

##### Diagnosis.

**Larva.** Following combination of characters: A) dorsal surface of labrum with submarginal arc of 4–6 long, simple setae; B) labial palp segment II with a thumb-like distomedial protuberance, segment III conical; C) left mandible with a comb-shaped structure at base of mola; D) fore femur rather broad, length 3.4 × maximum width, with ca. nine curved, spine-like setae and a partial second row near margin; E) claw with 12–15 denticles; F) paraproct distally not expanded, with 18–21 stout, marginal spines.

##### Description.

**Larva** (Figs 7, 8, 41c, 46b). Body length 4.1–5.4 mm. Cerci ca. 2/3 of body length. Paracercus ca. 2/3 of cerci length. Antenna approx. twice as long as head length.

***Colouration*.** Head dorsally light brown, thorax and abdomen dorsally brown with bright pattern as in Fig. 41c. Fore protoptera light brown with darker and brighter striation. Head, thorax, and abdomen ventrally brown with bright pattern as in Fig. 46b. Femur with a distomedial brown spot and a distodorsal brown streak, apically brown, tibia partially light brown, tarsus light brown, apically darker. Caudalii light brown with a brown dark band at ca. 1/3 of cerci length, distal area of cerci brown.

***Antenna*** (Fig. 8f) with scape and pedicel subcylindrical, without distolateral process at scape.

***Labrum*** (Fig. 7a). Rectangular, length 0.7 × maximum width. Distal margin with medial emargination and a small process. Dorsally with medium, fine, simple setae scattered over surface; submarginal arc of setae composed of one plus 4–6 long, simple setae, the first two setae after the submedian seta are close together. Ventrally with marginal row of setae composed of lateral and anterolateral long, feathered setae and medial long, bifid, pectinate setae; ventral surface with ca. five short, spine-like setae near lateral and anterolateral margin.

***Right mandible*** (Fig. 7b, c). Incisor and kinetodontium fused. Incisor with four denticles; kinetodontium with three denticles, inner margin of innermost denticle with a row of thin setae. Prostheca robust, apically denticulate. Margin between prostheca and mola with a pronounced hump. Tuft of setae at apex of mola present and many thin setae distally at base of mola.

***Left mandible*** (Fig. 7d–f). Incisor and kinetodontium fused. Incisor with three denticles; kinetodontium with three denticles. Prostheca robust, apically with small denticles and comb-shaped structure. Margin between prostheca and mola convex. Subtriangular process long and slender, above level of area between prostheca and mola. Denticles of mola apically constricted. Tuft of setae at apex of mola present. Comb-shaped structure at base of mola present.

Both mandibles with lateral margins almost straight. Basal half with fine, simple setae scattered over dorsal surface.

***Hypopharynx and superlinguae*** (Fig. 7g). Lingua approx. as long as superlinguae. Lingua longer than broad; medial tuft of stout setae well developed, short; distal half not expanded. Superlinguae distally rounded; lateral margin rounded; fine, long, simple setae along distal margin.

***Maxilla*** (Fig. 7h). Galea-lacinia ventrally with two simple, apical setae under canines. Inner dorsal row of setae with three denti-setae, distal denti-seta tooth-like, middle and proximal denti-setae slender, bifid and pectinate. Medially with one bipectinate, spine-like seta and three or four medium to long, simple setae. Maxillary palp 1.4 × as long as length of galea-lacinia; 2-segmented; palp segment II 1.6 × length of segment I; setae on maxillary palp fine, simple, scattered over surface of segments I and II; apex of last segment slightly pointed, without excavation at inner distolateral margin.

***Labium*** (Fig. 7i). Glossa basally broad, narrowing toward apex; shorter than paraglossa; inner margin with ca. ten short, stout, spine-like setae plus distalmost one much longer, less robust, spine-like seta; apex with two long and one medium, robust, pectinate setae; outer margin with five long, spine-like setae; ventral surface with fine, simple, scattered setae. Paraglossa sub-rectangular, curved inward; apex rounded; with three rows of long, robust, distally pectinate setae in apical area and two or three medium, simple setae in anteromedial area; outer margin with some long, spine-like setae; dorsally with a row of four long, spine-like, simple setae near inner margin. Labial palp with segment I 0.8 × length of segments II and III combined. Segment I ventrally with short, fine, simple setae. Segment II with thumb-like distomedial protuberance; distomedial protuberance 0.7 × width of base of segment III; ventral surface with short, fine, simple setae; dorsally with a row of six or seven long, spine-like, simple setae near outer margin. Segment III conical; apex slightly truncate; length 1.3 × width; ventrally covered with short, spine-like, simple setae and short, fine, simple setae.

***Hind protoptera*** (Fig. 8g) minute.

***Foreleg*** (Fig. 8a, b). Ratio of foreleg segments 1.3:1.0:0.7:0.3. ***Femur*.** Length ca. 3 × maximum width. Dorsal margin with a row of ca. nine curved, spine-like setae, a partial row of spine-like setae near margin; length of setae 0.19 × maximum width of femur. Apex rounded, with a pair of curved, spine-like setae and some short, stout setae. Many stout, lanceolate setae scattered along ventral margin; femoral patch absent. ***Tibia*.** Dorsal margin with a row of short, spine-like setae and fine, simple setae, on apex one longer, spine-like seta. Ventral margin with a row of short to medium curved, spine-like setae, distally of patellotibial suture one longer, curved, spine-like seta, on apex some longer, partly bipectinate, spine-like setae and a tuft of fine, simple setae. Anterior surface scattered with stout, lanceolate setae. Patellotibial suture present on basal half. ***Tarsus*.** Dorsal margin with a row of fine, simple setae. Ventral margin with a row of curved, spine-like setae. Claw with one row of 12–15 denticles on Luzon Island and ca. 17 denticles on Negros Island; distally pointed; with 4–7 stripes; subapical setae absent.

***Terga*** (Fig. 8c). Surface with irregular rows of U-shaped scale bases and scattered fine, simple setae. Posterior margin of tergum IV with rounded, partly fused spines, wider than long.

***Gills*** (Fig. 8d). Present on segments II–VII. Margin with small denticles intercalating fine simple setae. Tracheae extending from main trunk to inner and outer margins. Gill IV as long as length of segments V and 3/4 VI combined. Gill VII as long as length of segments VIII and ½ IX combined.

***Paraproct*** (Fig. 8e). Distally not expanded, with 18–21 stout, marginal spines. Surface scattered with U-shaped scale bases and fine, simple setae. Cercotractor with numerous small, marginal spines.

##### Etymology.

Dedicated to Ms. Kyra Mari Dominique Aldaba (Philippines), member of the AdMU Biodiversity Laboratory.

##### Distribution.

Philippines: Luzon and Negros (Fig. 48c).

##### Biological aspects.

The specimens were collected at altitudes from 50 m to 1400 m, mainly in riverside pools, but also in root packs or grass bunches in the runs.

##### Type material.

***Holotype*.** Philippines • larva; Luzon, Laguna, Samil River; 14°08'N, 121°31'E; 370 m; 27.VI.2018; leg. BIO-PHIL exped.; on slide; GenBank: MT830944; GBIFCH 00654913; PNM. ***Paratypes*.** Philippines • 2 larvae; same data as holotype; 1 on slide; GBIFCH 00592273; ZSM; 1 in alcohol, GBIFCH 00515409; ZSM • 1 larva; Luzon, Nueva Ecija, Pantabangan, Candaclan River; 240 m, 15°46'48"N, 121°13'17"E; 240 m; 05.II.1998; leg. Mendoza; on slide; GenBank: MT830945; GBIFCH 00654908; ZSM • 39 larvae; Luzon, Benguet, Tuba, Taloy Sur; 16°21'33"N, 120°30'31"E; 400 m; XI.1997; leg. Mey; 1 on slide; GenBank: MT830947; GBIFCH 00763648; AdMU; 38 in alcohol; GBIFCH 00515443; ZSM • 126 larvae; Luzon, Benguet, Kabayan, Bongis Bridge; 16°34'11"N, 120°50'12"E; 1000 m; XI.1997; leg. Mey; 1 on slide; GenBank: MT830946; GBIFCH 00763646; AdMU; 66 in alcohol; GBIFCH 00515440; 59 in alcohol; GBIFCH 00515500, GBIFCH 00515464, GBIFCH 00515441; ZSM • 1 larva; Negros Oriental, Valencia, Malaunay, small river; 09°18'17"N, 123°10'07"E; 470 m; 01.IX.2019; leg. Garces and Pelingen; on slide; GenBank: MT830948; GBIFCH 00654889; ZSM.

##### Other material.

Philippines • 27 larvae; Luzon, Ifugao, Tinoc; 16°40'58"N, 120°56'59"E; 1400 m; XI.1997; leg. Mey; in alcohol; GBIFCH 00515444; AdMU • 4 larvae; Luzon, Ilocos Sur, Suyo Municipality, Tagudin-Cervantes-Sabangan Road, Besang Pass Area; 16°57'17"N, 120°38'52"E; 1200 m; 15.IV.2019; leg. Freitag, Garces and Pangantihon; in alcohol; GBIFCH 00515442; ZSM.

#### Labiobaetis camiguinensis sp. nov.

Taxon classificationAnimaliaEphemeropteraBaetidae

27A9D314-54E8-55F7-AC61-FA0A1A574977

http://zoobank.org/A5674916-368A-4191-8F11-898F6CB52162

**Figure 9.**
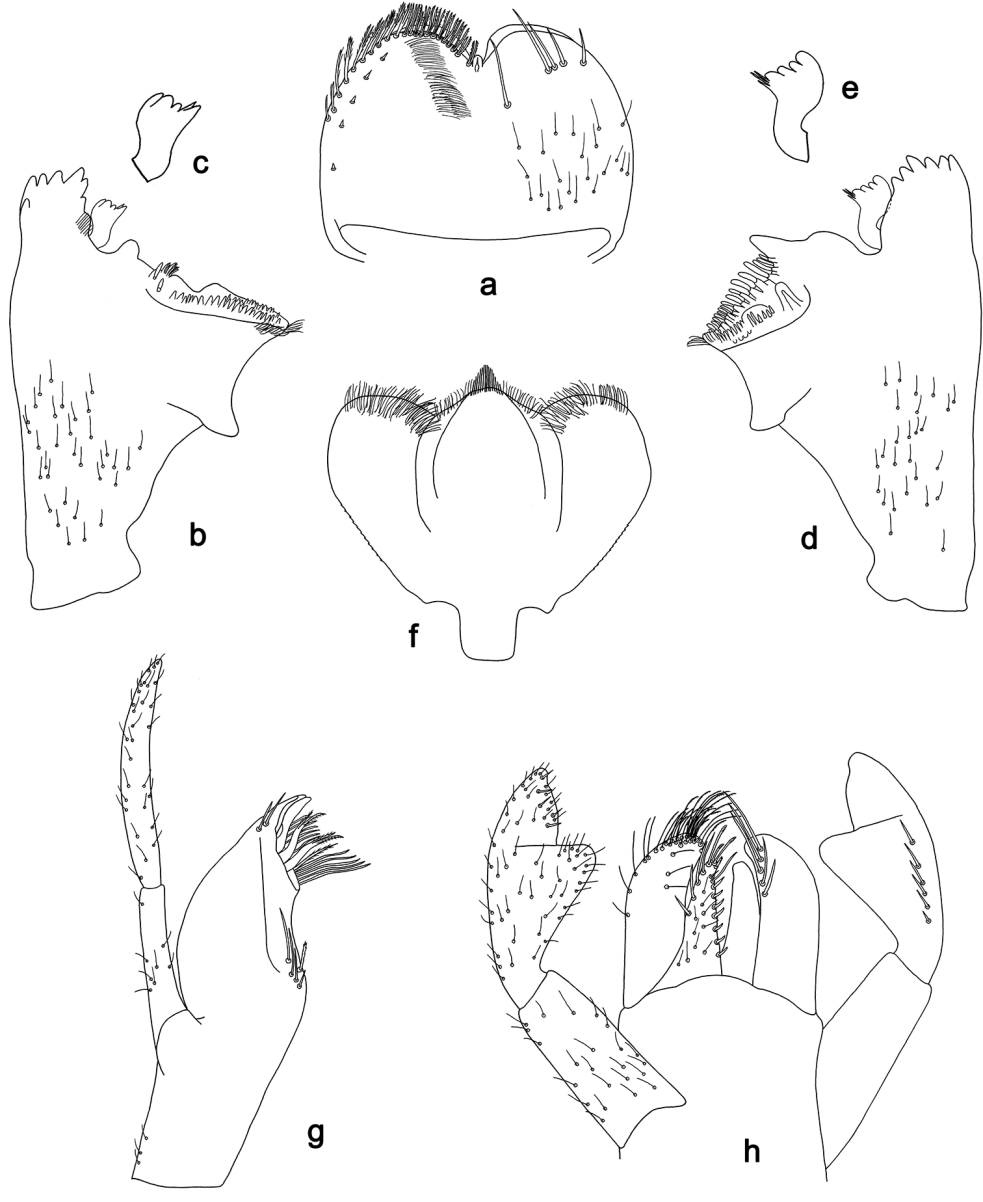
*Labiobaetis camiguinensis* sp. nov., larva morphology **a** labrum **b** right mandible **c** right prostheca **d** left mandible **e** left prostheca **f** hypopharynx and superlinguae **g** maxilla **h** labium.

**Figure 10.**
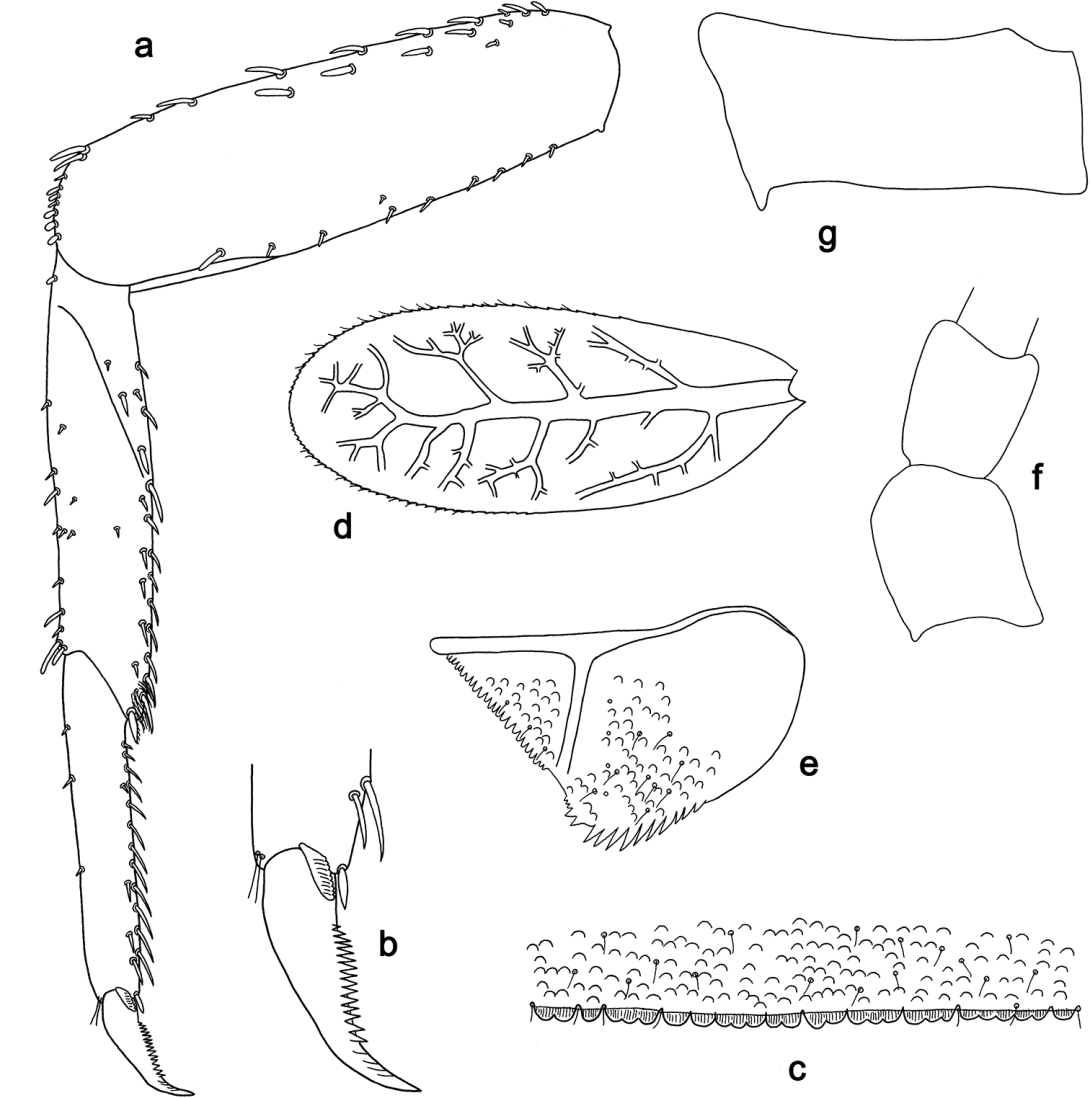
*Labiobaetis camiguinensis* sp. nov., larva morphology **a** foreleg **b** fore claw **c** tergum IV **d** gill IV **e** paraproct **f** antennal scape **g** metanotum.

Figures 9 , 10 , 42a , 46c , 47d , 48c

##### Diagnosis.

**Larva.** Following combination of characters: A) dorsal surface of labrum with submarginal arc of one plus four or five long, simple setae; B) labial palp segment II with thumb-like distomedial protuberance; C) fore femur rather broad, length ca. 3 × maximum width, dorsal margin with ca. ten curved, spine-like setae and a partial second row near margin; D) claw with 14 or 15 denticles; E) paraproct distally not expanded, with 15–17 stout, marginal spines.

##### Description.

**Larva** (Figs 9, 10, 42a, 46c, 47d). Body length 3.6–3.9 mm; antenna: approx. twice as long as head length.

***Colouration*.** Head dorsally light brown, thorax and abdomen dorsally brown with bright pattern as in Fig. 42a. Fore protoptera light brown with brown striation. Head, thorax, and abdomen ventrally brown with bright pattern as in Fig. 46c. Femur ecru with distomedial brown spot, apex with brown spot, tibia and tarsus light brown. Caudalii light brown, with a brown band at ca. 1/3 of cerci.

***Antenna*** (Fig. 10f) with scape and pedicel subcylindrical, without distolateral process at scape.

***Labrum*** (Fig. 9a). Rectangular, length 0.7 × maximum width. Distal margin with medial emargination and a small process. Dorsally with medium, fine, simple setae scattered over surface; submarginal arc of setae composed of one plus four or five long, simple setae, the first two setae after the submedian seta are close together. Ventrally with marginal row of setae composed of lateral and anterolateral long, feathered setae and medial long, bifid, pectinate setae; ventral surface with ca. five short, spine-like setae near lateral and anterolateral margin.

***Right mandible*** (Fig. 9b, c). Incisor and kinetodontium fused. Incisor with four denticles; kinetodontium with three denticles, inner margin of innermost denticle with a row of thin setae. Prostheca robust, apically denticulate. Margin between prostheca and mola with a pronounced hump. Tuft of setae at apex of mola present and many thin setae distally at base of mola.

***Left mandible*** (Fig. 9d, e). Incisor and kinetodontium fused. Incisor with three denticles; kinetodontium with three denticles. Prostheca robust, apically with small denticles and comb-shaped structure. Margin between prostheca and mola convex. Subtriangular process long and slender, above level of area between prostheca and mola. Denticles of mola apically constricted. Tuft of setae at apex of mola present.

Both mandibles with lateral margins almost straight. Basal half with fine, simple setae scattered over dorsal surface.

***Hypopharynx and superlinguae*** (Figs 9f, 47d). Lingua approx. as long as superlinguae. Lingua approx. as broad as long; medial tuft of stout setae well developed, short; distal half not expanded. Superlinguae distally rounded; lateral margin angulate; fine, long, simple setae along distal margin.

***Maxilla*** (Fig. 9g). Galea-lacinia ventrally with two simple, apical setae under canines. Inner dorsal row of setae with three denti-setae, distal denti-seta tooth-like, middle and proximal denti-setae slender, bifid and pectinate. Medially with one bipectinate, spine-like seta and three or four medium to long, simple setae. Maxillary palp 1.4 × as long as length of galea-lacinia; 2-segmented; palp segment II 1.8 × length of segment I; setae on maxillary palp fine, simple, scattered over surface of segments I and II; apex of last segment slightly pointed, without excavation at inner distolateral margin.

***Labium*** (Fig. 9h). Glossa basally broad, narrowing toward apex; shorter than paraglossa; inner margin with ca. seven short, stout, spine-like setae plus distalmost one much longer, less robust, spine-like seta; apex with two long and one medium, robust, pectinate setae and one short, robust seta; outer margin with four spine-like setae increasing in length distally; ventral surface with fine, simple, scattered setae. Paraglossa sub-rectangular, curved inward; apex rounded; with three rows of long, robust, distally pectinate setae in apical area and a row of three medium, simple setae in anteromedial area; outer margin with some long, spine-like setae; dorsally with a row of five long, spine-like, simple setae near inner margin. Labial palp with segment I 0.7 × length of segments II and III combined. Segment I ventrally with short, fine, simple setae. Segment II with thumb-like distomedial protuberance; distomedial protuberance 0.6 × width of base of segment III; ventral surface with short, fine, simple setae; dorsally with a row of six short to medium, spine-like, simple setae near outer margin. Segment III conical; apex rounded; length 1.2 × width; ventrally covered with short, spine-like, simple setae and short, fine, simple setae.

***Hind protoptera*** (Fig. 10g) minute.

***Foreleg*** (Fig. 10a, b). Ratio of foreleg segments 1.4:1.0:0.8:0.3. ***Femur*.** Length ca. 3 × maximum width. Dorsal margin with ca. ten curved, spine-like setae, proximally and medially a partial second row of spine-like setae near margin; length of setae 0.18 × maximum width of femur. Apex rounded, with a pair of spine-like setae and some short, stout setae. Many stout, lanceolate setae scattered along ventral margin; femoral patch absent. ***Tibia*.** Dorsal margin with a row of short to medium, spine-like setae, on apex two longer, spine-like setae. Ventral margin with a row of medium, curved, spine-like setae, distally of patellotibial suture one long, curved, spine-like seta, on apex some longer, partly bipectinate, spine-like setae and a tuft of fine, simple setae. Anterior surface scattered with stout, lanceolate setae. Patellotibial suture present on basal half. ***Tarsus*.** Dorsal margin with some short, stout setae. Ventral margin with a row of curved, spine-like setae and some spine-like setae near margin. Claw with one row of 14 or 15 denticles; distally pointed; with 4–6 stripes; subapical setae absent.

***Terga*** (Fig. 10c). Surface with irregular rows of U-shaped scale bases and scattered fine, simple setae. Posterior margin of tergum IV with rounded, partly fused spines, wider than long.

***Gills*** (Fig. 10d). Present on segments II–VII. Margin with small denticles intercalating fine simple setae. Tracheae extending from main trunk to inner and outer margins. Gill IV as long as length of segments V and VI combined. Gill VII as long as length of segments VIII and IX combined.

***Paraproct*** (Fig. 10e). Distally not expanded, with 15–17 stout, marginal spines. Surface scattered with U-shaped scale bases, fine, simple setae and micropores. Cercotractor with numerous small, marginal spines.

##### Etymology.

Refers to the island Camiguin, where the specimens were collected.

##### Distribution.

Philippines: Camiguin (Fig. 48c).

##### Biological aspects.

The specimens were collected at altitudes of 30 m and 900 m, mainly on bottom gravel.

##### Type material.

***Holotype*.** Philippines • larva; Camiguin, Sagay, Bonbon, lower Binangawan River; 09°06'39"N 124°43'45"E; 30 m; 09.XII.2018; leg. Freitag and Wantzen; on slide; GenBank: MT830949; GBIFCH 00654915; PNM. ***Paratypes*.** Philippines • 2 larvae; same data as holotype; 1 on slide; GBIFCH 00592308; ZSM; 1 in alcohol; GBIFCH 00515489; ZSM • 1 larva; Camiguin, Mt. Mabajao Sagay, upstream Binangawan Falls; 09°09'25"N, 124°43'57"E; 900 m; 09.XII.2018; leg. Freitag; in alcohol; GBIFCH 00515454; AdMU.

#### Labiobaetis lachicae sp. nov.

Taxon classificationAnimaliaEphemeropteraBaetidae

C35E4667-9F8F-59C5-AA3B-D0F58FEF272F

http://zoobank.org/60DC83D6-32F6-4181-B224-1A890EF26245

**Figure 11.**
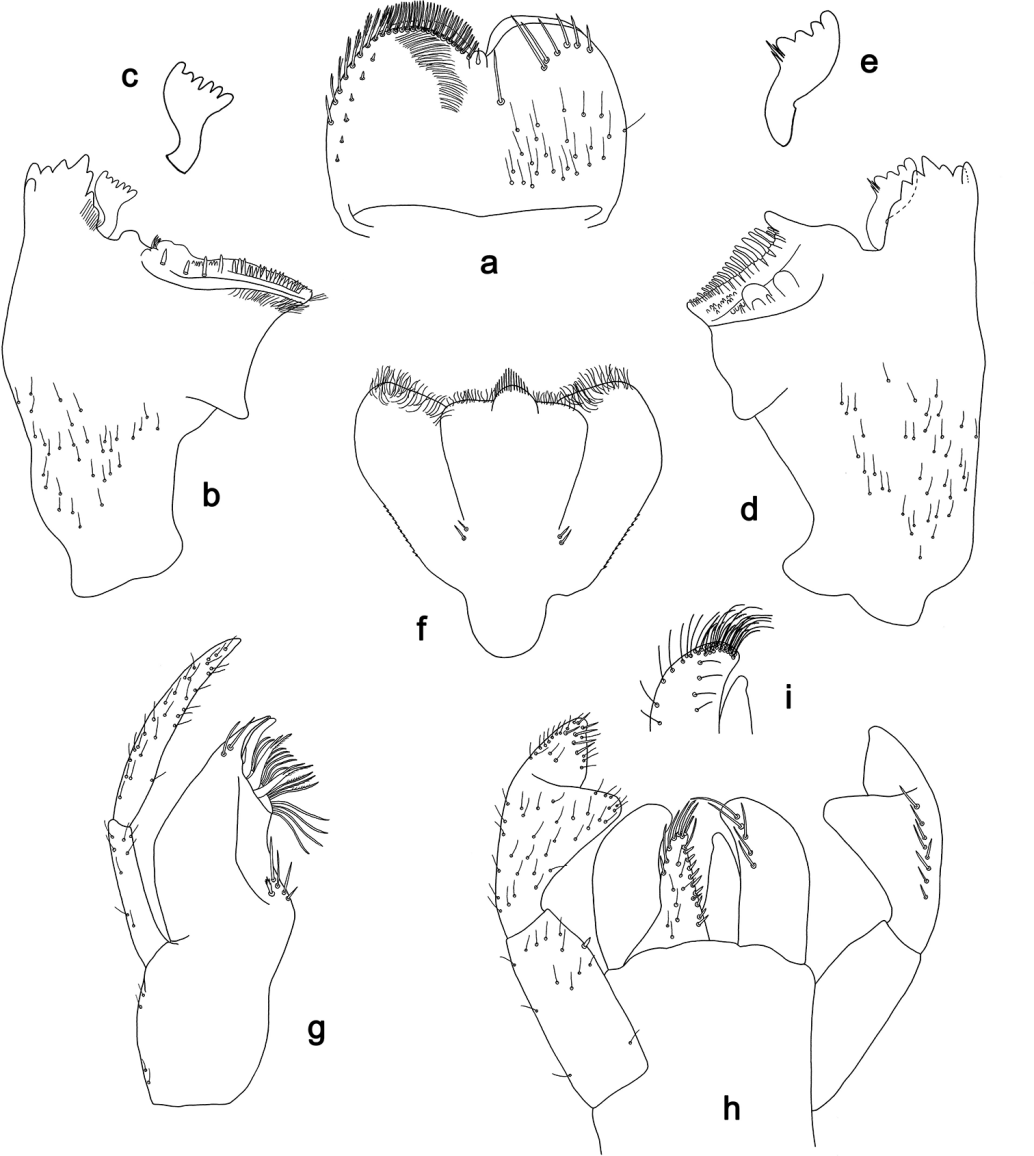
*Labiobaetis lachicae* sp. nov., larva morphology **a** labrum **b** right mandible **c** right prostheca **d** left mandible **e** left prostheca **f** hypopharynx and superlinguae **g** maxilla **h** labium **i** apex of paraglossa.

**Figure 12.**
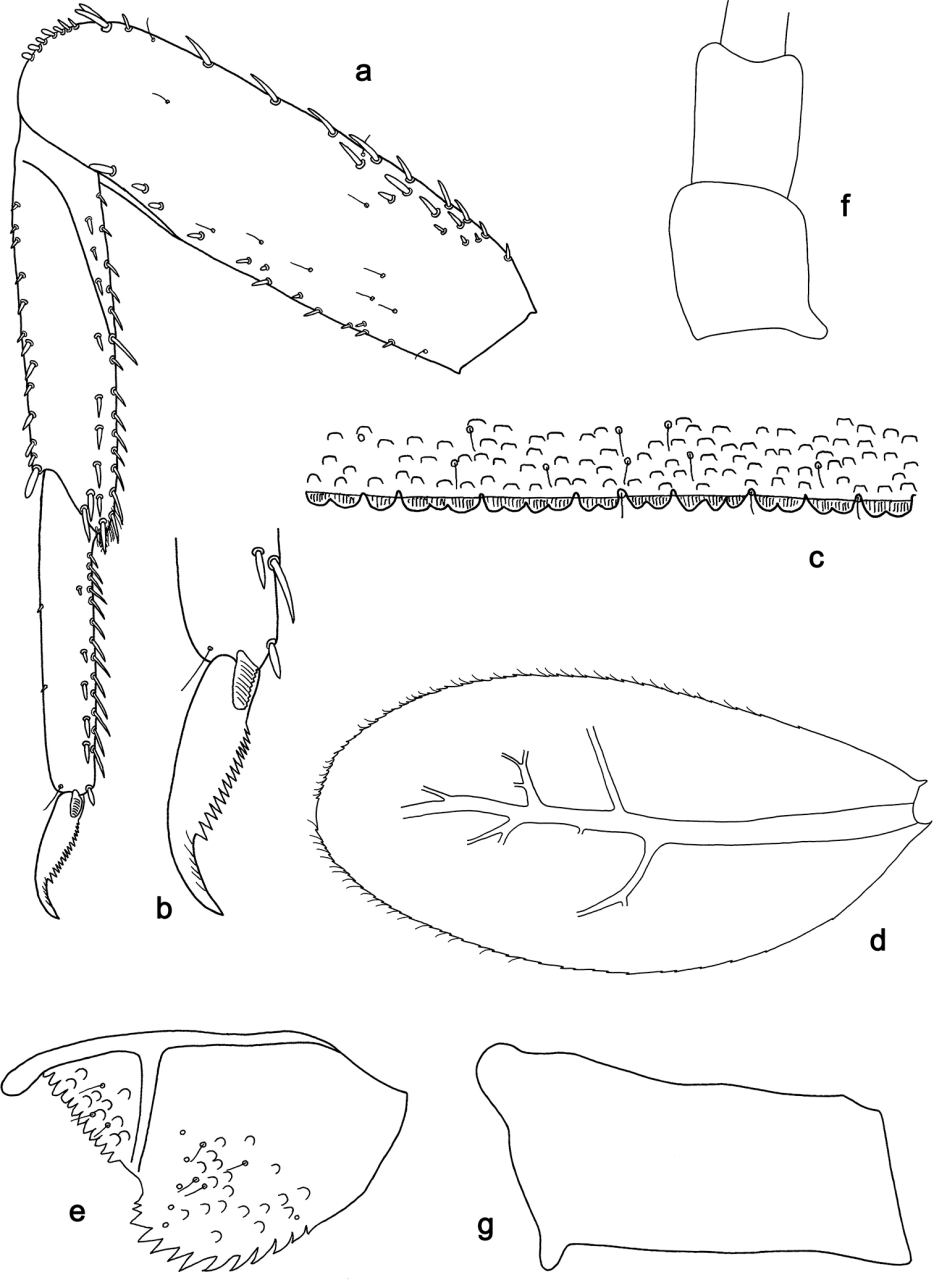
*Labiobaetis lachicae* sp. nov., larva morphology **a** foreleg **b** fore claw **c** tergum IV **d** gill IV **e** paraproct **f** antennal scape **g** metanotum.

Figures 11 , 12 , 42b , 47a , 48c

##### Diagnosis.

**Larva.** Following combination of characters: A) dorsal surface of labrum with submarginal arc of one plus 5–7 long, simple setae; B) labial palp segment II with a thumb-like distomedial protuberance, segment III conical; C) left mandible without setae at apex of mola; D) fore femur rather broad, length ca. 3 × maximum width, dorsal margin with 10–12 curved, spine-like setae and a partial second row near margin; E) claw with 15–17 denticles; F) paraproct distally not expanded, with ca. 13 stout, marginal spines.

##### Description.

**Larva** (Figs 11, 12, 42b, 47a). Body length 3.6–3.7 mm. Cerci ca. 2/3 of body length. Paracercus ca. 2/3 of cerci length. Antenna approx. twice as long as head length.

***Colouration*.** Head dorsally light brown, thorax and abdomen dorsally brown, with bright pattern as in Fig. 42b. Fore protoptera light brown with distinct brown striation. Head, thorax, and abdomen ventrally brown with bright pattern as in Fig. 47a. Legs ecru, femur with two partly merged distomedial brown spots, apex with brown spot, tibia with distomedial brown area. Caudalii light brown, with a brown band at ca. 1/3 of cerci, cerci brown in distal part.

***Antenna*** (Fig. 12f) with scape and pedicel subcylindrical, without distolateral process at scape.

***Labrum*** (Fig. 11a). Rectangular, length 0.7 × maximum width. Distal margin with medial emargination and a small process. Dorsally with medium, fine, simple setae scattered over surface; submarginal arc of setae composed of one plus 5–7 long, simple setae, the first two setae after the submedian seta are close together. Ventrally with marginal row of setae composed of lateral and anterolateral long, feathered setae and medial long, bifid setae; ventral surface with ca. six short, spine-like setae near lateral and anterolateral margin.

***Right mandible*** (Fig. 11b, c). Incisor and kinetodontium fused. Incisor with four denticles; kinetodontium with three denticles, inner margin of innermost denticle with a row of thin setae. Prostheca robust, apically denticulate. Margin between prostheca and mola with a pronounced hump. Tuft of setae at apex of mola present and many thin setae distally at base of mola.

***Left mandible*** (Fig. 11d, e). Incisor and kinetodontium fused. Incisor with three denticles; kinetodontium with three denticles. Prostheca robust, apically with small denticles and comb-shaped structure. Margin between prostheca and mola convex. Subtriangular process long and slender, above level of area between prostheca and mola. Denticles of mola apically constricted. Tuft of setae at apex of mola absent.

Both mandibles with lateral margins almost straight. Basal half with fine, simple setae scattered over dorsal surface.

***Hypopharynx and superlinguae*** (Fig. 11f). Lingua shorter than superlinguae. Lingua longer than broad; medial tuft of stout setae well developed, short; distal half laterally expanded. Superlinguae distally rounded; lateral margin angulate; fine, long, simple setae along distal margin.

***Maxilla*** (Fig. 11g). Galea-lacinia ventrally with two simple, apical setae under canines. Inner dorsal row of setae with three denti-setae, distal denti-seta tooth-like, middle and proximal denti-setae slender, bifid and pectinate. Medially with one bipectinate, spine-like seta and four medium to long, simple setae. Maxillary palp 1.3 × as long as length of galea-lacinia; 2-segmented; palp segment II 1.6 × length of segment I; setae on maxillary palp fine, simple, scattered over surface of segments I and II; apex of last segment slightly pointed, without excavation at inner distolateral margin.

***Labium*** (Fig. 11h, i). Glossa basally broad, narrowing toward apex; shorter than paraglossa; inner margin with ca. nine short, stout, spine-like setae plus distalmost one much longer, less robust, spine-like seta; apex with two long and one medium, robust, pectinate setae; outer margin with four spine-like setae increasing in length distally; ventral surface with fine, simple, scattered setae. Paraglossa sub-rectangular, curved inward; apex rounded; with three rows of long, robust, distally pectinate setae in apical area and four medium, simple setae in anteromedial area; outer margin with some long, spine-like setae; dorsally with a row of five long, spine-like, simple setae near inner margin. Labial palp with segment I 0.9 × length of segments II and III combined. Segment I ventrally with short, fine, simple setae and one short, stout, simple seta at inner margin. Segment II with thumb-like distomedial protuberance; distomedial protuberance 0.8 × width of base of segment III; ventral surface with short, fine, simple setae; dorsally with a row of 6–8 medium, spine-like, simple setae near outer margin. Segment III conical; apex rounded; length 1.1 × width; ventrally covered with short, spine-like, simple setae and short, fine, simple setae.

***Hind protoptera*** (Fig. 12g) minute.

***Foreleg*** (Fig. 12a, b). Ratio of foreleg segments 1.4:1.0:0.8:0.3. ***Femur*.** Length ca. 3 × maximum width. Dorsal margin with 10–12 curved, spine-like setae, proximally and medially a partial second row of spine-like setae and some additional stout setae near margin; length of setae 0.23 × maximum width of femur. Apex rounded, with a pair of spine-like setae and some short, stout setae. Many stout, lanceolate setae scattered along ventral margin; femoral patch absent. ***Tibia*.** Dorsal margin with a row of short to medium, spine-like setae, on apex one longer, spine-like seta. Ventral margin with a row of medium, curved, spine-like setae, distally of patellotibial suture one long, curved, spine-like seta, on apex some longer, partly bipectinate, spine-like setae and a tuft of fine, simple setae. Anterior surface scattered with stout, lanceolate setae. Patellotibial suture present on basal half. ***Tarsus*.** Dorsal margin with some short, stout setae. Ventral margin with a row of curved, spine-like setae and some spine-like setae near margin. Claw with one row of 15–17 denticles; distally pointed; with ca. six stripes; subapical setae absent.

***Terga*** (Fig. 12c). Surface with irregular rows of U-shaped scale bases and scattered fine, simple setae and micropores. Posterior margin of tergum IV with rounded, partly fused spines, wider than long.

***Gills*** (Fig. 12d). Present on segments II–VII. Margin with small denticles intercalating fine simple setae. Tracheae partly extending from main trunk towards outer and inner margins. Gill IV as long as length of segments V and VI combined. Gill VII as long as length of segments VIII and IX combined.

***Paraproct*** (Fig. 12e). Distally not expanded, with ca. 13 stout, marginal spines. Surface scattered with U-shaped scale bases, fine, simple setae and micropores. Cercotractor with small, marginal spines.

##### Etymology.

Dedicated to Ms. Maria Kenosis Lachica (Philippines/Japan), friend of the AdMU Biodiversity Laboratory.

##### Distribution.

Philippines: Mindanao (Fig. 48c).

##### Biological aspects.

The specimens were collected at altitudes from sea level to 120 m, in root packs, grass bunches or submerged wood in riffles or runs.

##### Type material.

***Holotype*.** Philippines • larva; Mindanao, Agusan del Sur, San Francisco, Bayogan, Tagkunayan Creek; 08°28'N, 125°59'E; 120 m; 05.II.1998; leg. Mendoza; on slide; GenBank: MT830950; GBIFCH 00654891; PNM. ***Paratypes*.** Philippines • 2 larvae; same data as holotype; 1 on slide; GBIFCH 00592269; AdMU; 1 on slide; GBIFCH 00592310; ZSM. **Other material.** Philippines • 1 larva; Mindanao, Surigao del Sur, Tandag, middle Tandag River; 30 m; 09°03'33"N, 126°05'57"E; 04.XII.2018; leg. Pangantihon; in alcohol; GBIFCH 00515332; AdMU • 1 larva; Mindanao, Surigao del Sur, Tandag, Pangi, Pangi River; 09°06'18"N, 126°08'53"E; 10 m; 30.XI.2018, leg. Pangantihon; on slide; GBIFCH 515520; ZSM.

#### Labiobaetis palawano sp. nov.

Taxon classificationAnimaliaEphemeropteraBaetidae

779A624D-49E9-5AB5-BE10-592DC157808D

http://zoobank.org/9E057916-4F3E-43F3-9AB7-B89D1A001EEA

**Figure 13.**
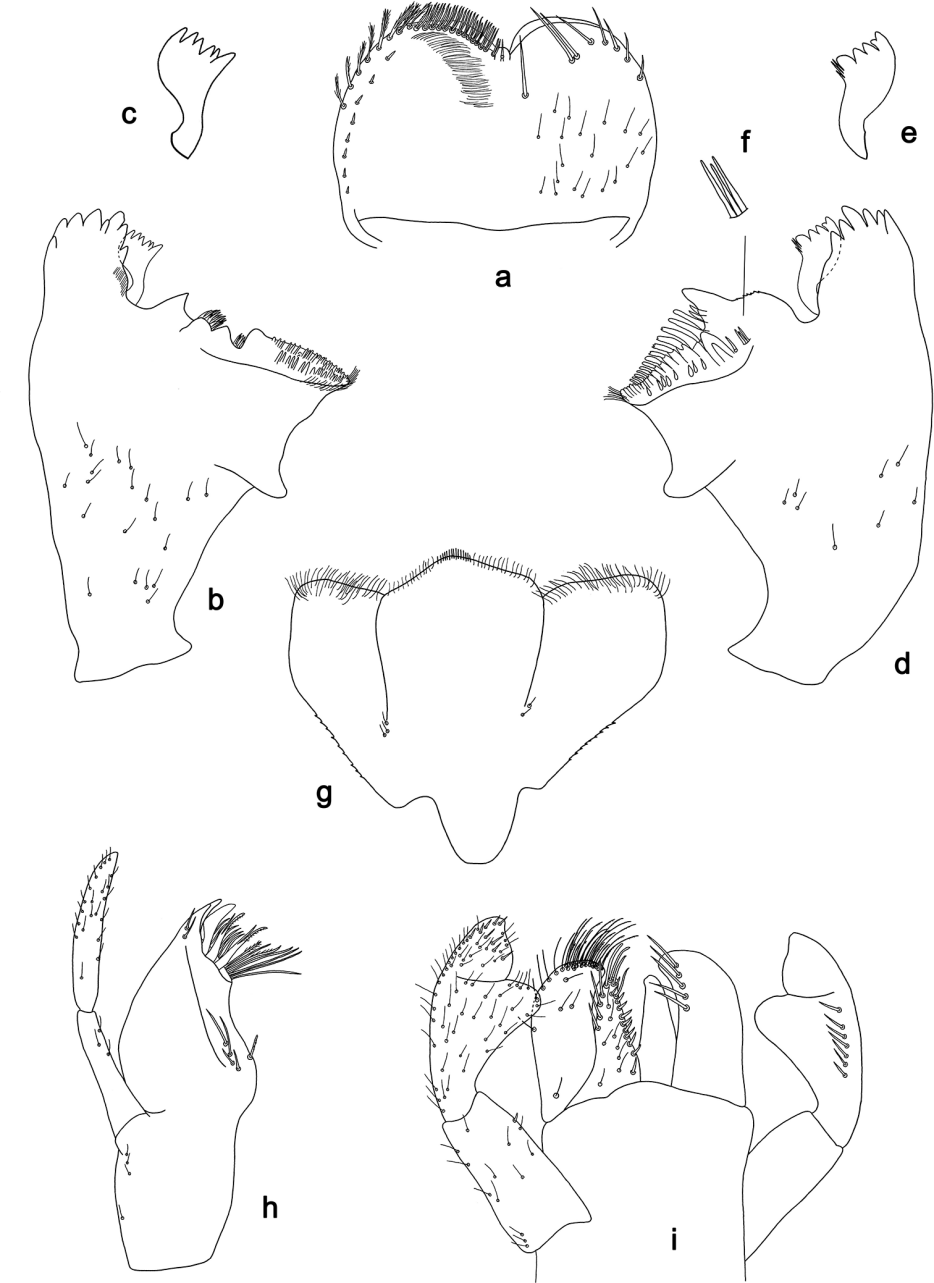
*Labiobaetis palawano* sp. nov., larva morphology **a** labrum **b** right mandible **c** right prostheca **d** left mandible **e** left prostheca **f** comb-shaped structure at left mola **g** hypopharynx and superlinguae **h** maxilla **i** labium.

**Figure 14.**
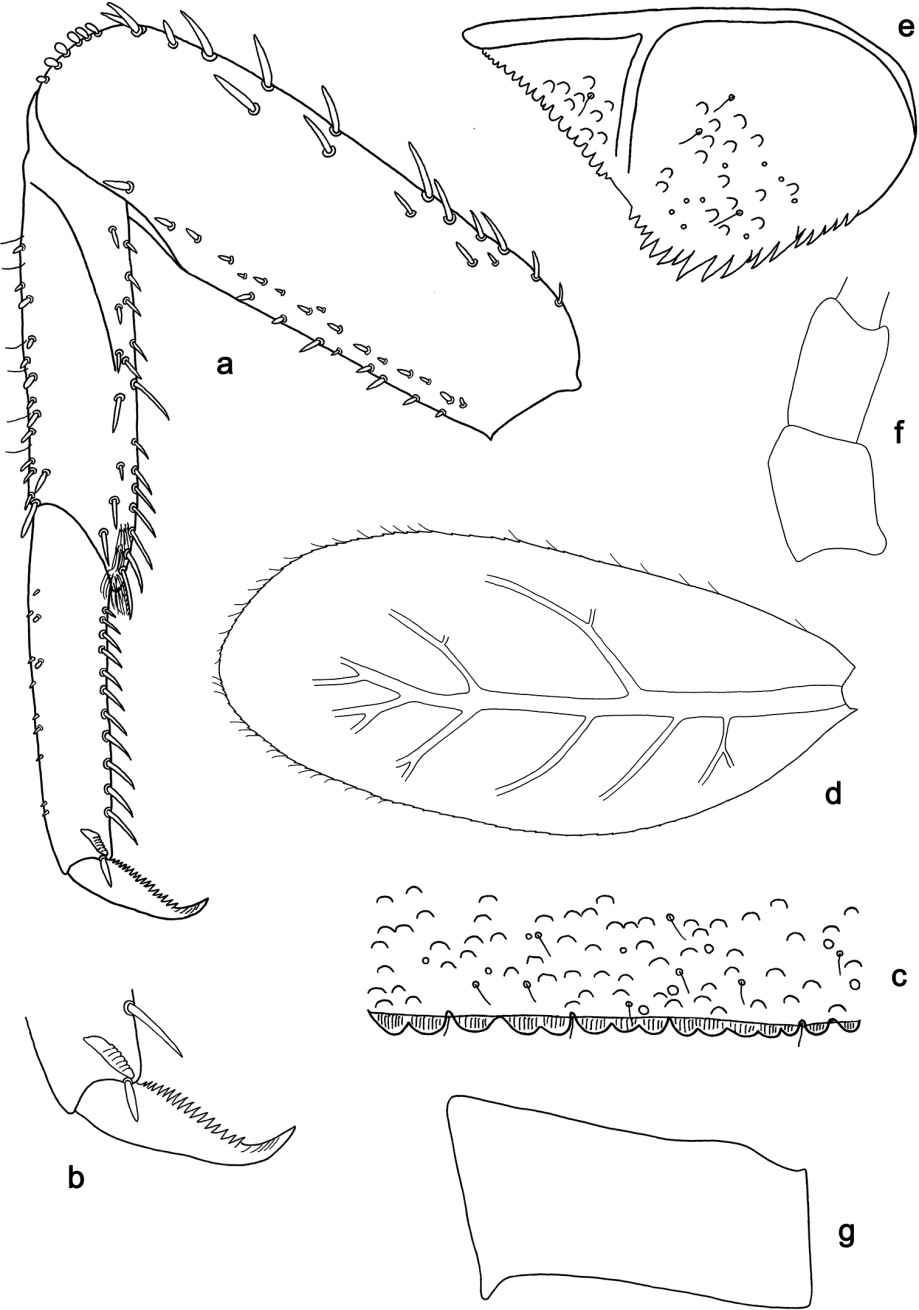
*Labiobaetis palawano* sp. nov., larva morphology **a** foreleg **b** fore claw **c** tergum IV **d** gill IV **e** paraproct **f** antennal scape **g** metanotum.

Figures 13 , 14 , 42c , 47b , 48c

##### Diagnosis.

**Larva.** Following combination of characters: A) dorsal surface of labrum with submarginal arc of one plus seven long, simple setae; B) labial palp segment II with a thumb-like distomedial protuberance, segment III conical; C) mola of right mandible proximally beginning with a double hump; D) hypopharynx with medial tuft of stout setae poorly developed; E) fore femur rather broad, length ca. 3 × maximum width, dorsal margin with 10–13 curved, spine-like setae and a partial second row near margin; F) claw with 15–17 denticles; G) posterior margin of tergum IV with rounded spines, wider than long.

##### Description.

**Larva** (Figs 13, 14, 42c, 47b). Body length 3–4 mm. Cerci ca. 2/3 of body length. Paracercus ca. 2/3 of cerci length. Antenna approx. 2.5 × as long as head length.

***Colouration*.** Head, thorax, and abdomen dorsally brown, with dark brown markings as in Fig. 42c. Fore protoptera light brown with brown striation. Head, thorax, and abdomen ventrally brown. Legs light brown, femur with a distomedial brown spot, apically brown. Caudalii light brown, with a brown band at ca. 1/3 of cerci.

***Antenna*** (Fig. 14f) with scape and pedicel subcylindrical, without distolateral process at scape.

***Labrum*** (Fig. 13a). Rectangular, length 0.7 × maximum width. Distal margin with medial emargination and a small process. Dorsally with medium, fine, simple setae scattered over surface; submarginal arc of setae composed of one plus seven long, simple setae, the first two setae after the submedian seta are close together. Ventrally with marginal row of setae composed of lateral and anterolateral long, feathered setae and medial long, bifid, pectinate setae; ventral surface with ca. eight short, spine-like setae near lateral and anterolateral margin.

***Right mandible*** (Fig. 13b, c). Incisor and kinetodontium fused. Incisor with five denticles; kinetodontium with three denticles, inner margin of innermost denticle with a row of thin setae. Prostheca robust, apically denticulate. Margin between prostheca and mola with a pronounced hump, mola proximally beginning with a double hump. Tuft of setae at apex of mola present and many thin setae distally at base of mola.

***Left mandible*** (Fig. 13d–f). Incisor and kinetodontium fused. Incisor with three denticles; kinetodontium with three denticles. Prostheca robust, apically with small denticles and comb-shaped structure. Margin between prostheca and mola convex, with minute denticles toward subtriangular process. Subtriangular process long and slender, above level of area between prostheca and mola. Denticles of mola apically constricted and partly with minute secondary dentation at the tips. Tuft of setae at apex of mola present. Comb-shaped structure at base of mola present.

Both mandibles with lateral margins slightly convex. Basal half with fine, simple setae scattered over dorsal surface.

***Hypopharynx and superlinguae*** (Fig. 13g). Lingua longer than superlinguae. Lingua longer than broad; medial tuft of stout setae poorly developed; distal half not expanded. Superlinguae distally straight; lateral margin angulate; fine, long, simple setae along distal margin.

***Maxilla*** (Fig. 13h). Galea-lacinia ventrally with two simple, apical setae under canines. Inner dorsal row of setae with three denti-setae, distal denti-seta tooth-like, middle and proximal denti-setae slender, bifid and pectinate. Medially with one bipectinate, spine-like seta and four or five long, simple setae. Maxillary palp 1.2 × as long as length of galea-lacinia; 2-segmented; palp segment II 1.4 × length of segment I; setae on maxillary palp fine, simple, scattered over surface of segments I and II; apex of last segment slightly pointed, without excavation at inner distolateral margin.

***Labium*** (Fig. 13i). Glossa basally broad, narrowing toward apex; shorter than paraglossa; inner margin with ca. nine short, stout, spine-like setae plus distalmost one much longer, less robust, spine-like seta; apex with two long, one medium and one short, robust, pectinate setae; outer margin with four spine-like setae; ventral surface with fine, simple, scattered setae. Paraglossa sub-rectangular, curved inward; apex rounded; with three rows of long, robust, distally pectinate setae in apical area and 3–5 medium, simple setae in anteromedial area and one fine, simple seta in proximomedial area; outer margin with some long, spine-like setae; dorsally with a row of five long, spine-like, simple setae near inner margin. Labial palp with segment I 0.8 × length of segments II and III combined. Segment I ventrally with short, fine, simple setae and one short, stout, simple seta at inner margin. Segment II with thumb-like distomedial protuberance; distomedial protuberance 0.6 × width of base of segment III; ventral surface with short, fine, simple setae; dorsally with a row of seven medium, spine-like, simple setae near outer margin. Segment III conical; apex slightly truncate; length 1.0 × width; ventrally covered with short, spine-like, simple setae and short, fine, simple setae.

***Hind protoptera*** (Fig. 14g) minute.

***Foreleg*** (Fig. 14a, b). Ratio of foreleg segments 1.4:1.0:0.8:0.3. ***Femur*.** Length ca. 3 × maximum width. Dorsal margin with a row of 10–13 curved, spine-like setae in different length, and a partial second row of spine-like setae near margin; length of setae 0.28 × maximum width of femur. Apex rounded, with a pair of curved, spine-like setae and some short, stout, apically rounded setae. Many stout, lanceolate setae scattered along ventral margin; femoral patch absent. ***Tibia*.** Dorsal margin with a row of short, spine-like setae, on apex one longer, spine-like seta. Ventral margin with a row of short to long, curved, spine-like setae, distally of patellotibial suture one long, curved, spine-like seta, on apex some longer, curved, partly bipectinate spine-like setae and a tuft of fine, simple setae. Anterior surface scattered with stout, lanceolate setae. Patellotibial suture present on basal half. ***Tarsus*.** Dorsal margin with a row of short, stout setae. Ventral margin with a row of curved, spine-like setae. Claw with one row of 15–17 denticles; distally pointed; with 5–8 stripes; subapical setae absent.

***Terga*** (Fig. 14c). Surface with irregular rows of U-shaped scale bases and scattered fine, simple setae and micropores. Posterior margin of tergum IV with rounded, partly fused spines, wider than long.

***Gills*** (Fig. 14d). Present on segments II–VII. Margin with small denticles intercalating fine simple setae. Tracheae extending from main trunk to inner and outer margins. Gill IV as long as length of segments V and ½ VI combined. Gill VII as long as length of segments VIII and 1/3 IX combined.

***Paraproct*** (Fig. 14e). Distally not expanded, with ca. 17 stout, marginal spines and some submarginal spines. Surface scattered with U-shaped scale bases, fine, simple setae and micropores. Cercotractor with numerous small, marginal spines.

##### Etymology.

Dedicated to the indigenous Palawano people of Palawan.

##### Distribution.

Philippines: Palawan (incl. Busuanga) (Fig. 48c).

##### Biological aspects.

The specimens were collected at altitudes below 100 m, partly on rock surface in riffles or runs.

##### Type material.

***Holotype*.** Philippines • larva; Palawan, P. Princesa, Bindujan, Talabigan River; 10°01'26"N, 119°04'37"E; 10 m; 27.VII.2019; leg. Freitag and Molls; on slide; GenBank: MT830987; GBIFCH 00763688; PNM. ***Paratypes*.** Philippines • 2 larvae; Palawan, Busuanga, Coron, 4 km E San Nicolas; 12°03'46"N, 120°13'25"E; 25.XII.2019; leg. Freitag; 1 in alcohol; GBIFCH 00515519; ZSM, temporarily stored in AdMU; 1 in alcohol; MT830988; GBIFCH 00763679; PNM • 1 larva; Busuanga, 5 km NW Coron town; Mabintangen R., small mount. riv.; 12°01'45"N, 120°12'19"E; 50 m; 02.II.2020; leg. Freitag; on slide; GBIFCH 00515521; AdMU • 1 larva; Palawan, P. Princesa Cabayugan District, Cabayugan River, near Nagsatayan Creek, S of Martarpi; 10°09'47"N, 118°50'37"E; 37 m; 05.VIII.2000; leg. Freitag; on slide; GBIFCH 00592355; ZSM, temporarily stored in AdMU.

#### Labiobaetis sabordoi sp. nov.

Taxon classificationAnimaliaEphemeropteraBaetidae

9B8A2243-D0CE-59B2-A109-D09200F4FFB4

http://zoobank.org/007986FB-400B-4665-9113-F1A130E70527

**Figure 15.**
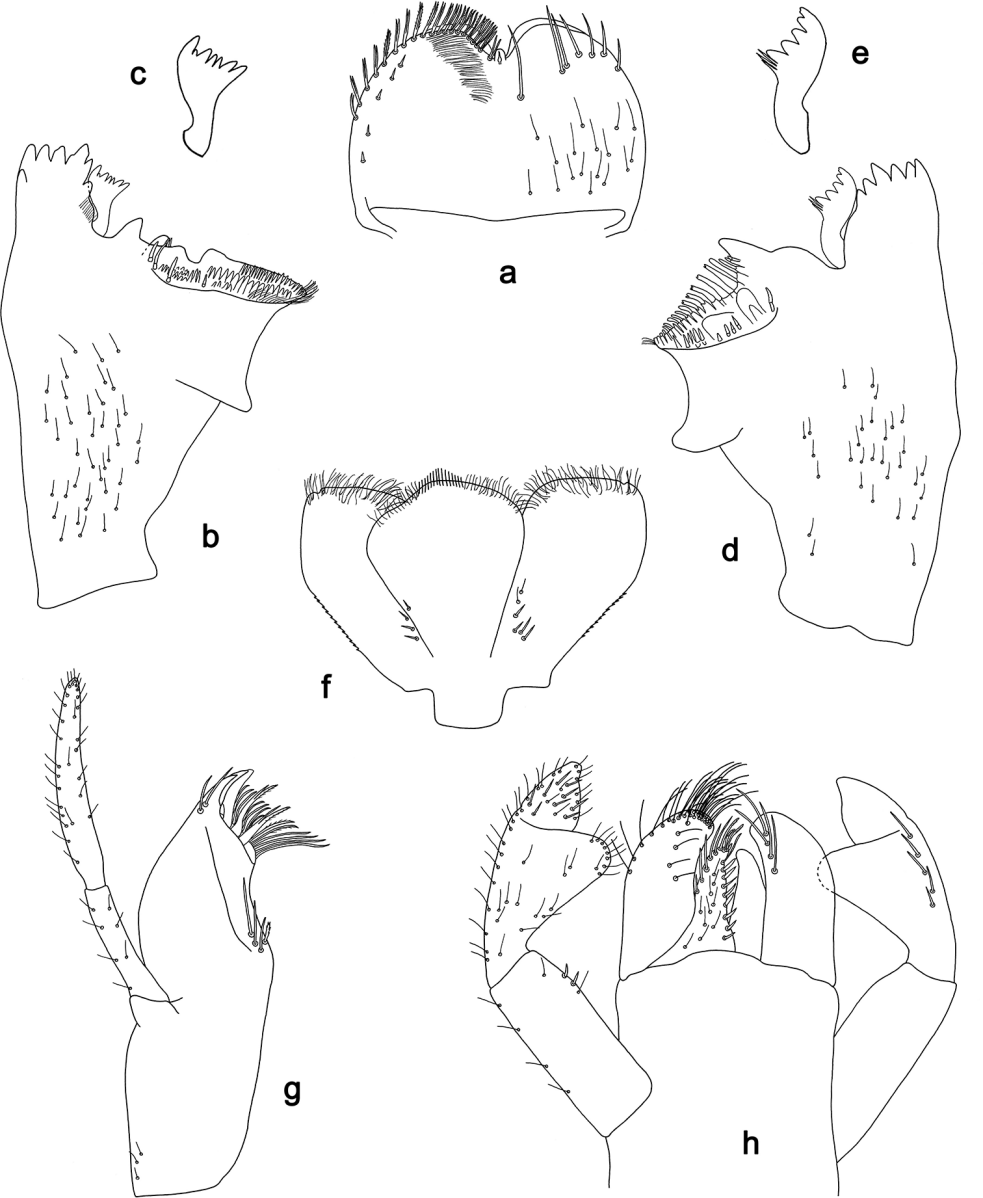
*Labiobaetis sabordoi* sp. nov., larva morphology **a** labrum **b** right mandible **c** right prostheca **d** left mandible **e** left prostheca **f** hypopharynx and superlinguae **g** maxilla **h** labium.

**Figure 16.**
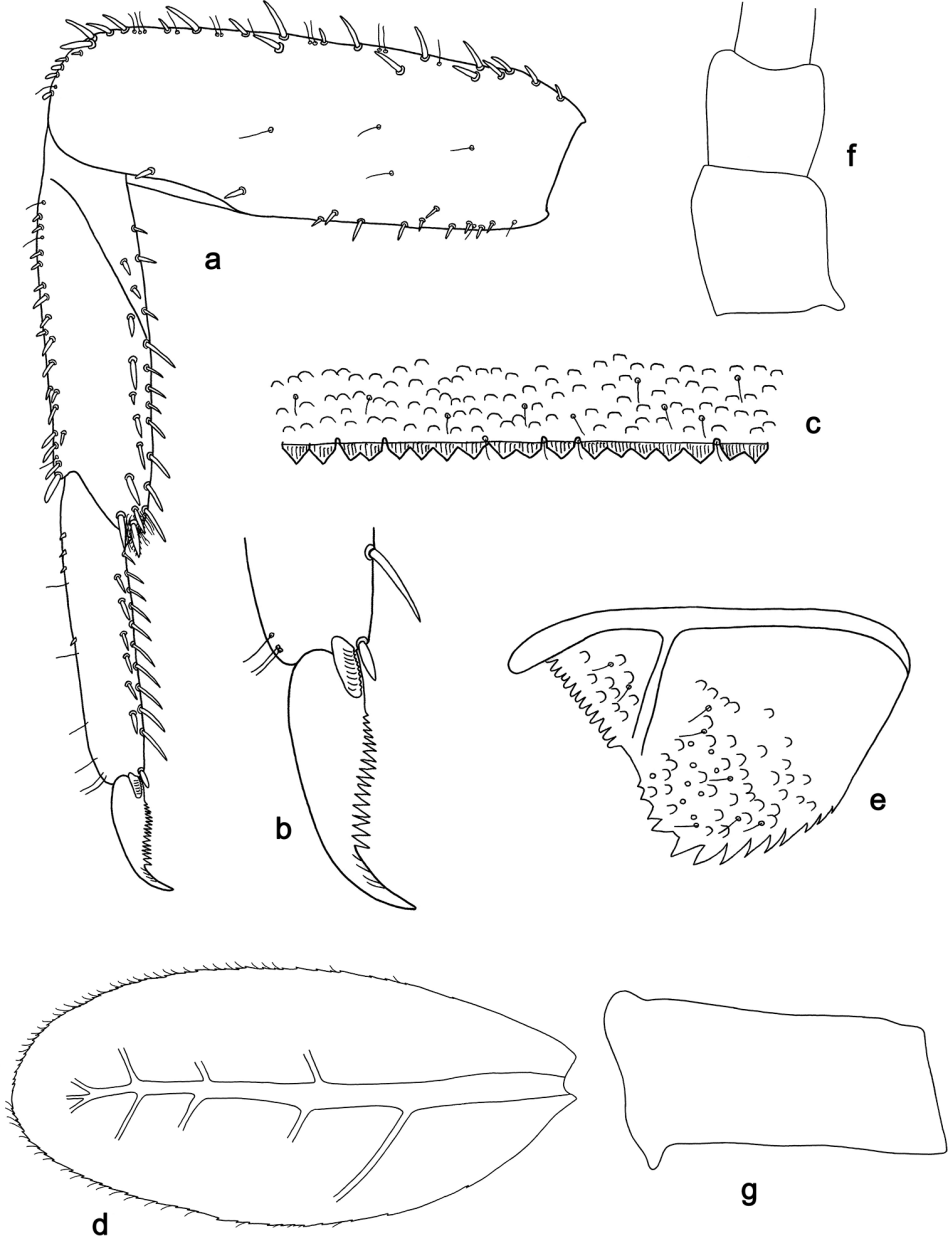
*Labiobaetis sabordoi* sp. nov., larva morphology **a** foreleg **b** fore claw **c** tergum IV **d** gill IV **e** paraproct **f** antennal scape **g** metanotum.

Figures 15 , 16 , 42d , 47c , 48c

##### Diagnosis.

**Larva.** Following combination of characters: A) dorsal surface of labrum with submarginal arc of one plus five or six long, simple setae; B) labial palp segment II with a thumb-like distomedial protuberance, segment III conical; C) mola of right mandible proximally beginning with a double hump; D) hypopharynx with medial tuft of stout setae poorly developed; E) fore femur rather broad, length ca. 3 × maximum width, dorsal margin with 11–14 curved, spine-like setae and a partial second row near margin; F) claw with 16–18 denticles; G) posterior margin of tergum IV with triangular spines, wider than long.

##### Description.

**Larva** (Figs 15, 16, 42d, 47c). Body length 3–3.5 mm. Cerci ca. 2/3 of body length. Paracercus ca. 2/3 of cerci length. Antenna approx. twice as long as head length.

***Colouration*.** Head dorsally light brown with brown markings, thorax and abdomen dorsally brown with bright pattern as in Fig. 42d. Fore protoptera light brown with distinct brown striation. Head, thorax, and abdomen ventrally brown with bright pattern as in Fig. 47c. Legs light brown, femur with two connected brown spots, apically brown. Caudalii light brown, with a brown band at ca. 1/3 of cerci length, distal area of cerci brown.

***Antenna*** (Fig. 16f) with scape and pedicel subcylindrical, without distolateral process at scape.

***Labrum*** (Fig. 15a). Rectangular, length 0.7 × maximum width. Distal margin with medial emargination and a small process. Dorsally with medium, fine, simple setae scattered over surface; submarginal arc of setae composed of one plus five or six long, simple setae, the first two setae after the submedian seta are close together. Ventrally with marginal row of setae composed of lateral and anterolateral long, feathered setae and medial long, bifid, pectinate setae; ventral surface with ca. five short, spine-like setae near lateral and anterolateral margin.

***Right mandible*** (Fig. 15b, c). Incisor and kinetodontium fused. Incisor with four denticles; kinetodontium with three denticles, inner margin of innermost denticle with a row of thin setae. Prostheca robust, apically denticulate. Margin between prostheca and mola with a pronounced hump, mola proximally beginning with a double hump. Tuft of setae at apex of mola present and many thin setae distally at base of mola.

***Left mandible*** (Fig. 15d, e). Incisor and kinetodontium fused. Incisor with three denticles; kinetodontium with three denticles. Prostheca robust, apically with small denticles and comb-shaped structure. Margin between prostheca and mola convex. Subtriangular process long and slender, above level of area between prostheca and mola. Denticles of mola apically constricted and partly with minute secondary dentation at the tips. Tuft of setae at apex of mola present.

Both mandibles with lateral margins almost straight. Basal half with fine, simple setae scattered over dorsal surface.

***Hypopharynx and superlinguae*** (Fig. 15f). Lingua approx. as long as superlinguae. Lingua longer than broad; medial tuft of stout setae poorly developed; distal half laterally expanded. Superlinguae distally straight; lateral margin angulate; fine, long, simple setae along distal margin.

***Maxilla*** (Fig. 15g). Galea-lacinia ventrally with two simple, apical setae under canines. Inner dorsal row of setae with three denti-setae, distal denti-seta tooth-like, middle and proximal denti-setae slender, bifid and pectinate. Medially with one bipectinate, spine-like seta and three or four medium to long, simple setae. Maxillary palp 1.4 × as long as length of galea-lacinia; 2-segmented; palp segment II 1.7 × length of segment I; setae on maxillary palp fine, simple, scattered over surface of segments I and II; apex of last segment slightly pointed, without excavation at inner distolateral margin.

***Labium*** (Fig. 15h). Glossa basally broad, narrowing toward apex; shorter than paraglossa; inner margin with ca. eight short, stout, spine-like setae plus distalmost one much longer, less robust, spine-like seta; apex with two long and one medium, robust, pectinate setae and one short, robust seta; outer margin with five spine-like setae increasing in length distally; ventral surface with fine, simple, scattered setae. Paraglossa sub-rectangular, curved inward; apex rounded; with three rows of long, robust, distally pectinate setae in apical area and four medium, simple setae in anteromedial area; outer margin with some long, spine-like setae; dorsally with a row of four long, spine-like, simple setae near inner margin. Labial palp with segment I 0.9 × length of segments II and III combined. Segment I ventrally with short, fine, simple setae and one or two short, stout, simple setae at inner margin. Segment II with thumb-like distomedial protuberance; distomedial protuberance 0.7 × width of base of segment III; ventral surface with short, fine, simple setae; dorsally with a row of five or six medium, spine-like, simple setae near outer margin. Segment III conical; apex slightly truncate; length 1.2 × width; ventrally covered with short, spine-like, simple setae and short, fine, simple setae.

***Hind protoptera*** (Fig. 16g) minute.

***Foreleg*** (Fig. 16a, b). Ratio of foreleg segments 1.4:1.0:0.8:0.3. ***Femur*.** Length ca. 3 × maximum width. Dorsal margin with a row of 11–14 curved, spine-like setae in different length, proximally and medially a partial second row of spine-like setae near margin; length of setae 0.22 × maximum width of femur. Apex rounded, with a pair of curved, spine-like setae and some short, stout setae. Many stout, lanceolate setae scattered along ventral margin; femoral patch absent. ***Tibia*.** Dorsal margin with a row of short, spine-like setae, on apex one longer, spine-like seta. Ventral margin with a row of short to long, curved, spine-like setae, distally of patellotibial suture one long, curved, spine-like seta, on apex some longer, partly bipectinate, spine-like setae and a tuft of fine, simple setae. Anterior surface scattered with stout, lanceolate setae. Patellotibial suture present on basal half. ***Tarsus*.** Dorsal margin with some short, stout and some fine, simple setae. Ventral margin with a row of curved, spine-like setae and some spine-like setae near margin. Claw with one row of 16–18 denticles; distally pointed; with ca. four stripes; subapical setae absent.

***Terga*** (Fig. 16c). Surface with irregular rows of U-shaped scale bases and scattered fine, simple setae. Posterior margin of tergum IV with triangular, partly fused spines, wider than long, sometimes apically rounded.

***Gills*** (Fig. 16d). Present on segments II–VII. Margin with small denticles intercalating fine simple setae. Tracheae partly extending from main trunk towards outer and inner margins. Gill IV as long as length of segments V and VI combined. Gill VII as long as length of segments VIII and IX combined.

***Paraproct*** (Fig. 16e). Distally not expanded, with 13–23 stout, marginal spines. Surface scattered with U-shaped scale bases, fine, simple setae and micropores. Cercotractor with numerous small, marginal spines.

##### Etymology.

Dedicated to Mr. Marc Ryan Sabordo (Philippines), collector and project assistant of the AdMU Biodiversity Laboratory.

##### Distribution.

Philippines: Negros, Sibuyan and Tablas (Fig. 48c).

##### Biological aspects.

The specimens were collected at altitudes from sea level to 480 m, partly in leaf litter.

##### Type material.

***Holotype*.** Philippines • larva; Negros Oriental, Valencia, Casaroro River downstream; 09°18'N, 123°14'E; 150 m; 01.IX.2019; leg. Garces and Pelingen; on slide; GBIFCH 00592270; PNM. ***Paratypes*.** Philippines • 1 larva; same data as holotype; on slide; GenBank: MT830951; GBIFCH 00654878; ZSM • 2 larvae; Romblon, Tablas, S of San Agustin; 12°33'38"N, 122°07'19"E; 40 m; 14.I.2019; leg. Freitag; 1 on slide; GenBank: MT830952; GBIFCH 00 763674; ZSM; 1 in alcohol; GBIFCH 00515334; ZSM • 2 larvae; Romblon, Sibuyan, Cajidiocan, Cambijang; 12°20'40"N, 122°40'37"E; 5 m; 16.I.2019; leg. Freitag; in alcohol; GBIFCH 00515333; AdMU.

### *Labiobaetis operosus* group of species (Kaltenbach and Gattolliat 2019)

Following combination of characters: A) dorsal surface of labrum with submarginal arc of feathered setae; B) labial palp segment II with thumb-like or lobed distomedial protuberance; C) seven pairs of gills; D) hind protoptera well developed; E) distolateral process at scape well developed; F) fore tarsus with thin setae at ventrodistal margin.

#### Labiobaetis gamay sp. nov.

Taxon classificationAnimaliaEphemeropteraBaetidae

E37055B3-5233-5700-B757-FE3D2CB7D608

http://zoobank.org/8FB0BC17-3C57-4B66-A271-95FA834C5FAC

**Figure 17.**
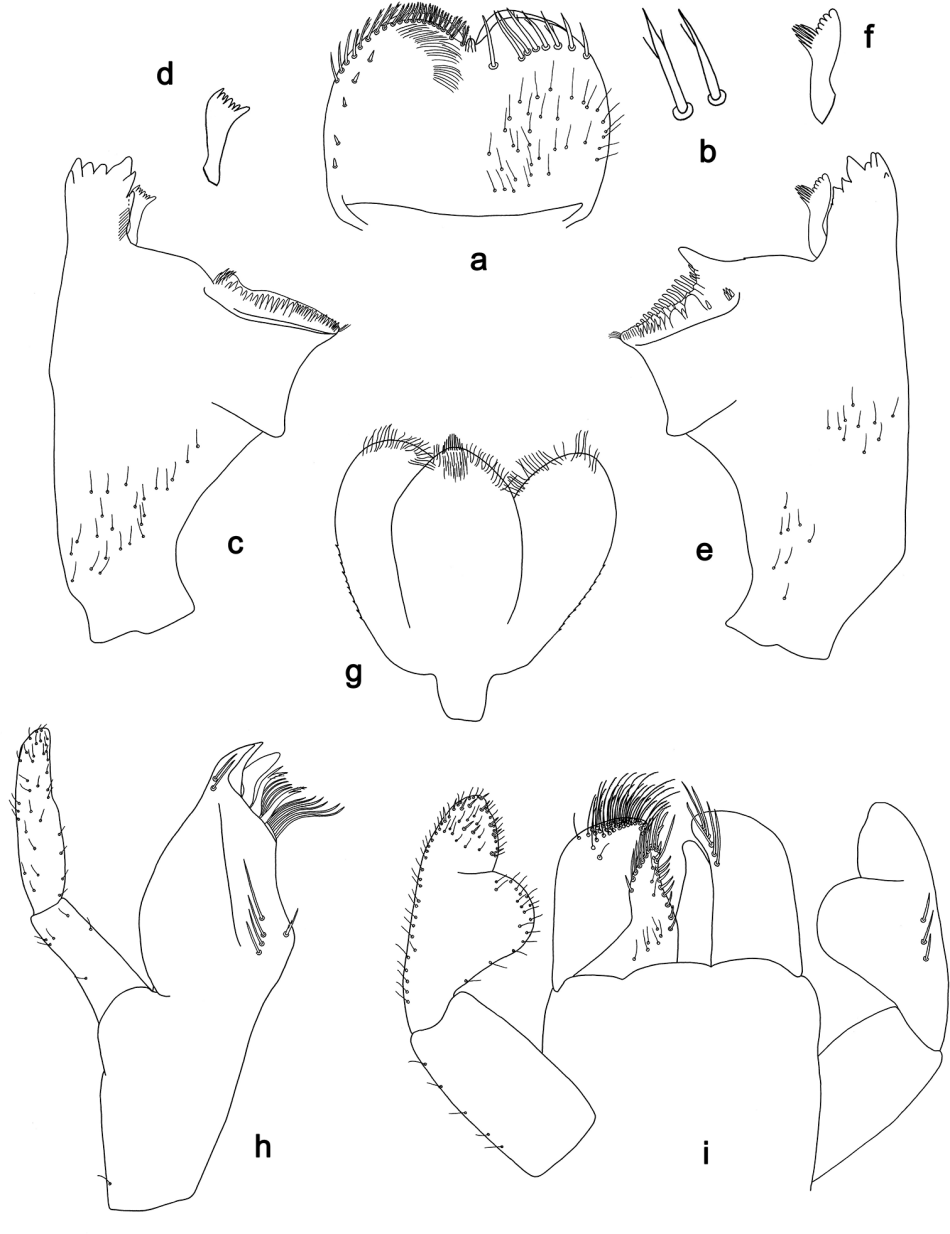
*Labiobaetis gamay* sp. nov., larva morphology **a** labrum **b** setae of arc on dorsal surface of labrum **c** right mandible **d** right prostheca **e** left mandible **f** left prostheca **g** hypopharynx and superlinguae **h** maxilla **i** labium.

**Figure 18.**
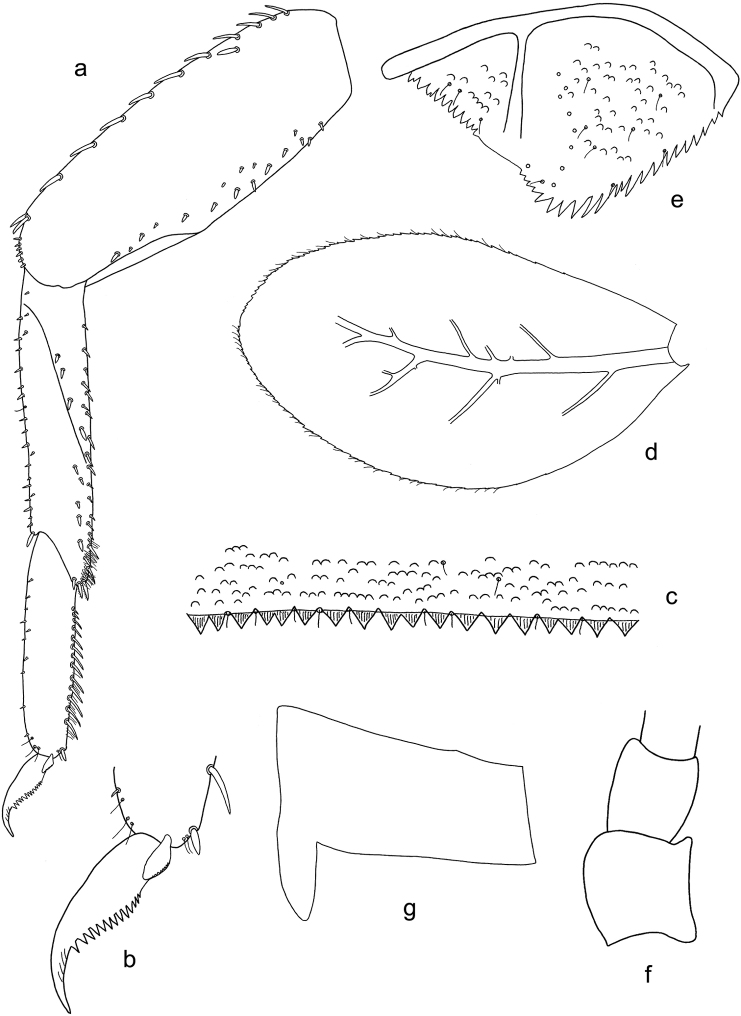
*Labiobaetis gamay* sp. nov., larva morphology **a** foreleg **b** fore claw **c** tergum IV **d** gill IV **e** paraproct **f** antennal scape **g** metanotum.

Figures 17 , 18 , 43a , 49a

##### Diagnosis.

**Larva.** Following combination of characters: A) dorsal surface of labrum with submarginal arc of one plus 7–9 feathered setae with strongly reduced feathers; B) labial palp segment II with a broad, thumb-like distomedial protuberance; segment III conical; C) fore femur rather broad, length ca. 3 × maximum width, dorsal margin with 10–14 curved, spine-like setae; D) hind protoptera well developed; E) paraproct distally not expanded, with 19–22 stout marginal spines.

##### Description.

**Larva** (Figs 17, 18, 43a). Body length 4.6–5.2 mm. Cerci ca. 2/3 of body length. Paracercus ca. 2/3 of cerci length. Antenna approx. twice as long as head length.

***Colouration*.** Head, thorax, and abdomen dorsally grey-brown, with bright pattern as in Fig. 43a, abdominal tergites I, VII, and X mainly light brown. Fore protoptera light brown with darker striation. Head, thorax, and abdomen ventrally ecru, frons and genae grey-brown, abdominal sternites VIII and IX grey-brown. Legs ecru, femur with two sometimes merged grey-brown distomedial spots, tarsus distally grey-brown. Caudalii light brown, with a grey- brown band at ca. 1/3 of cerci, cerci distally grey-brown.

***Antenna*** (Fig. 18f) with scape and pedicel subcylindrical, with well-developed distolateral process at scape.

***Labrum*** (Fig. 17a, b). Rectangular, length 0.7 × maximum width. Distal margin with medial emargination and a small process. Dorsally with medium, fine, simple setae scattered over surface; submarginal arc of setae composed of one plus 7–9 long, feathered setae with strongly reduced feathers. Ventrally with marginal row of setae composed of lateral and anterolateral long, feathered setae and medial long, bifid, pectinate setae; ventral surface with ca. five short, spine-like setae near lateral and anterolateral margin.

***Right mandible*** (Fig. 17c, d). Incisor and kinetodontium fused. Incisor with four denticles; kinetodontium with three denticles, inner margin of innermost denticle with a row of thin setae. Prostheca robust, apically denticulate. Margin between prostheca and mola slightly convex. Tuft of setae at apex of mola present.

***Left mandible*** (Fig. 17e, f). Incisor and kinetodontium fused. Incisor with four denticles; kinetodontium with three denticles. Prostheca robust, apically with small denticles and comb-shaped structure. Margin between prostheca and mola straight, with few minute denticles. Subtriangular process long and slender, above level of area between prostheca and mola. Denticles of mola apically constricted. Tuft of setae at apex of mola present.

Both mandibles with lateral margins almost straight. Basal half with fine, simple setae scattered over dorsal surface.

***Hypopharynx and superlinguae*** (Fig. 17g). Lingua approx. as long as superlinguae. Lingua longer than broad; medial tuft of stout setae well developed, short; distal half laterally expanded. Superlinguae distally rounded; lateral margin rounded; fine, long, simple setae along distal margin.

***Maxilla*** (Fig. 17h). Galea-lacinia ventrally with two simple, apical setae under canines. Inner dorsal row of setae with three denti-setae, distal denti-seta tooth-like, middle and proximal denti-setae slender, bifid and pectinate. Medially with one bipectinate, spine-like seta and four long, simple setae. Maxillary palp slightly longer than length of galea-lacinia; 2-segmented; palp segment II 1.4 × length of segment I; setae on maxillary palp fine, simple, scattered over surface of segments I and II; apex of last segment rounded, with slight excavation at inner distolateral margin.

***Labium*** (Fig. 17i). Glossa basally broad, narrowing toward apex; shorter than paraglossa; inner margin with ca. nine spine-like setae, distalmost seta much longer than other setae; apex with two long and one medium, robust, pectinate setae and one short, robust seta; outer margin with seven spine-like setae increasing in length distally; ventral surface with fine, simple, scattered setae. Paraglossa sub-rectangular, curved inward; apex rounded; with three rows of long, robust, distally pectinate setae in apical area and one or two medium, simple setae in anteromedial area; dorsally with a row of five long, spine-like, simple setae near inner margin. Labial palp with segment I 0.8 × length of segments II and III combined. Segment I ventrally with short, fine, simple setae. Segment II with broad thumb-like distomedial protuberance; distomedial protuberance 0.7 × width of base of segment III; ventral surface with short, fine, simple setae; dorsally with a row of three long, spine-like setae near outer margin. Segment III conical; apex slightly truncate; length 1.1 × width; ventrally covered with short, spine-like, simple setae and short, fine, simple setae.

***Hind protoptera*** (Fig. 18g) well developed.

***Foreleg*** (Fig. 18a, b). Ratio of foreleg segments 1.4:1.0:0.8:0.3. ***Femur*.** Length ca. 3 × maximum width. Dorsal margin with 10–14 curved, spine-like setae, mostly one or two setae additionally near margin in basal area; length of setae 0.19 × maximum width of femur. Apex rounded, with a pair of spine-like setae and some short, stout setae. Many stout, lanceolate setae scattered along ventral margin; femoral patch absent. ***Tibia*.** Dorsal margin with a row of short, spine-like setae and fine simple setae, on apex one longer, spine-like seta. Ventral margin with a row of short, curved, spine-like setae, on apex some longer, partly bipectinate setae and a tuft of fine, simple setae. Anterior surface scattered with stout, lanceolate setae. Patellotibial suture present on basal 2/3. ***Tarsus*.** Dorsal margin with a row of short, stout setae and fine, simple setae. Ventral margin with a row of curved, spine-like setae and in distal area fine simple setae. Claw with one row of 12 or 13 denticles; distally pointed; with ca. four stripes; subapical setae absent.

***Terga*** (Fig. 18c). Surface with irregular rows of U-shaped scale bases and scattered fine, simple setae. Posterior margin of tergum IV with triangular spines, wider than long.

***Gills*** (Fig. 18d). Present on segments I–VII. Margin with small denticles intercalating fine simple setae. Tracheae extending from main trunk to inner and outer margins. Gill I ca. 2/3 length of segment II. Gill IV as long as length of segments V and ½ VI combined. Gill VII as long as length of segment VIII.

***Paraproct*** (Fig. 18e). Distally not expanded, with 19–22 stout, marginal spines. Surface scattered with U-shaped scale bases and fine, simple setae. Cercotractor with numerous small, marginal spines.

##### Etymology.

Named after the Cebuano word meaning reduced, referring to the strongly reduced feathers of the submarginal setae on the dorsal labrum surface.

##### Distribution.

Philippines: Luzon and Mindoro (Fig. 49a).

##### Biological aspects.

The specimens were collected at altitudes from sea level to 140 m, partly on hygropetric rocks or rock surface in riffles or runs.

##### Type material.

***Holotype*.** Philippines • larva; Oriental Mindoro, Roxas, Brgy. San Vicente, lower reach of Taugad River; 12°37.30'N, 121°22.97'E; 140 m; 2016–2019; leg. Freitag and Garces; on slide; GBIFCH 00592274; PNM. ***Paratypes*.** Philippines • 27 larvae; same data as holotype; 2 on slide; GenBank. MT830953, MT830954; GBIFCH 00 654922, GBIFCH 00763637; ZSM; 25 in alcohol; GBIFCH 00515433, GBIFCH 00515434, GBIFCH 00515458, GBIFCH 00515460; ZSM • 10 larvae; Oriental Mindoro, Roxas, Brgy. San Vicente; 12°37'06"N, 121°23'49"E; 140 m; 2016–2019; leg. Freitag, Garces and Pangantihon; 2 on slides; GenBank: MT830955; GBIFCH 00763639, GBIFCH 00763338; AdMU; 8 in alcohol; GBIFCH 00515435, GBIFCH 00515459; AdMU • 15 larvae; Luzon, La Union, Sudipen Municipality, Amburayan River; 16°54'38"N, 120°28'40"E; 20 m; 14.IV.2019; leg. Freitag, Garces and Pangantihon; 1 on slide; GenBank: MT830956; GBIFCH 00763655; ZSM; 14 in alcohol; GBIFCH 00515436, GBIFCH 00515461; ZSM • 31 larvae; Luzon, Ilocos Sur, Suyo Municipality, big river downstream Sangbay n. Ragsak; 16°59'32"N, 120°32'21"E; 100 m; 15.IV.2019; leg. Freitag, Garces and Pangantihon; 1 on slide; GenBank: MT830957; GBIFCH 00763657; ZSM; 30 in alcohol; GBIFCH 00515437; ZSM • 14 larvae; Luzon, La Union, San Juan Municipality, Baroro River; 16°39'27"N, 120°25'55"E; 90 m; 19.IV.1996; leg. Mendoza; 1 on slide; GenBank: MT830958; GBIFCH 00763658; ZSM; 13 in alcohol; GBIFCH 00515438, GBIFCH 515439; ZSM.

#### Labiobaetis pangantihoni sp. nov.

Taxon classificationAnimaliaEphemeropteraBaetidae

45859324-A12F-529F-B9AB-75DE737FE918

http://zoobank.org/AAA2FE3C-DA89-41AA-A20A-4AF457BB0848

**Figure 19.**
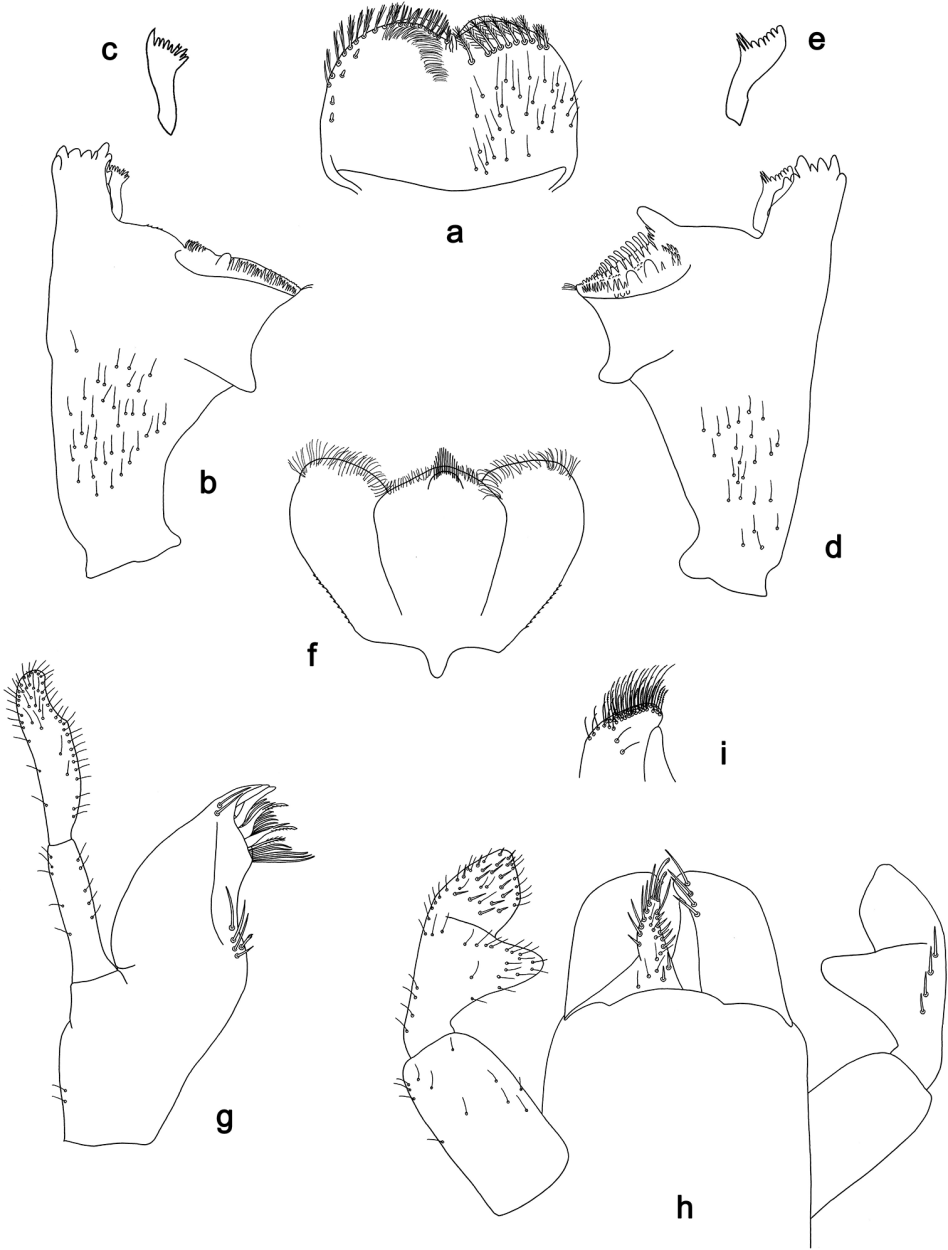
*Labiobaetis pangantihoni* sp. nov., larva morphology **a** labrum **b** right mandible **c** right prostheca **d** left mandible **e** left prostheca **f** hypopharynx and superlinguae **g** maxilla **h** labium **i** apex of paraglossa.

**Figure 20.**
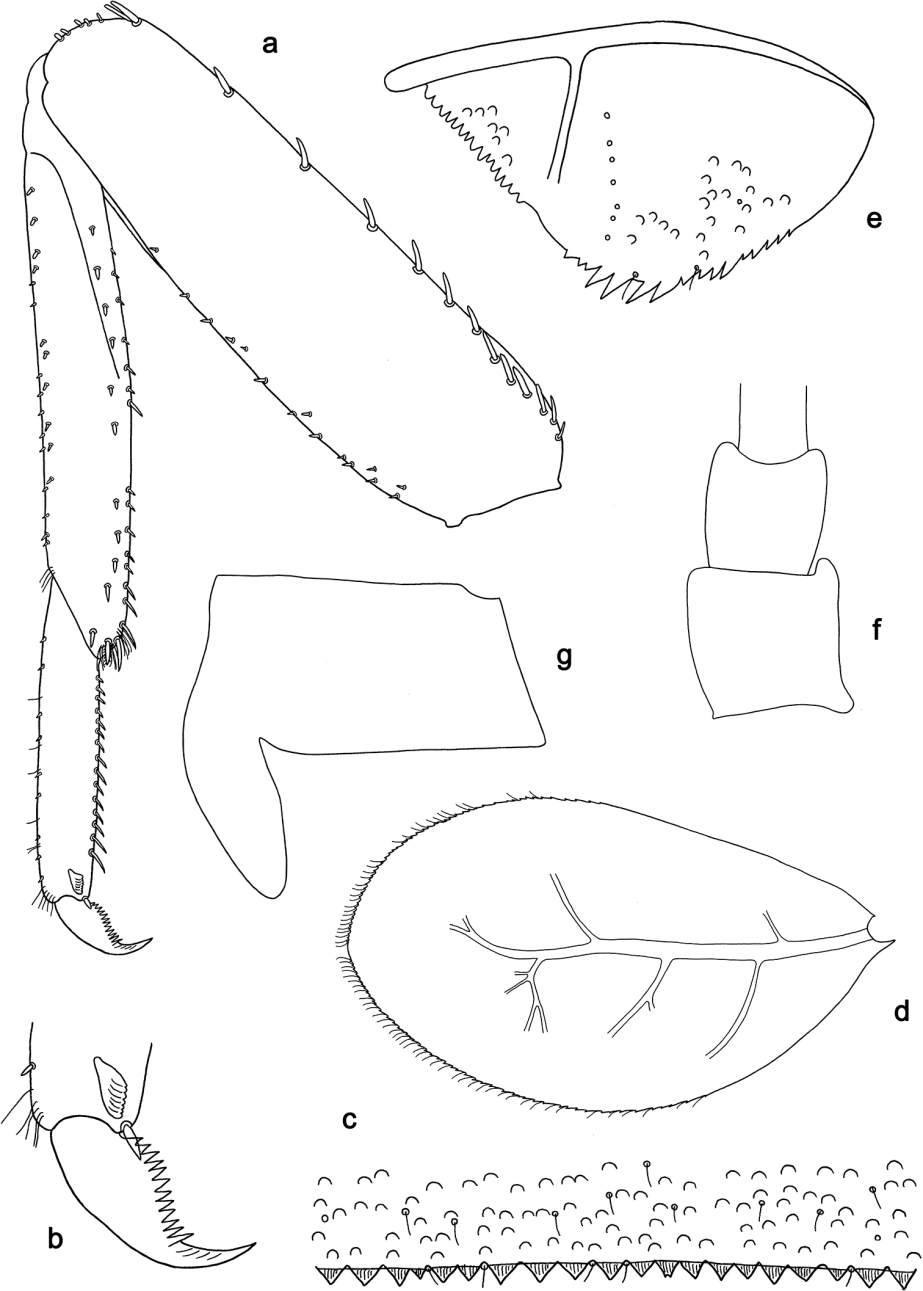
*Labiobaetis pangantihoni* sp. nov., larva morphology **a** foreleg **b** fore claw **c** tergum IV **d** gill IV **e** paraproct **f** antennal scape **g** metanotum.

Figures 19 , 20 , 43b , 49a

##### Diagnosis.

**Larva.** Following combination of characters: A) dorsal surface of labrum with submarginal arc of one plus nine or ten long, feathered setae; B) labial palp segment II with a rather slender, thumb-like distomedial protuberance, segment III slightly pentagonal; C) fore femur rather broad, length 3.4 × maximum width, dorsal margin with eleven or 12 curved, spine-like setae; D) claw with 10–13 denticles; E) paraproct distally not expanded, with ca. 16 stout marginal spines.

##### Description.

**Larva** (Figs 19, 20, 43b). Body length 3.5–4 mm. Cerci ca. 2/3 of body length. Paracercus ca. 2/3 of cerci length. Antenna approx. 2.5 × as long as head length.

***Colouration*.** Head, thorax, and abdomen dorsally brown, with bright pattern as in Fig. 43b, fore protoptera brown with bright striation. Head, thorax, and abdomen ventrally light brown, genae brown, abdominal segments VIII and IX darker brown. Legs light brown, femur with distomedial brown spot, apex brown, tibia distally brown. Caudalii light brown with a brown band at ca. 1/3 of cerci length.

***Antenna*** (Fig. 20f) with scape and pedicel subcylindrical, with well-developed distolateral process at scape.

***Labrum*** (Fig. 19a). Rectangular, length 0.7 × maximum width. Distal margin with medial emargination and a small process. Dorsally with medium, fine, simple setae scattered over surface; submarginal arc of setae composed of one plus nine or ten long, feathered setae. Ventrally with marginal row of setae composed of lateral and anterolateral long, feathered setae and medial long, bifid setae; ventral surface with ca. four short, spine-like setae near lateral and anterolateral margin.

***Right mandible*** (Fig. 19b, c). Incisor and kinetodontium fused. Incisor with five denticles; kinetodontium with three denticles, inner margin of innermost denticle without a row of thin setae. Prostheca robust, apically denticulate. Margin between prostheca and mola slightly convex, with minute denticles. Tuft of setae at apex of mola present.

***Left mandible*** (Fig. 19d, e). Incisor and kinetodontium fused. Incisor with four denticles; kinetodontium with three denticles. Prostheca robust, apically with small denticles and comb-shaped structure. Margin between prostheca and mola straight. Subtriangular process long and slender, above level of area between prostheca and mola. Denticles of mola apically constricted. Tuft of setae at apex of mola present.

Both mandibles with lateral margins almost straight. Basal half with fine, simple setae scattered over dorsal surface.

***Hypopharynx and superlinguae*** (Fig. 19f). Lingua approx. as long as superlinguae. Lingua longer than broad; medial tuft of stout setae well developed, short; distal half laterally expanded. Superlinguae distally rounded; lateral margin rounded; fine, long, simple setae along distal margin.

***Maxilla*** (Fig. 19g). Galea-lacinia ventrally with two simple, apical setae under canines. Inner dorsal row of setae with three denti-setae, distal denti-seta tooth-like, middle and proximal denti-setae slender, bifid and pectinate. Medially with one bipectinate, spine-like seta and four or five long, simple setae. Maxillary palp 1.3 × as long as length of galea-lacinia; 2-segmented; palp segment II 1.3 × length of segment I; setae on maxillary palp fine, simple, scattered over surface of segments I and II; apex of last segment rounded, with excavation at inner distolateral margin.

***Labium*** (Fig. 19h, i). Glossa basally broad, narrowing toward apex; shorter than paraglossa; inner margin with ca. eight spine-like setae, distalmost seta much longer than other setae; apex with two long and one medium, robust, pectinate setae; outer margin with six medium to long, spine-like setae; ventral surface with short, fine, simple, scattered setae. Paraglossa sub-rectangular, curved inward; apex rounded; with three rows of long, robust, distally pectinate setae in apical area and two medium, simple setae in anteromedial area; dorsally with a row of four long, spine-like, simple setae near inner margin. Labial palp with segment I 0.9 × length of segments II and III combined. Segment I ventrally with short, fine, simple setae. Segment II with rather slender, thumb-like distomedial protuberance; distomedial protuberance 0.8 × width of base of segment III; ventral surface with short, fine, simple setae; dorsally with a row of four spine-like, simple setae near outer margin. Segment III slightly pentagonal; apex slightly truncate; length 1.1 × width; ventrally covered with short, spine-like, simple setae and short, fine, simple setae.

***Hind protoptera*** (Fig. 20g) well developed.

***Foreleg*** (Fig. 20a, b). Ratio of foreleg segments 1.2:1.0:0.6:0.2. ***Femur*.** Length ca. 3 × maximum width. Dorsal margin with eleven or twelve curved, spine-like setae; length of setae 0.16 × maximum width of femur. Apex rounded, with a pair of spine-like setae and some short, stout setae. Many stout, lanceolate setae scattered along ventral margin; femoral patch absent. ***Tibia*.** Dorsal margin with a row of short, spine-like setae, near margin another row of short, spine-like setae. Ventral margin with row of short to medium, curved, spine-like setae, on apex some longer and partly bipectinate setae and a tuft of fine, simple setae. Anterior surface scattered with stout, lanceolate setae. Patellotibial suture present on basal half. ***Tarsus*.** Dorsal margin with a row of short stout setae and fine, simple setae. Ventral margin with a row of curved, spine-like setae. Claw with one row of 10–13 denticles; distally pointed; with ca. four stripes; subapical setae absent.

***Terga*** (Fig. 20c). Surface with irregular rows of U-shaped scale bases and scattered fine, simple setae. Posterior margin of tergum IV with triangular spines, wider than long.

***Gills*** (Fig. 20d). Present on segments I–VII. Margin with small denticles intercalating fine simple setae. Tracheae extending from main trunk to inner and outer margins. Gill I ca. 1/2 length of segment II. Gill IV as long as length of segments V and 1/2 VI combined. Gill VII as long as length of segments VIII and 1/4 IX combined.

***Paraproct*** (Fig. 20e). Distally not expanded, with ca. 16 stout, marginal spines. Surface scattered with U-shaped scale bases, fine, simple setae and micropores. Cercotractor with numerous small, marginal spines.

##### Etymology.

Dedicated to Mr. Clister V. Pangantihon, outstanding collector, entomologist and project assistant of the AdMU Biodiversity Laboratory.

##### Distribution.

Philippines: Palawan (Fig. 49a).

##### Biological aspects.

The specimens were collected at altitudes from sea level to 180 m, partly on submerged wood in runs or riffles.

##### Type material.

***Holotype*.** Philippines • larva; Palawan, Balabac, Danglis River near the road; 07°57'39"N, 117°02'59"E; 170 m; 2019; leg. Pelingen and Pangantihon; on slide; GBIFCH 00592336; PNM. ***Paratypes*.** Philippines • 9 larvae; same data as holotype; 3 on slides; GenBank: MT830959; GBIFCH 00592321, GBIFCH 00763684, GBIFCH 00592335; ZSM, temporarily stored in AdMU; 5 in alcohol; GBIFCH 00515426; PCSD; 1 in alcohol; GBIFCH 00515400; AdMU • 1 larva; Palawan, Balabac, Danglis Falls/Busay; 07°57'39"N, 117°02'59"E; 180 m; 2019; leg. Pelingen and Pangantihon; in alcohol; GBIFCH 00515427; AdMU • 5 larvae; Palawan, Quezon, Isugod, Aramaywan River; 09°21'07"N, 118°08'26"E; 14 m; 2019; leg. Pangantihon and Pelingen; 1 on slide; GBIFCH 00592331; AdMU; 2 in alcohol; GBIFCH 00515429; PNM; 2 in alcohol; GBIFCH 00515401, GBIFCH 00515402; AdMU • 2 larvae; Palawan, Quezon, Aramaywan River; 09°18'25"N, 118°07'42"E; 20 m; 2019; leg. Pangantihon and Pelingen; in alcohol; GBIFCH 00515428; AdMU.

#### Labiobaetis tagbanwa sp. nov.

Taxon classificationAnimaliaEphemeropteraBaetidae

F8636E7D-4762-5C21-B77E-7D82505643E5

http://zoobank.org/F6926F19-3CEB-4C21-8E62-A14C0D7F4839

**Figure 21.**
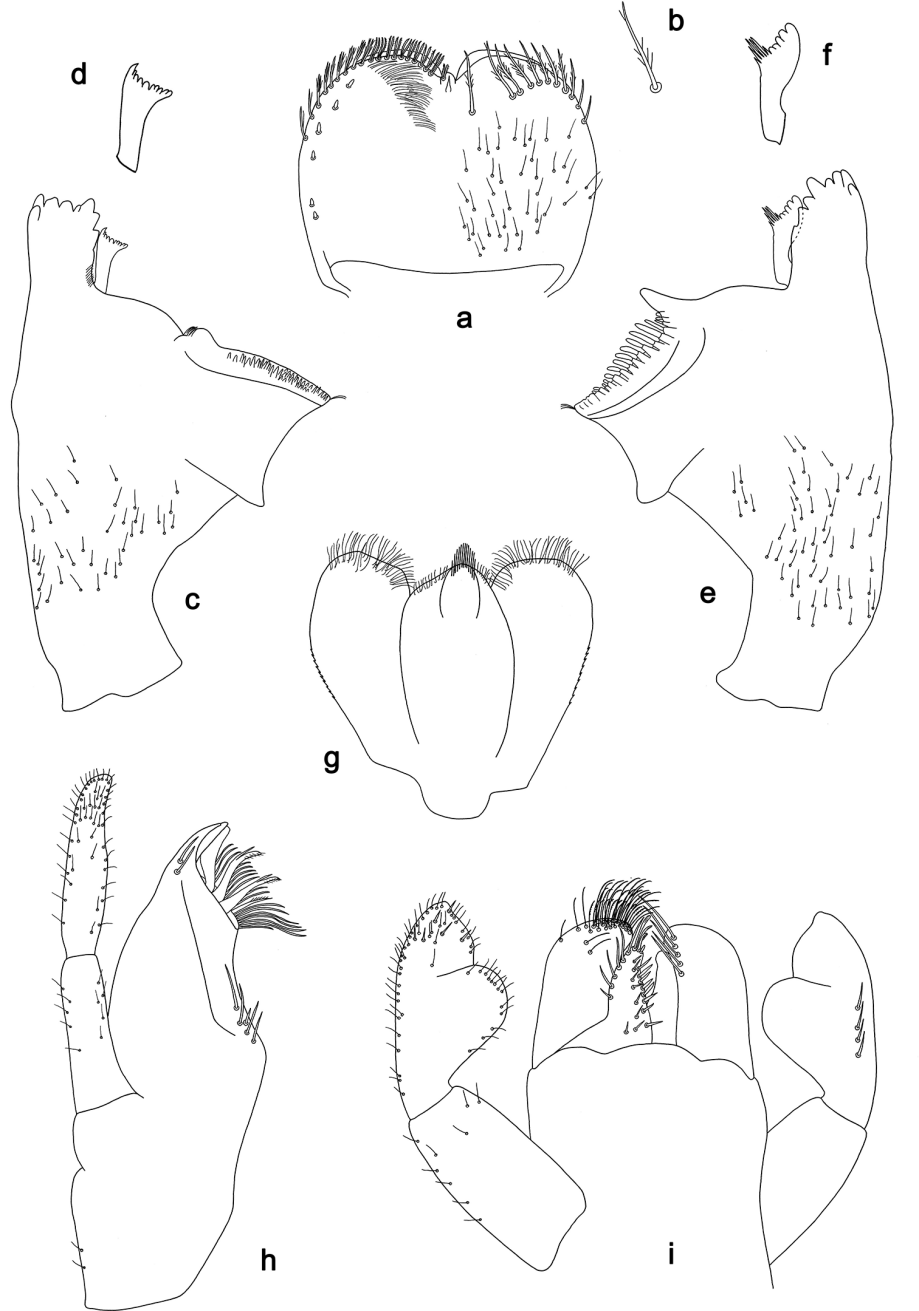
*Labiobaetis tagbanwa* sp. nov., larva morphology **a** labrum **b** seta of arc on dorsal surface of labrum **c** right mandible **d** right prostheca **e** left mandible **f** left prostheca **g** hypopharynx and superlinguae **h** maxilla **i** labium.

**Figure 22.**
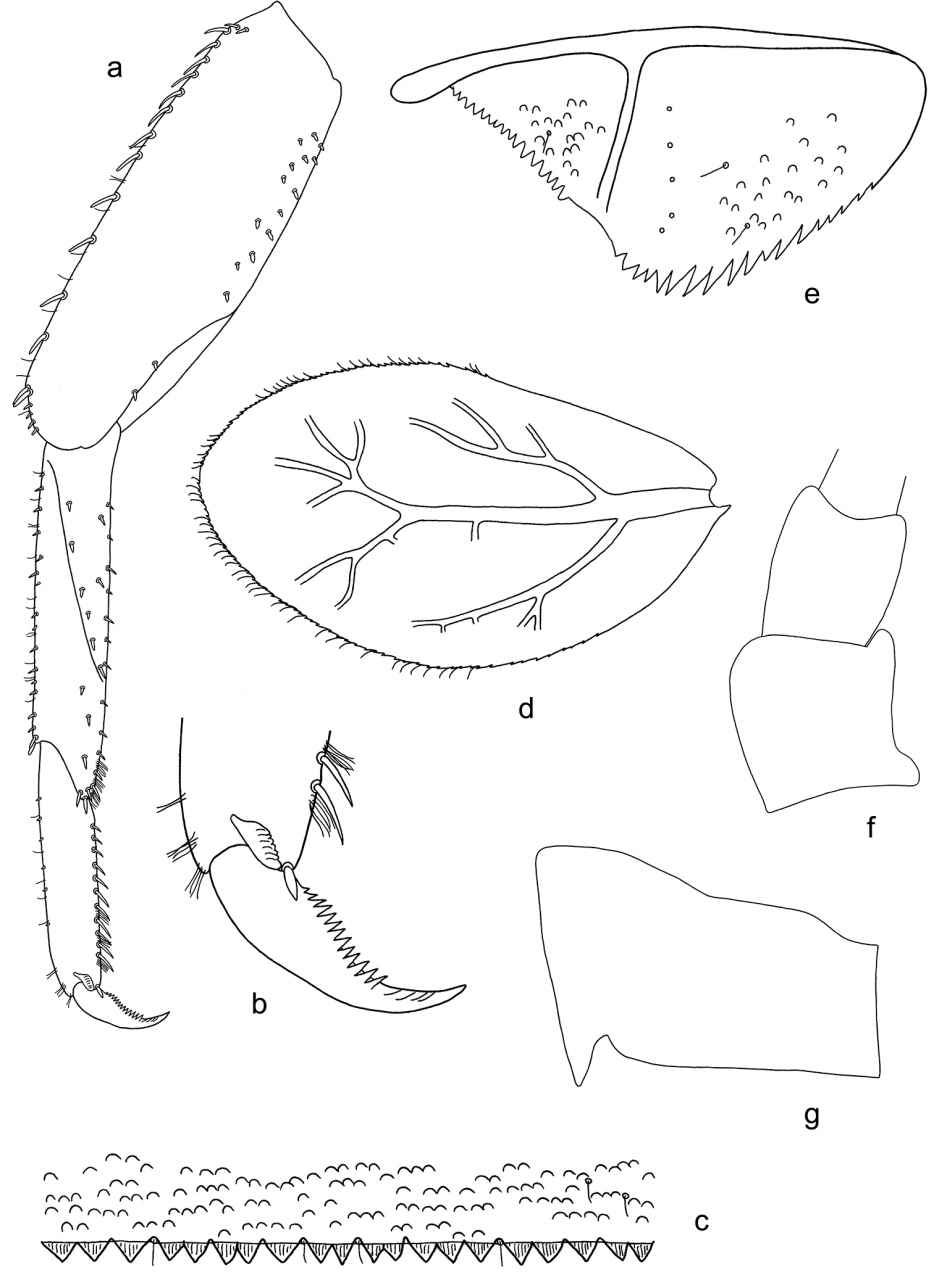
*Labiobaetis tagbanwa* sp. nov., larva morphology **a** foreleg **b** fore claw **c** tergum IV **d** gill IV **e** paraproct **f** antennal scape **g** metanotum.

Figures 21 , 22 , 43c , 49a

##### Diagnosis.

**Larva.** Following combination of characters: A) dorsal surface of labrum with submarginal arc of one plus 8–10 long, feathered setae with reduced feathers; B) labial palp segment II with a broad thumb-like distomedial protuberance, segment III conical; C) fore femur rather broad, length ca. 3 × maximum width, dorsal margin with ca. 13 curved, spine-like setae; D) hind protoptera medium developed; E) paraproct distally not expanded, with ca. 18 stout, marginal spines.

##### Description.

**Larva** (Figs 21, 22, 43c). Body length 4.1–5.3 mm. Cerci ca. 3/4 of body length. Paracercus ca. 2/3 of cerci length. Antenna approx. twice as long as head length.

***Colouration*.**

Head, thorax, and abdomen dorsally brown, with bright markings as in Fig. 43c, abdominal segments I, VII and X light brown, fore protoptera basally brown, distally light brown with bright striation. Head, thorax, and abdomen ventrally light brown, genae brown, abdominal segments laterally somewhat darker, abdominal segments VIII and IX brown, abdominal segment V, VI and VII medially with darker areas. Legs ecru, femur with distomedial brown spot, apex brown, tibia and tarsus distally light brown. Caudalii light brown, with a brown band at ca. 1/3 of cerci length, cerci distally brown.

***Antenna*** (Fig. 22f) with scape and pedicel subcylindrical, with well-developed distolateral process at scape.

***Labrum*** (Fig. 21a, b). Rectangular, length 0.8 × maximum width. Distal margin with medial emargination and a small process. Dorsally with medium, fine, simple setae scattered over surface; submarginal arc of setae composed of one plus 8–10 long, feathered setae with reduced feathers. Ventrally with marginal row of setae composed of lateral and anterolateral long, feathered setae and medial long, bifid, pectinate setae; ventral surface with ca. six short, spine-like setae near lateral and anterolateral margin.

***Right mandible*** (Fig. 21c, d). Incisor and kinetodontium fused. Incisor with five denticles; kinetodontium with three denticles, inner margin of innermost denticle with a row of thin setae. Prostheca robust, apically denticulate. Margin between prostheca and mola slightly convex. Tuft of setae at apex of mola present.

***Left mandible*** (Fig. 21e, f). Incisor and kinetodontium fused. Incisor with four denticles; kinetodontium with three denticles. Prostheca robust, apically with small denticles and comb-shaped structure. Margin between prostheca and mola slightly convex. Subtriangular process long and slender, above level of area between prostheca and mola. Denticles of mola apically constricted. Tuft of setae at apex of mola present.

Both mandibles with lateral margins almost straight. Basal half with fine, simple setae scattered over dorsal surface.

***Hypopharynx and superlinguae*** (Fig. 21g). Lingua approx. as long as superlinguae. Lingua longer than broad; medial tuft of stout setae well developed, short; distal half not expanded. Superlinguae distally rounded; lateral margin rounded; fine, long, simple setae along distal margin.

***Maxilla*** (Fig. 21h). Galea-lacinia ventrally with two simple, apical setae under canines. Inner dorsal row of setae with three denti-setae, distal denti-seta tooth-like, middle and proximal denti-setae slender, bifid and pectinate. Medially with one bipectinate, spine-like seta and four long, simple setae. Maxillary palp 1.2 × as long as length of galea-lacinia; 2-segmented; palp segment II 1.2 × length of segment I; setae on maxillary palp fine, simple, scattered over surface of segments I and II; apex of last segment rounded, with slight excavation at inner distolateral margin.

***Labium*** (Fig. 21i). Glossa basally broad, narrowing toward apex; shorter than paraglossa; inner margin with ca. eight spine-like setae; apex with two long and one medium, robust, pectinate setae and one short, robust seta; outer margin with six medium to long spine-like setae; ventral surface with short, simple setae. Paraglossa sub-rectangular, curved inward; apex rounded; with three rows of long, robust, distally pectinate setae in apical area and two medium, simple setae in anteromedial area; dorsally with a row of five long, spine-like, simple setae near inner margin. Labial palp with segment I 0.9 × length of segments II and III combined. Segment I ventrally with short, fine, simple setae. Segment II with broad thumb-like distomedial protuberance; distomedial protuberance 0.5 × width of base of segment III; ventral surface with short, fine, simple setae; dorsally with a row of three or four spine-like, simple setae near outer margin. Segment III conical; apex slightly pointed; length 0.9 × width; ventrally covered with short, spine-like, simple setae and short, fine, simple setae.

***Hind protoptera*** (Fig. 22g) medium developed.

***Foreleg*** (Fig. 22a, b). Ratio of foreleg segments 1.5:1.0:0.7:0.3. ***Femur*.** Length ca. 3 × maximum width. Dorsal margin with ca. 13 curved, spine-like setae; length of setae 0.18 × maximum width of femur. Apex rounded, with a pair of spine-like setae and some short, stout setae. Many stout, lanceolate setae scattered along ventral margin; femoral patch absent. ***Tibia*.** Dorsal margin with a row of short, spine-like setae and fine, simple setae, on apex a pair of longer, spine-like setae. Ventral margin with a row of short, curved, spine-like setae, on apex some longer setae and a tuft of fine, simple setae. Anterior surface scattered with stout, lanceolate setae. Patellotibial suture present on basal 2/3. ***Tarsus*.** Dorsal margin with a row of short, stout setae and fine, simple setae. Ventral margin with a row of curved, spine-like setae and in distal area fine simple setae. Claw with one row of 11–14 denticles; distally pointed; with ca. four stripes; subapical setae absent.

***Terga*** (Fig. 22c). Surface with irregular rows of U-shaped scale bases and scattered fine, simple setae. Posterior margin of tergum IV with triangular spines, wider than long.

***Gills*** (Fig. 22d). Present on segments I–VII. Margin with small denticles intercalating fine simple setae. Tracheae extending from main trunk to inner and outer margins. Gill I ca. 1/2 length of segment II. Gill IV as long as length of segments V and 1/2 VI combined. Gill VII as long as length of segment VIII.

***Paraproct*** (Fig. 22e). Distally not expanded, with ca. 18 stout, marginal spines. Surface scattered with U-shaped scale bases and fine, simple setae. Cercotractor with numerous small, marginal spines.

##### Etymology.

Dedicated to the indigenous Tagbanwa people of Palawan.

##### Distribution.

Philippines: Palawan (Fig. 49a).

##### Biological aspects.

The specimens were collected from sea level to 100 m on rock surface or submerged wood in riffles or runs.

##### Type material.

***Holotype*.** Philippines • larva; Palawan, Quezon, Aramaywan River, cogon grass; 09°22'33"N, 118°08'41"E; 15 m; 2019; leg. Pangantihon and Pelingen; on slide; GBIFCH 00592324; PNM. ***Paratypes*.** Philippines • 16 larvae; same data as holotype; 1 on slide; GenBank: MT830962; GBIFCH 00763680; ZSM, temporarily stored in AdMU; 1 on slide; GBIFCH 00592350; AdMU; 3 in alcohol; GBIFCH 00515432, GBIFCH 00515457; PNM; 2 in alcohol; GenBank: MT830961; GBIFCH 00763681, GBIFCH 00515430; ZSM, temporarily stored in AdMU; 6 in alcohol; GBIFCH 00515431; PCSD; 3 in alcohol; GBIFCH 00515410; AdMU • 1 larva; Palawan, Puerto Princesa, Luzviminda, Iwahig River; 09°41'20"N 118°37'29"E; 13.VI.2009; leg. Freitag; on slide; GenBank: MT830960; GBIFCH 00654885; AdMU • 1 larva; Palawan, Aborlan, Cabigaan, Talakaigan, mount. Riv. upstr. dam; 09°26'55"N, 118°26'44"E; 100 m; 14.VI.1995; leg. Freitag; in alcohol; GBIFCH 00515456; AdMU.

#### Labiobaetis valdezorum sp. nov.

Taxon classificationAnimaliaEphemeropteraBaetidae

E3287CF3-0A0E-57CD-B089-806C288947E2

http://zoobank.org/DCC0D1B4-B4C0-4FF4-A224-173008DCAF39

**Figure 23.**
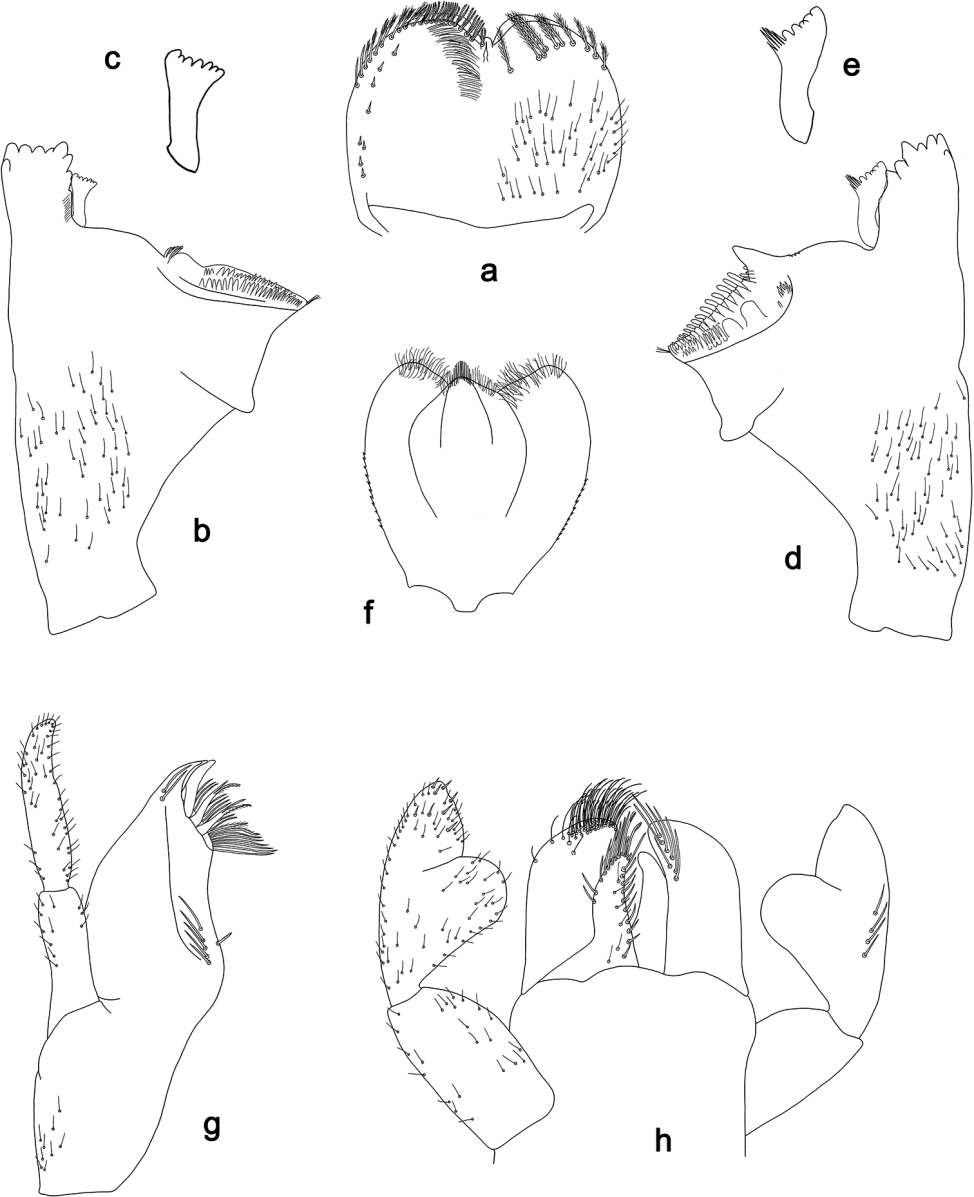
*Labiobaetis valdezorum* sp. nov., larva morphology **a** labrum **b** right mandible **c** right prostheca **d** left mandible **e** left prostheca **f** hypopharynx and superlinguae **g** maxilla **h** labium.

**Figure 24.**
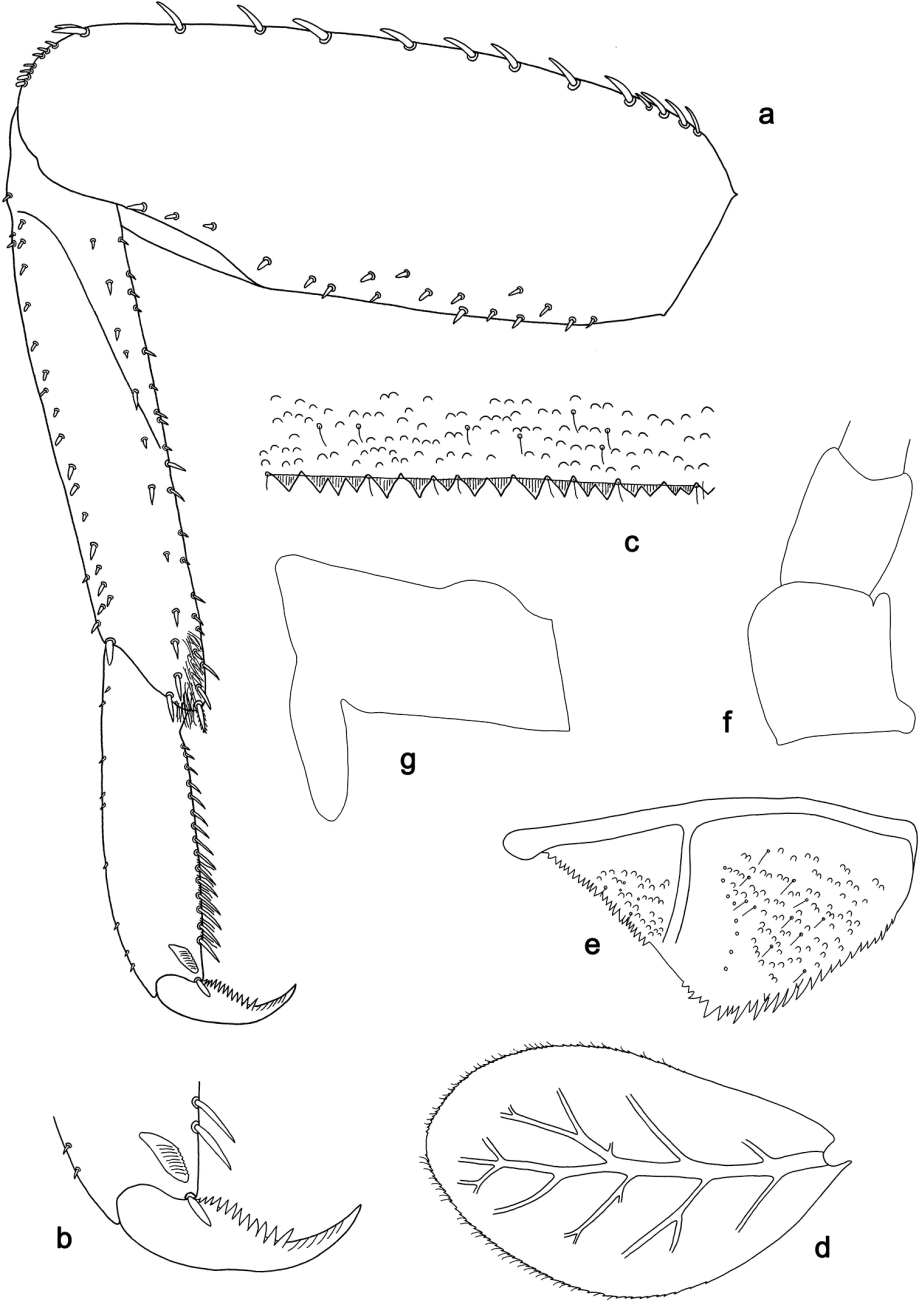
*Labiobaetis valdezorum* sp. nov., larva morphology **a** foreleg **b** fore claw **c** tergum IV **d** gill IV **e** paraproct **f** antennal scape **g** metanotum.

Figures 23 , 24 , 44a , 49a

##### Diagnosis.

**Larva.** Following combination of characters: A) dorsal surface of labrum with submarginal arc of one plus 9–12 feathered setae; B) labial palp segment II with broad, thumb-like distomedial protuberance; C) fore femur rather broad, length ca. 3 × maximum width, dorsal margin with 12–15 curved, spine-like setae, fore tarsus broad with dorsal and ventral margins slightly convex; D) claw with eleven or twelve denticles; E) paraproct distally not expanded, with 23–32 stout marginal spines and some additional submarginal spines.

##### Description.

**Larva** (Figs 23, 24, 44a). Body length 5.5–5.9 mm. Cerci ca. 2/3 of body length. Paracercus ca. 2/3 of cerci length. Antenna approx. 2.5 × as long as head length.

***Colouration*.** Head, thorax, and abdomen dorsally light brown, with darker pattern as in Fig. 44a, abdominal segments VIII and IX dark brown. Fore protoptera light brown, basally darker, with bright striation. Head, thorax, and abdomen ventrally light brown, frons and genae darker brown, abdominal segments VIII and IX dark brown. Legs light brown, femur with distomedial dark brown spot, apically brown, tarsus dorsodistally dark brown. Caudalii light brown with a dark brown band at ca. 1/3 of cerci length.

***Antenna*** (Fig. 24f) with scape and pedicel subcylindrical, with well-developed distolateral process at scape.

***Labrum*** (Fig. 23a). Rectangular, length 0.8 × maximum width. Distal margin with medial emargination and a small process. Dorsally with medium, fine, simple setae scattered over surface; submarginal arc of setae composed of one plus 9–12 long, feathered setae. Ventrally with marginal row of setae composed of lateral and anterolateral long, feathered setae and medial long, bifid, pectinate setae; ventral surface with ca. eight short, spine-like setae near lateral and anterolateral margin.

***Right mandible*** (Fig. 23b, c). Incisor and kinetodontium fused. Incisor with five denticles; kinetodontium with three denticles, inner margin of innermost denticle with a row of thin setae. Prostheca robust, apically denticulate. Margin between prostheca and mola slightly convex. Tuft of setae at apex of mola present.

***Left mandible*** (Fig. 23d, e). Incisor and kinetodontium fused. Incisor with four denticles; kinetodontium with three denticles. Prostheca robust, apically with small denticles and comb-shaped structure. Margin between prostheca and mola slightly convex, with minute denticles toward subtriangular process. Subtriangular process long and slender, above level of area between prostheca and mola. Denticles of mola apically constricted. Tuft of setae at apex of mola present.

Both mandibles with lateral margins almost straight. Basal half with fine, simple setae scattered over dorsal surface.

***Hypopharynx and superlinguae*** (Fig. 23f). Lingua shorter than superlinguae. Lingua longer than broad; medial tuft of stout setae well developed, short; distal half laterally expanded. Superlinguae distally rounded; lateral margin rounded; fine, long, simple setae along distal margin.

***Maxilla*** (Fig. 23g). Galea-lacinia ventrally with two simple, apical setae under canines. Inner dorsal row of setae with three denti-setae, distal denti-seta tooth-like, middle and proximal denti-setae slender, bifid and pectinate. Medially with one bipectinate, spine-like seta and five medium to long, simple setae. Maxillary palp slightly longer than length of galea-lacinia; 2-segmented; palp segment II 1.4 × length of segment I; setae on maxillary palp fine, simple, scattered over surface of segments I and II; apex of last segment rounded, with slight excavation at inner distolateral margin.

***Labium*** (Fig. 23h). Glossa basally broad, narrowing toward apex; shorter than paraglossa; inner margin with ca. ten spine-like setae increasing in length distally; apex with two long and one medium, robust, pectinate setae and one short, robust seta; outer margin with eight long, spine-like setae; ventral surface with short, fine, simple and short, spine-like setae. Paraglossa sub-rectangular, curved inward; apex rounded; with three rows of long, robust, distally pectinate setae in apical area and one medium, simple seta in anteromedial area; dorsally with a row of six or seven long, spine-like, simple setae near inner margin. Labial palp with segment I 0.8 × length of segments II and III combined. Segment I ventrally with short, fine, simple setae. Segment II with broad thumb-like distomedial protuberance; distomedial protuberance 0.6 × width of base of segment III; ventral surface with short, fine, simple setae; dorsally with a row of three or four spine-like, simple setae near outer margin. Segment III conical; apex slightly pointed; length subequal to width; ventrally covered with short, spine-like, simple setae and short, fine, simple setae.

***Hind protoptera*** (Fig. 24g) well developed.

***Foreleg*** (Fig. 24a, b). Ratio of foreleg segments 1.2:1.0:0.6:0.2. ***Femur*.** Length ca. 3 × maximum width. Dorsal margin with a row of 12–15 curved, spine-like setae, sometimes a few additional setae near margin in basal area; length of setae 0.14 × maximum width of femur. Apex rounded, with a pair of spine-like setae and some short, stout setae. Many stout, lanceolate setae scattered along ventral margin; femoral patch absent. ***Tibia*.** Dorsal margin with a row of short, spine-like setae, on apex one longer, spine-like seta. Ventral margin with a row of mainly short, spine-like setae, on apex some longer and partly bipectinate setae and a tuft of fine, simple setae. Anterior surface scattered with stout, lanceolate setae. Patellotibial suture present on basal half. ***Tarsus*.** Dorsal margin with a row of short, stout setae. Ventral margin with a row of curved, spine-like setae and in distal area fine simple setae. Claw with one row of eleven or twelve denticles; with ca. six stripes; subapical setae absent.

***Terga*** (Fig. 24c). Surface with irregular rows of U-shaped scale bases and scattered fine, simple setae. Posterior margin of tergum IV with triangular spines, wider than long.

***Gills*** (Fig. 24d). Present on segments I–VII. Margin with small denticles intercalating fine simple setae. Tracheae extending from main trunk to inner and outer margins. Gill I as long as length of segment II. Gill IV as long as length of segments V and half of VI combined. Gill VII as long as length of segment VIII.

***Paraproct*** (Fig. 24e). Distally not expanded, with 23–32 stout, marginal spines and some submarginal spines. Surface scattered with U-shaped scale bases and fine, simple setae. Cercotractor with numerous small, marginal spines, apically pointed.

##### Etymology.

Dedicated to Dr. Emma Aguada Valdez, Mr. Rolando Valdez and Mr. Francis Paolo Valdez for their generous help and support in the scientific projects of the AdMU Biodiversity Laboratory.

##### Distribution.

Philippines: Negros and Cebu (Fig. 49a).

##### Biological aspects.

The specimens were collected at altitudes from 150 m to 480 m, on bottom gravel or submerged wood in runs or riffles.

##### Type material.

***Holotype*.** Philippines • larva; Negros Oriental, Valencia, Casaroro River downstream; 09°18'N, 123°14'E; 150 m; 01.IX.2019; leg. Garces and Pelingen; on slide; GenBank. MT830963; GBIFCH 00654888; PNM. ***Paratypes*.** Philippines • 24 larvae; same data as holotype; 3 on slides; GenBank. MT830965, MT830966; GBIFCH 00654879, GBIFCH 00592277, GBIFCH 00654880; ZSM; 1 on slide; GenBank: MT830964; GBIFCH 00654882; AdMU; 7 in alcohol; GBIFCH 00515419; ZSM; 13 in alcohol; GBIFCH 00515418, GBIFCH 00515420; AdMU • 1 larva; Negros Oriental, Valencia, Apolong, Casaroro River upstream; 09°17'N, 123°13'E; 470 m; 01.IX.2019; leg. Garces and Pelingen; in alcohol; GBIFCH 00515421; ZSM • 15 larvae; Cebu, Cebu City, Lusaran; 10°28'13"N, 123°52'26"E; 200 m; 16.IX.1996; leg. Mendoza; 1 on slide; GenBank: MT830967; GBIFCH 00763671; ZSM; 10 in alcohol; GBIFCH 00515422, GBIFCH 00515423, GBIFCH 00515424; AdMU; 4 in alcohol; GBIFCH 00515425; ZSM.

#### Labiobaetis wantzeni sp. nov.

Taxon classificationAnimaliaEphemeropteraBaetidae

6237AA1C-6D82-56E9-B90B-FEE3B1EB0CC4

http://zoobank.org/1651E9BC-B331-4AB0-85C3-8BFB5746B3EC

**Figure 25.**
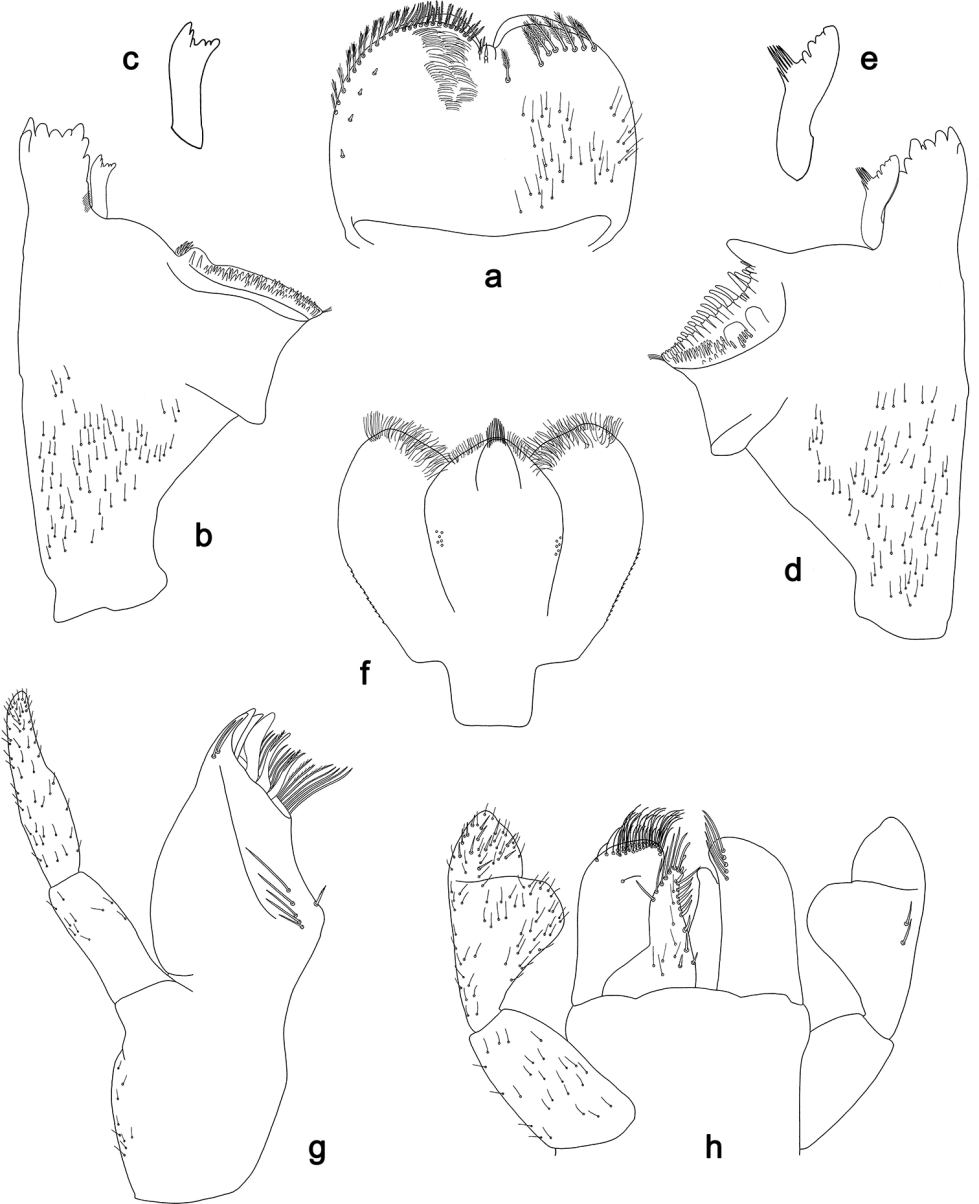
*Labiobaetis wantzeni* sp. nov., larva morphology **a** labrum **b** right mandible **c** right prostheca **d** left mandible **e** left prostheca **f** hypopharynx and superlinguae **g** maxilla **h** labium.

**Figure 26.**
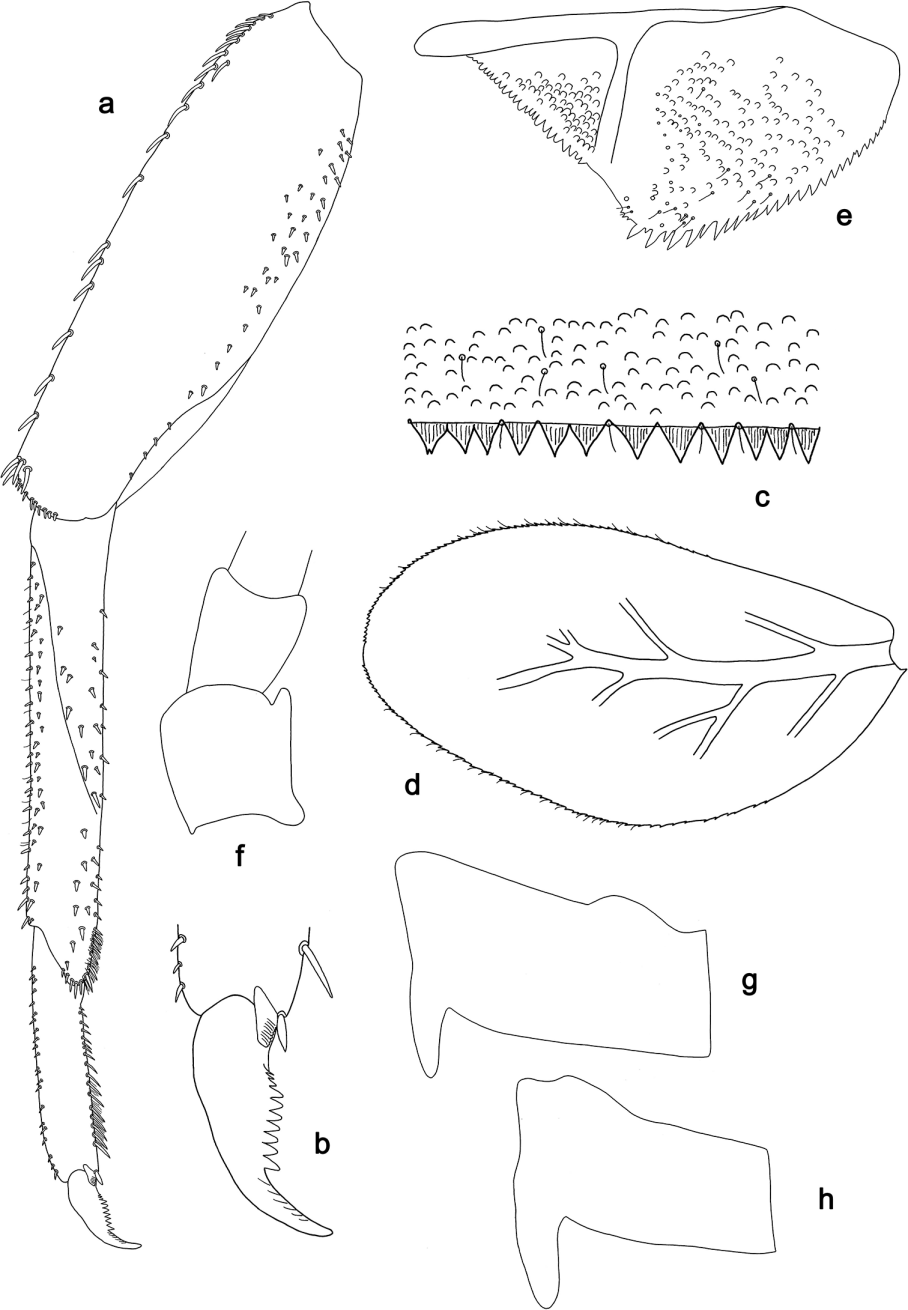
*Labiobaetis wantzeni* sp. nov., larva morphology **a** foreleg **b** fore claw **c** tergum IV **d** gill IV **e** paraproct **f** antennal scape **g** metanotum **h** metanotum.

Figures 25 , 26 , 44b , 49a

##### Diagnosis.

**Larva.** Following combination of characters: A) dorsal surface of labrum with submarginal arc of one plus 7–10 long, feathered setae; B) labial palp segment II with a broad, thumb-like distomedial protuberance, segment III conical; C) fore femur rather broad, length ca. 3 × maximum width, dorsal margin with a row of ca. 18 curved, spine-like setae; D) claw with 10–13 denticles; E) hind protoptera medium developed; F) paraproct distally not expanded, with ca. 30 stout marginal spines.

##### Description.

**Larva** (Figs 25, 26, 44b). Body length 5.2–7.1 mm. Cerci ca. 2/3 of body length. Paracercus ca. 2/3 of cerci length. Antenna approx. 2.5 × as long as head length.

***Colouration*.** Head dorsally light brown, thorax and abdomen dorsally mainly dark brown with pattern as in Fig. 44b, abdominal segments I, VI, and X light brown; fore protoptera basally dark brown, distally light brown with bright striation. Head, thorax, and abdomen ventrally mainly light brown, genae dark brown, abdominal segments laterally with darker areas and abdominal segments VII–IX dark brown. Legs light brown, femur with distomedial and apical dark brown spots, tibia distally with dark brown area. Caudalii light brown with a brown band at ca. 1/3 of cerci length, cerci distally brown.

***Antenna*** (Fig. 26f) with scape and pedicel subcylindrical, with well-developed distolateral process at scape.

***Labrum*** (Fig. 25a). Rectangular, length 0.7 × maximum width. Distal margin with medial emargination and a small process. Dorsally with medium, fine, simple setae scattered over surface; submarginal arc of setae composed of one plus 7–10 long, feathered setae. Ventrally with marginal row of setae composed of lateral and anterolateral long, feathered setae and medial long, bifid, pectinate setae; ventral surface with ca. four short, spine-like setae near lateral and anterolateral margin.

***Right mandible*** (Fig. 25b, c). Incisor and kinetodontium fused. Incisor with five denticles; kinetodontium with three denticles, inner margin of innermost denticle with a row of thin setae. Prostheca robust, apically denticulate. Margin between prostheca and mola slightly convex. Tuft of setae at apex of mola present.

***Left mandible*** (Fig. 25d, e). Incisor and kinetodontium fused. Incisor with five denticles; kinetodontium with three denticles. Prostheca robust, apically with small denticles and comb-shaped structure. Margin between prostheca and mola slightly convex. Subtriangular process long and slender, above level of area between prostheca and mola. Denticles of mola apically constricted. Tuft of setae at apex of mola present.

Both mandibles with lateral margins almost straight. Basal half with fine, simple setae scattered over dorsal surface.

***Hypopharynx and superlinguae*** (Fig. 25f). Lingua approx. as long as superlinguae. Lingua longer than broad; medial tuft of stout setae well developed, short; distal half laterally expanded. Superlinguae distally rounded; lateral margin rounded; fine, long, simple setae along distal margin.

***Maxilla*** (Fig. 25g). Galea-lacinia ventrally with two simple, apical setae under canines. Inner dorsal row of setae with three denti-setae, distal denti-seta tooth-like, middle and proximal denti-setae slender, bifid and pectinate. Medially with one bipectinate, spine-like seta and five medium to long, simple setae. Maxillary palp 1.2 × as long as length of galea-lacinia; 2-segmented; palp segment II 1.5 × length of segment I; setae on maxillary palp fine, simple, scattered over surface of segments I and II; apex of last segment rounded, with excavation at inner distolateral margin.

***Labium*** (Fig. 25h). Glossa basally broad, narrowing toward apex; shorter than paraglossa; inner margin with ca. 8 spine-like setae increasing in length distally; apex with two long, one medium and one short, robust setae; outer margin with five long, spine-like setae; ventral surface with short, fine, simple and short, spine-like setae. Paraglossa sub-rectangular, curved inward; apex rounded; with three rows of long, robust, distally pectinate setae in apical area and one or two medium, simple setae in anteromedial area; dorsally with a row of five long, spine-like, simple setae near inner margin. Labial palp with segment I 0.8 × length of segments II and III combined. Segment I ventrally with short, fine, simple setae. Segment II with broad thumb-like distomedial protuberance; distomedial protuberance 0.5 × width of base of segment III; ventral surface with short, fine, simple setae; dorsally with two or three spine-like, simple setae near outer margin. Segment III conical; apex rounded; length 0.9 × width; ventrally covered with short, spine-like, simple setae and short, fine, simple setae.

***Hind protoptera*** (Fig. 26g, h) medium to well developed.

***Foreleg*** (Fig. 26a, b). Ratio of foreleg segments 1.3:1.0:0.5:0.2. ***Femur*.** Length ca. 3 × maximum width. Dorsal margin with a row of 15–18 curved, spine-like setae, sometimes a few extra setae basally near margin; length of setae 0.14 × maximum width of femur. Apex rounded, with one or two pairs of spine-like setae and some short, stout setae. Many stout, lanceolate setae scattered along ventral margin; femoral patch absent. ***Tibia*.** Dorsal margin with a row of short, spine-like setae and fine, simple setae, near margin another row of short, spine-like setae. Ventral margin with a row of short, spine-like setae, on apex some longer spine-like setae and a tuft of fine, simple setae. Anterior surface scattered with stout, lanceolate setae. Patellotibial suture present on basal 2/3. ***Tarsus*.** Dorsal margin with a row of short, stout setae. Ventral margin with a row of curved, spine-like setae and in distal area fine simple setae. Claw with one row of 10–13 denticles; distally pointed; with ca. six stripes; subapical setae absent.

***Terga*** (Fig. 26c). Surface with irregular rows of U-shaped scale bases and scattered fine, simple setae. Posterior margin of tergum IV with triangular spines, mainly longer than wide.

***Gills*** (Fig. 26d). Present on segments I–VII. Margin with small denticles intercalating fine simple setae. Tracheae extending from main trunk to inner and outer margins. Gill I ca. 2/3 length of segment II. Gill IV as long as length of segments V and half VI combined. Gill VII as long as length of segments VIII and 1/4 IX combined.

***Paraproct*** (Fig. 26e). Distally not expanded, with ca. 30 stout, marginal spines. Surface scattered with U-shaped scale bases, fine, simple setae and micropores. Cercotractor with numerous small, marginal spines.

##### Etymology.

Dedicated to Prof. Karl Matthias Wantzen (France), collector of some material, for his outstanding contribution to freshwater ecological research and conservation.

##### Distribution.

Philippines: Camiguin and Mindanao (Fig. 49a).

##### Biological aspects.

The specimens were collected at altitudes from sea level to 900 m, on bottom gravel, submerged wood, leaf packs or grass bunches in runs or riffles.

##### Type material.

***Holotype*.** Philippines • larva; Camiguin, Sagay, Bonbon, lower Binangawan River; 09°06'39"N 124°43'45"E; 30 m; 09.XII.2018; leg. Freitag and Wantzen; on slide; GenBank: MT830968; GBIFCH 00654898; PNM. ***Paratypes*.** Philippines • 8 larvae; same data as holotype; 3 on slides; GenBank: MT830973, MT830969, MT830972; GBIFCH 00654900, GBIFCH 00654897, GBIFCH 00654896; ZSM; 1 on slide; GBIFCH 00592276; AdMU; 2 in alcohol; GBIFCH 00515412, GBIFCH 00515413; AdMU; 2 in alcohol; GBIFCH 00515416, GBIFCH 00515417; ZSM • 2 larvae; Camiguin, Looc /Tuasan R, ca. 9.5 km SE Mambajao; 09°12'N, 124°41'E; 240 m; 08.XII.2018; leg. Freitag; 1 on slide; GBIFCH 00515522; AdMU; 1 on slide; GenBank: MT830970; GBIFCH 00763641; ZSM • 1 larva; Camiguin, Mt. Mabajao Sagay, upstream Binangawan Falls; 09°09'25"N, 124°43'57"E; 900 m; 09.XII.2018; leg. Freitag; in alcohol; GBIFCH 00515414; AdMU • 7 larvae; Mindanao, Ozamis, Tangub, Labo River; 08°09'42"N, 124°42'28"E; 470 m; 13.IV.1994; leg. Mendoza; 1 on slide; GenBank: MT830971; GBIFCH 00763642; ZSM; 6 in alcohol; GBIFCH 00515415; AdMU.

### *Labiobaetis sumigarensis* group of species (Kaltenbach and Gattolliat 2019)

Following combination of characters: A) dorsal surface of labrum with submarginal arc of clavate, apically smooth setae; B) labial palp segment II with large, lobed or thumb-like distomedial protuberance, outer margin of protuberance predominantly concave (sometimes with hook-like modification of the protuberance); C) left mandible without setae at apex of mola, with minute denticles between prostheca and mola; D) six pairs of gills; E) hind protoptera absent; F) distolateral process at scape poorly developed or absent; G) patellotibial suture short, on basal 1/3 area of tibia; H) colour of larvae dorsally uniform brown.

#### Labiobaetis molawinensis

Taxon classificationAnimaliaEphemeropteraBaetidae

(Müller-Liebenau, 1982)

EE940A7A-37BC-5D6A-8D71-6F07326FA8AC

Figures 27 , 49b

##### Diagnosis.

**Larva.** Following combination of characters: A) dorsal surface of labrum with submarginal arc of ca. 15 clavate setae; B) labial palp segment II with a large, lobed distomedial protuberance, segment III slightly pentagonal, apically slightly truncate; C) left mandible without setae at apex of mola; D) fore femur rather slender, length 3.6 × maximum width, dorsal margin with a row of ca. ten curved, spine-like setae; E) tarsal claw with ca. eleven denticles; F) paraproct distally not expanded, with > 40 stout marginal spines.

##### Examined material.

***Paratype*.** Philippines • 1 larva; rapids, Molawin Creek, college, Laguna; 28.VII.1977; leg. C.R. Realon; Coll. Pescador; on slide; ZSM.

##### Other material.

Philippines • 1 larva; Luzon, Laguna, Los Banos, UP Campus, Molawin River; 14°10'05"N, 121°11'44"E; 29.IX.1998; leg. Mendoza; on slide; GBIFCH 00654910; AdMU • 1 larva; Luzon, Laguna, Samil River; 14°08'N, 121°31'E; 27.VI.2018; 370 m; leg. Freitag et al.; on slide; GBIFCH 00654912; ZSM • 1 larva; Luzon, Aurora, Baler, Cemento, rocky coast; 15°45'21"N, 121°34'46"E; 0 m; 04.II.1998; leg. Mendoza; on slide; GBIFCH 00654892; AdMU.

**Figure 27.**
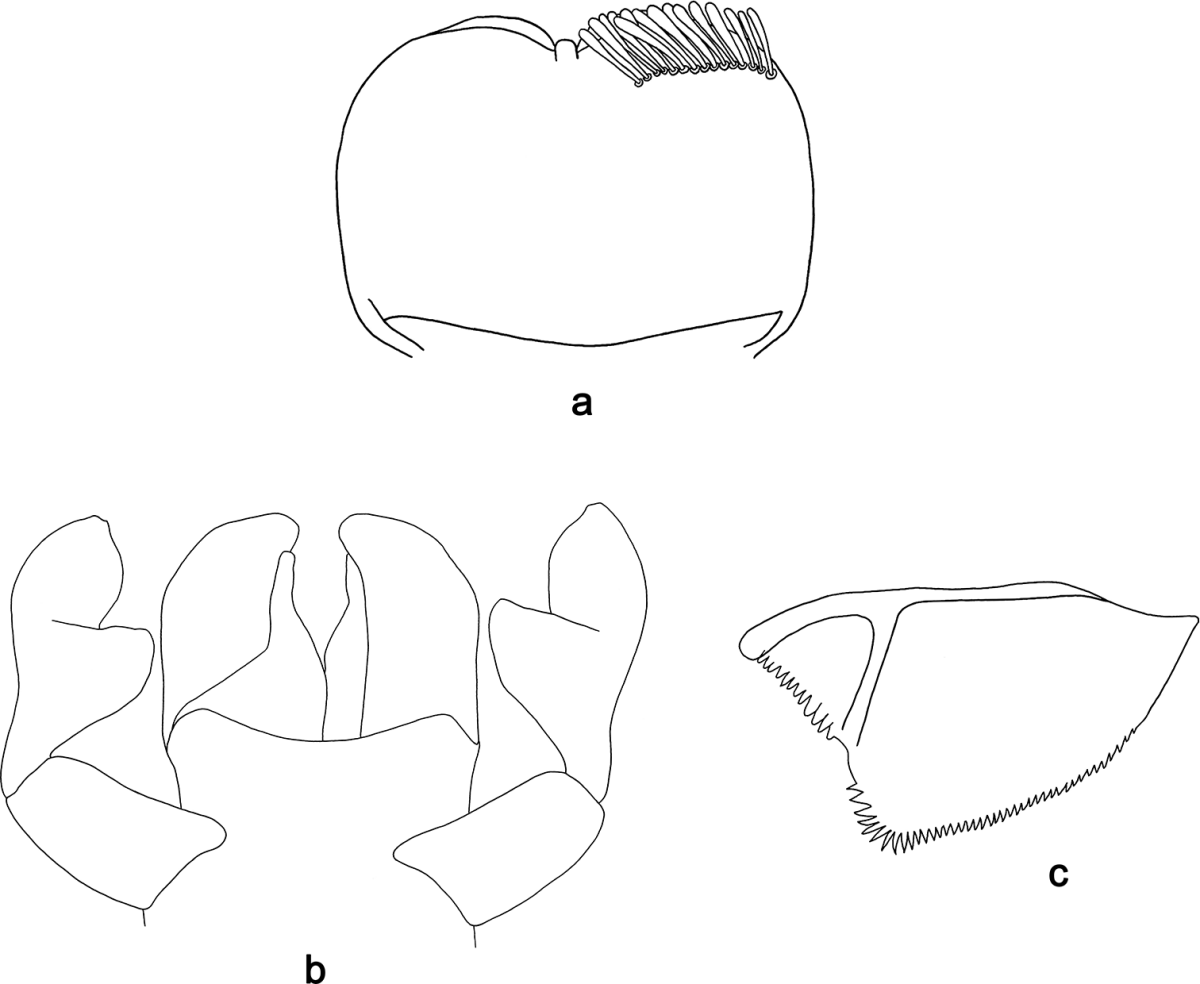
*Labiobaetis molawinensis*, larva morphology **a** labrum **b** labium **c** paraproct.

#### Labiobaetis sumigarensis

Taxon classificationAnimaliaEphemeropteraBaetidae

(Müller-Liebenau, 1982)

450DB6E8-F831-5813-B7A4-0D0BA755A325

Figures 28 , 49b

##### Diagnosis.

**Larva.** Following combination of characters: A) dorsal surface of labrum with submarginal arc of ca. 26 clavate setae; B) labial palp segment II with a hook-like distomedial protuberance, segment III slightly pentagonal, apically slightly pointed; C) left mandible without setae at apex of mola; D) fore femur rather broad, length 3.4 × maximum width, dorsal margin with ca. 15 curved, spine-like setae; E) tarsal claw with ca. ten denticles; F) paraproct slightly expanded, with 35–39 stout marginal spines, some with split tips.

##### Examined material.

***Holotype*.** Philippines • 1 larva; Mountain Prov., Sumigar Stream, Sumigar, Banaue; 03.X.1967; leg. Pescador; on slide; ZSM.

##### Other material.

Philippines • 1 larva; Luzon, Ifugao, Banaue, Sumigar Bridge; 16°59'37"N, 121°02'51"E; 1700 m; IX.1997; leg. Mey; on slide; GBIFCH 00592357; AdMU.

**Figure 28.**
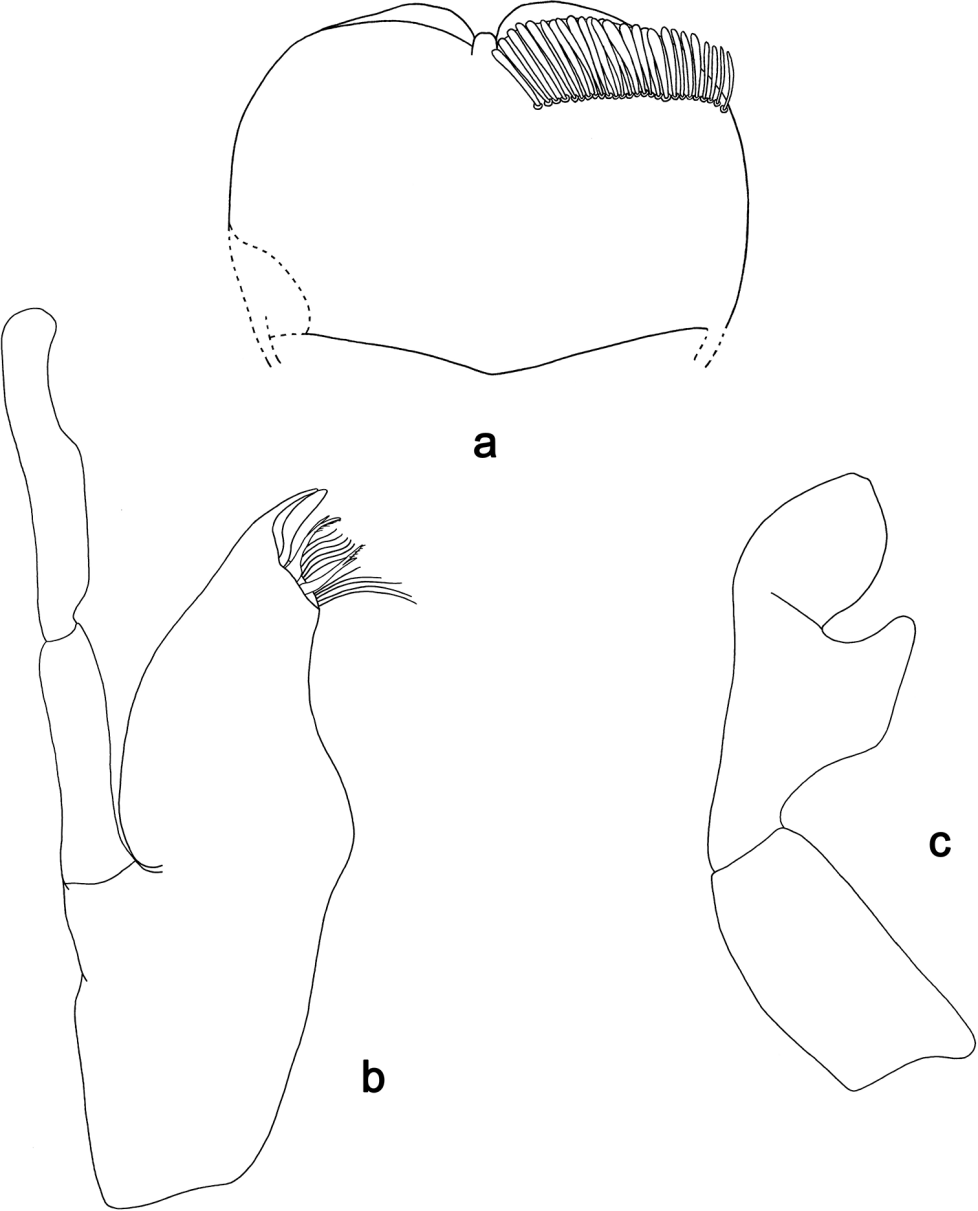
*Labiobaetis sumigarensis*, larva morphology **a** labrum **b** maxilla **c** labial palp.

#### Labiobaetis baganii sp. nov.

Taxon classificationAnimaliaEphemeropteraBaetidae

BCD12B82-DB53-5209-8594-BDAB412B3C5B

http://zoobank.org/69A8B24A-CED0-4E1A-AC2D-59D31B156558

**Figure 29.**
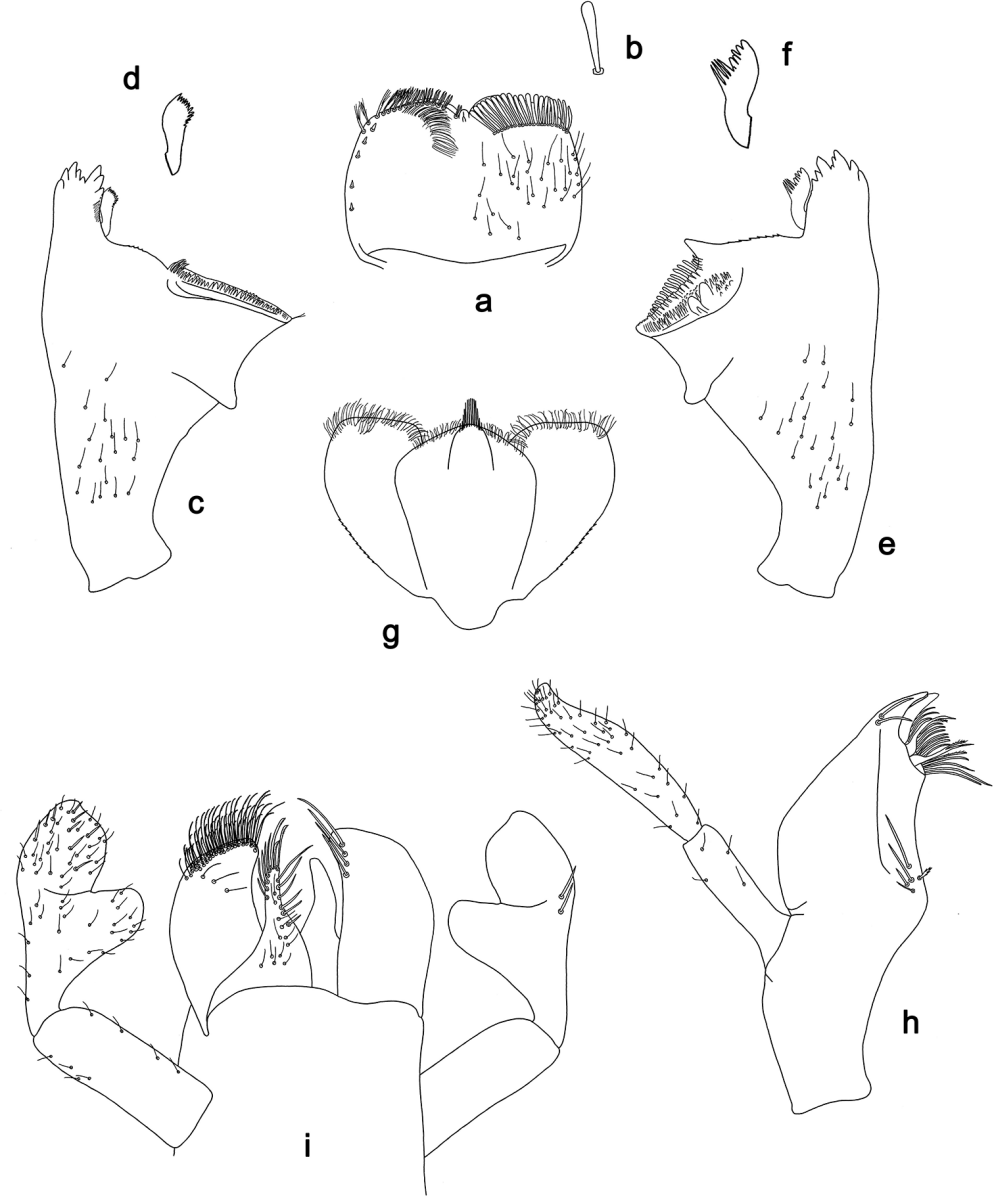
*Labiobaetis baganii* sp. nov., larva morphology **a** labrum **b** seta of arc on dorsal surface of labrum **c** right mandible **d** right prostheca **e** left mandible **f** left prostheca **g** hypopharynx and superlinguae **h** maxilla **i** labium.

**Figure 30.**
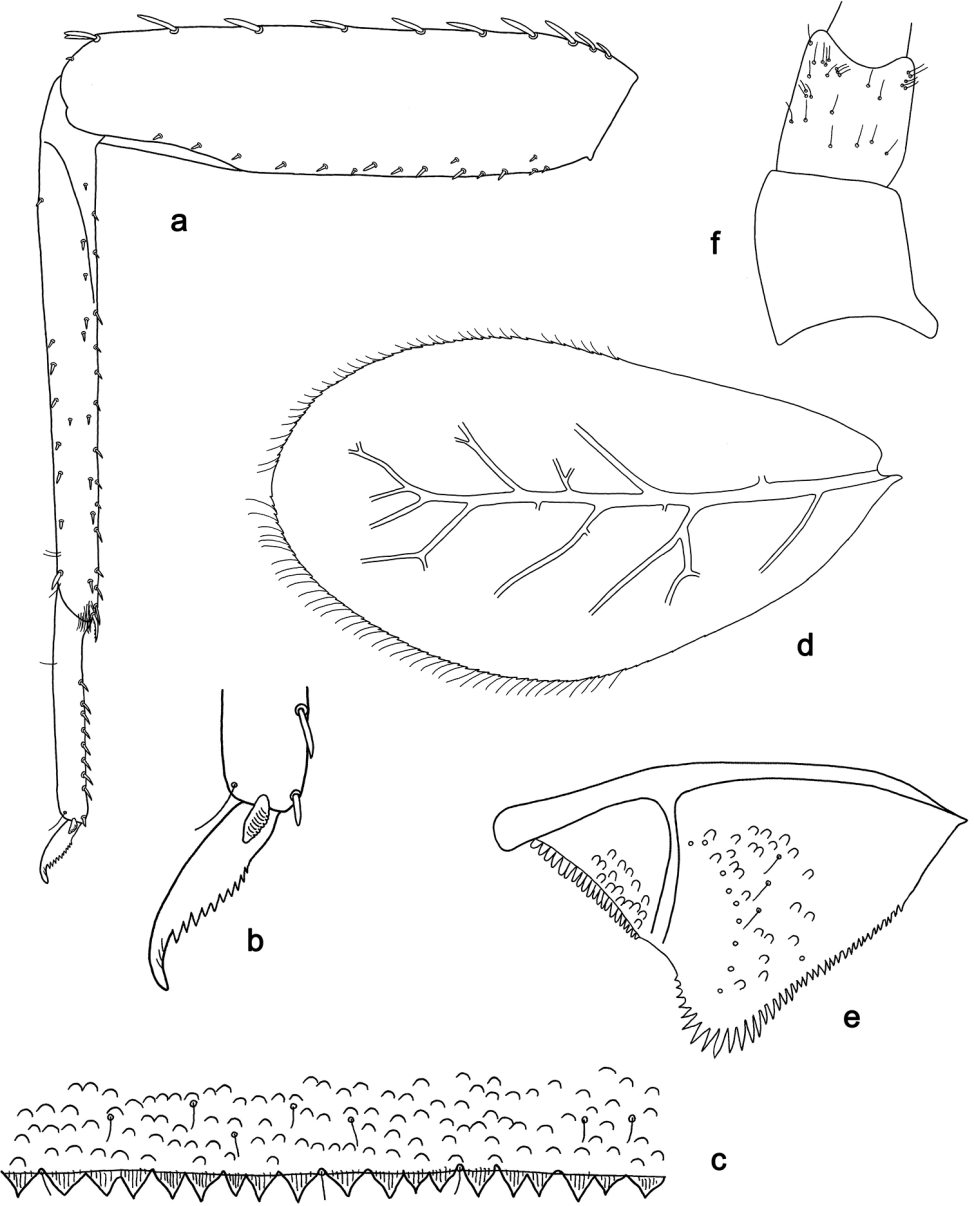
*Labiobaetis baganii* sp. nov., larva morphology **a** foreleg **b** fore claw **c** tergum IV **d** gill IV **e** paraproct **f** antennal scape.

Figures 29 , 30 , 44c , 49b

##### Diagnosis.

**Larva.** Following combination of characters: A) dorsal surface of labrum with submarginal arc of 17–21 long, clavate setae; B) labial palp segment II with a thumb-like distomedial protuberance, segment III slightly pentagonal; C) left mandible without setae at apex of mola; D) fore femur rather slender, length ca. 4 × maximum width, dorsal margin with 8–11 curved, spine-like setae; E) paraproct distally expanded, with 39–43 stout, marginal spines.

##### Description.

**Larva** (Figs 29, 30, 44c). Body length 3.1–5 mm. Cerci ca. 1/2 of body length. Paracercus: ca. 2/3 of cerci length. Antenna approx. twice as long as head length.

***Colouration*.** Head, thorax, and abdomen dorsally brown, thorax with pattern as in Fig. 44c, fore protoptera brown. Head, thorax, and abdomen ventrally light brown. Legs light brown, femur with a brown distomedial spot connected to a brown streak along ventral margin, apex brown. Caudalii light brown.

***Antenna*** (Fig. 30f) with scape and pedicel subcylindrical, without distolateral process at scape.

***Labrum*** (Fig. 29a, b). Rectangular, length 0.7 × maximum width. Distal margin with medial emargination and a small process. Dorsally with medium to long, fine, simple setae scattered over surface; submarginal arc of setae composed of 17–21 long, clavate setae. Ventrally with marginal row of setae composed of anterolateral long, feathered setae and medial long, bifid setae; ventral surface with ca. five short, spine-like setae near lateral and anterolateral margin.

***Right mandible*** (Fig. 29c, d). Incisor and kinetodontium fused. Incisor with five denticles; kinetodontium with three denticles, inner margin of innermost denticle with a row of thin setae. Prostheca robust, apically denticulate. Margin between prostheca and mola slightly convex, with minute denticles. Tuft of setae at apex of mola present.

***Left mandible*** (Fig. 29e, f). Incisor and kinetodontium fused. Incisor with five denticles; kinetodontium with three denticles. Prostheca robust, apically with small denticles and comb-shaped structure. Margin between prostheca and mola straight, with minute denticles towards subtriangular process. Subtriangular process long and slender, above level of area between prostheca and mola. Denticles of mola apically constricted. Tuft of setae at apex of mola absent.

Both mandibles with lateral margins almost straight. Basal half with fine, simple setae scattered over dorsal surface.

***Hypopharynx and superlinguae*** (Fig. 29g). Lingua approx. as long as superlinguae. Lingua longer than broad; medial tuft of stout setae well developed; distal half laterally expanded. Superlinguae distally straight; lateral margin rounded; fine, long, simple setae along distal margin.

***Maxilla*** (Fig. 29h). Galea-lacinia ventrally with two simple, apical setae under canines. Inner dorsal row of setae with three denti-setae, distal denti-seta tooth-like, middle and proximal denti-setae slender, bifid and pectinate. Medially with one bipectinate, spine-like seta and three or four medium to long, simple setae. Maxillary palp 1.4 × as long as length of galea-lacinia; 2-segmented; palp segment II 1.5 × length of segment I; setae on maxillary palp fine, simple, scattered over surface of segments I and II; apex of last segment rounded, with excavation at inner distolateral margin.

***Labium*** (Fig. 29i). Glossa basally broad, narrowing toward apex; shorter than paraglossa; inner margin with six spine-like setae increasing in length distally; apex with two long and one medium, robust, pectinate setae; outer margin with five spine-like setae increasing in length distally; ventral surface with fine, simple, scattered setae. Paraglossa sub-rectangular, curved inward; apex rounded; with three rows of long, robust, distally pectinate setae in apical area and a row of three or four medium, simple setae in anteromedial area; dorsally with a row of four long, spine-like, simple setae near inner margin. Labial palp with segment I 0.8 × length of segments II and III combined. Segment I ventrally with short, fine, simple setae. Segment II with thumb-like distomedial protuberance; distomedial protuberance 0.7 × width of base of segment III; ventral surface with short, fine, simple setae; dorsally with two long, spine-like, simple setae near outer margin. Segment III slightly pentagonal; apex rounded; length 1.3 × width; ventrally covered with short, spine-like, simple setae and short, fine, simple setae.

***Hind protoptera*** absent.

***Foreleg*** (Fig. 30a, b). Ratio of foreleg segments 1.1:1.0:0.4:0.1. ***Femur*.** Length ca. 4 × maximum width. Dorsal margin with 8–11 long, curved, spine-like setae; length of setae 0.24 × maximum width of femur. Apex rounded, with a pair of long, curved, spine-like setae and one or two short, stout setae. Many stout, lanceolate setae scattered along ventral margin; femoral patch absent. ***Tibia*.** Dorsal margin with a row of short, spine-like setae, on apex one longer, spine-like seta. Ventral margin with a row of short, curved, spine-like setae, on apex some longer, partly bipectinate setae and a tuft of fine, simple setae. Anterior surface scattered with stout, lanceolate setae. Patellotibial suture present on basal 1/3. ***Tarsus*.** Dorsal margin with some fine, simple setae. Ventral margin with a row of curved, spine-like setae. Claw with one row of ten or eleven denticles; distally pointed; with ca. three stripes; subapical setae absent.

***Terga*** (Fig. 30c). Surface with irregular rows of U-shaped scale bases and scattered fine, simple setae. Posterior margin of tergum IV with triangular spines, wider than long.

***Gills*** (Fig. 30d). Present on segments II–VII. Margin with small denticles intercalating fine simple setae. Tracheae extending from main trunk to inner and outer margins. Gill IV as long as length of segments V and half VI combined. Gill VII little longer than length of segment VIII.

***Paraproct*** (Fig. 30e). Distally expanded, with 39–43 stout, marginal spines. Surface scattered with U-shaped scale bases, fine, simple setae and micropores. Cercotractor with numerous small, marginal spines.

##### Etymology.

Dedicated to Mr. Bagani Sularte (Philippines), outstanding illustrator and friend of one of the authors (JG), for support in her dissertation work.

##### Distribution.

Philippines: Mindanao and Camiguin (Fig. 49b).

##### Biological aspects.

The specimens were collected at altitudes from sea level to 660 m, mainly in submerged wood or leaf packs in the runs.

##### Type material.

***Holotype*.** Philippines • larva; Mindanao, Agusan N, Cabadbaran River; 09°10'15"N, 125°40'55"E; 240 m.; 03.VI.2018; leg. Freitag and Pangantihon; on slide; GenBank: MT830974; GBIFCH 00654895; PNM. ***Paratypes*.** Philippines • 2 larvae; same data as holotype; 1 on slide; GBIFCH 00592303; ZSM; 1 in alcohol; GBIFCH 00515483; AdMU • 1 larva; Mindanao, Agusan del Sur, San Francisco, Bayogan, Tagkunayan Creek; 08°28'N, 125°59'E; 120 m; 05.II.1998; leg. Mendoza; on slide; GBIFCH 00592317; AdMU • 1 larva; Mindanao, Agusan del Norte, Jabonga, Creek upstream of village near Mainit; 09°20'40"N, 125°30'50"E; 50 m; 02.IV.2019, leg. Freitag; in alcohol; GBIFCH 00515484; ZSM • 1 larva; Mindanao, Agusan N, Cabadbaran, Del Pilar, Payas River; 09°11'34"N, 125°36'34"E; 660 m; 23.VI.2018; leg. Pangantihon; in alcohol; GBIFCH 00515485; ZSM • 15 larvae; Camiguin, Sagay, Bonbon, lower Binangawan River; 09°06'39"N 124°43'45"E; 30 m; 09.XII.2018; leg. Freitag and Wantzen; 2 on slides; GenBank: MT830975; GBIFCH 00654899, GBIFCH 592326; ZSM; 2 in alcohol; GBIFCH 00515486, GBIFCH 00515488; AdMU; 11 in alcohol; GBIFCH 515487; AdMU.

#### Labiobaetis delocadoi sp. nov.

Taxon classificationAnimaliaEphemeropteraBaetidae

6691A0BB-0CFF-590B-9364-16B44221754F

http://zoobank.org/61631C84-8BAE-4C88-8CDE-AC051839D419

**Figure 31.**
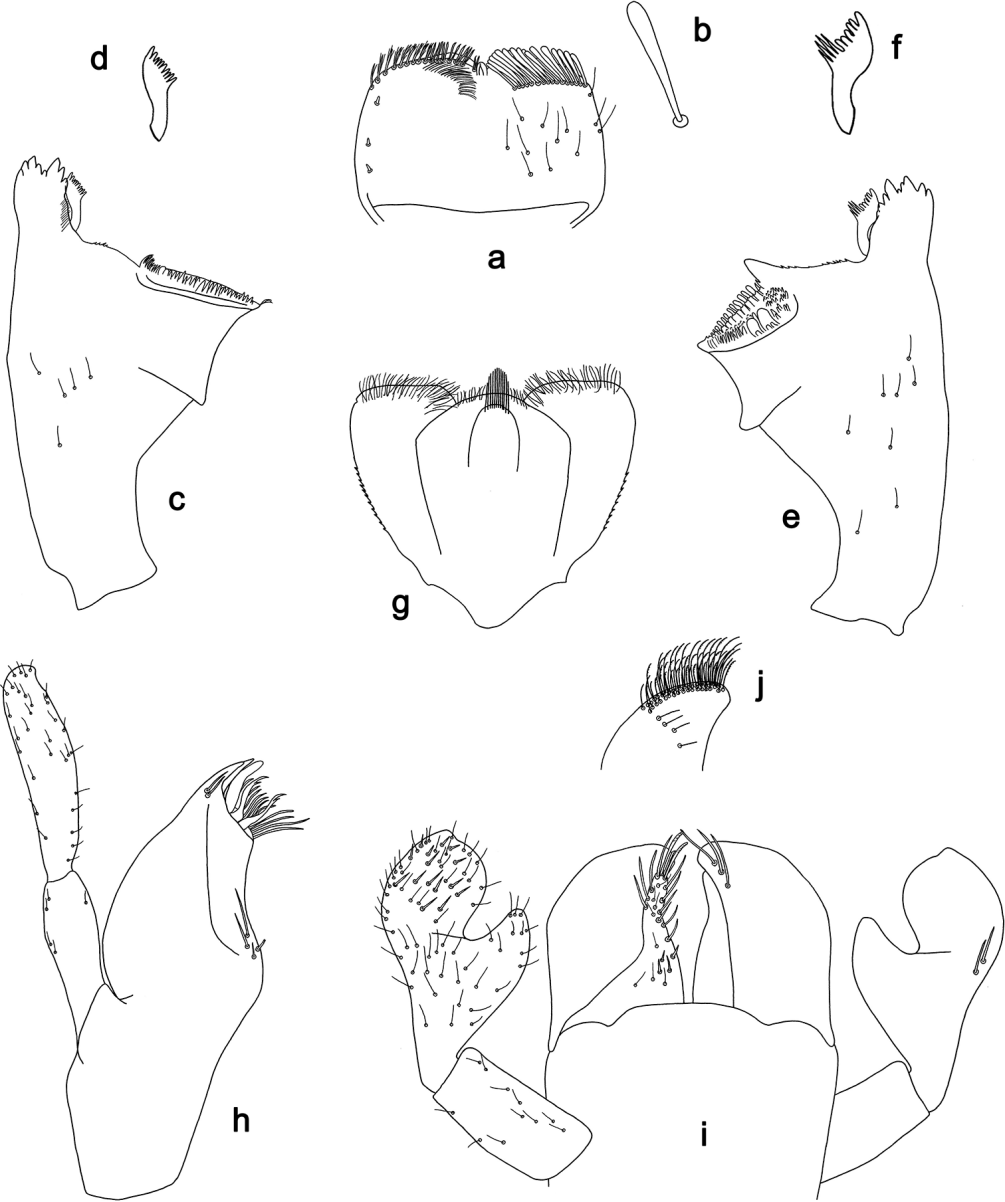
*Labiobaetis delocadoi* sp. nov., larva morphology **a** labrum **b** seta of arc on dorsal surface of labrum **c** right mandible **d** right prostheca **e** left mandible **f** left prostheca **g** hypopharynx and superlinguae **h** maxilla **i** labium **j** apex of paraglossa.

**Figure 32.**
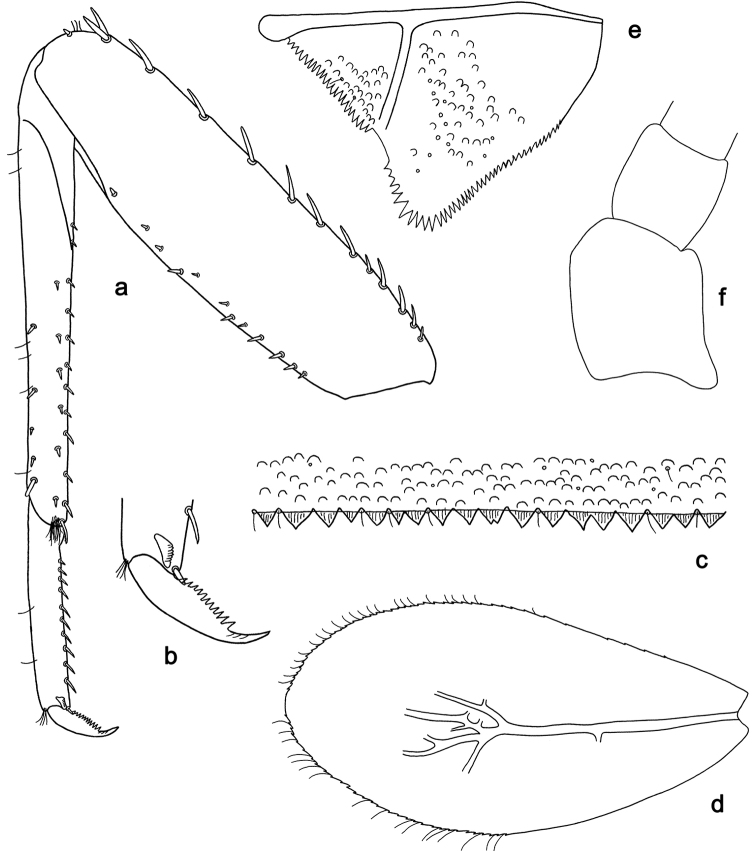
*Labiobaetis delocadoi* sp. nov., larva morphology **a** foreleg **b** fore claw **c** tergum IV **d** gill IV **e** paraproct **f** antennal scape.

Figures 31 , 32 , 44d , 49b

##### Diagnosis.

**Larva.** Following combinations of characters: A) dorsal surface of labrum with submarginal arc of 16–19 long, clavate setae; B) labial palp segment II with a hook-like distomedial protuberance, segment III slightly pentagonal; C) left mandible without setae at apex of mola; D) fore femur rather slender, length ca. 4 × maximum width, dorsal margin with ca. eleven curved, spine-like setae; E) paraproct distally expanded, with 36–39 stout, marginal spines.

##### Description.

**Larva** (Figs 31, 32, 44d). Body length 3.2–4.6 mm. Cerci ca. 2/3 of body length. Paracercus ca. 2/3 of cerci length. Antenna approx. twice as long as head length.

***Colouration*.** Head, thorax, and abdomen dorsally light brown, fore protoptera light brown with bright striation. Head, thorax, and abdomen ventrally light brown. Legs light brown, femur with a distomedial brown spot and a brown streak at dorsal margin, apex brown, tibia darker in distomedial area. Caudalii light brown, with a brown band at ca. 1/2 of cerci length.

***Antenna*** (Fig. 32f) with scape and pedicel subcylindrical, with very poorly developed distolateral process at scape.

***Labrum*** (Fig. 31a, b). Rectangular, length 0.7 × maximum width. Distal margin with medial emargination and a small process. Dorsally with medium, fine, simple setae scattered over surface; submarginal arc of setae composed of 16–19 long, clavate setae. Ventrally with marginal row of setae composed of anterolateral long, feathered setae and medial long, bifid setae; ventral surface with ca. three short, spine-like setae near lateral and anterolateral margin.

***Right mandible*** (Fig. 31c, d). Incisor and kinetodontium fused. Incisor with five denticles; kinetodontium with three denticles, inner margin of innermost denticle with a row of thin setae. Prostheca robust, apically denticulate. Margin between prostheca and mola slightly convex, with minute denticles. Tuft of setae at apex of mola present.

***Left mandible*** (Fig. 31e, f). Incisor and kinetodontium fused. Incisor with five denticles; kinetodontium with three denticles. Prostheca robust, apically with small denticles and comb-shaped structure. Margin between prostheca and mola straight, with minute denticles towards subtriangular process. Subtriangular process long and slender, above level of area between prostheca and mola. Denticles of mola apically constricted. Tuft of setae at apex of mola absent.

Both mandibles with lateral margins almost straight. Basal half with fine, simple setae scattered over dorsal surface.

***Hypopharynx and superlinguae*** (Fig. 31g). Lingua shorter than superlinguae. Lingua longer than broad; medial tuft of stout setae well developed; distal half laterally expanded. Superlinguae distally straight; lateral margin rounded; fine, long, simple setae along distal margin.

***Maxilla*** (Fig. 31h). Galea-lacinia ventrally with two simple, apical setae under canines. Inner dorsal row of setae with three denti-setae, distal denti-seta tooth-like, middle and proximal denti-setae slender, bifid and pectinate. Medially with one bipectinate, spine-like seta and three or four medium to long, simple setae. Maxillary palp 1.3 × as long as length of galea-lacinia; 2-segmented; palp segment II 1.6 × length of segment I; setae on maxillary palp fine, simple, scattered over surface of segments I and II; apex of last segment rounded, with slight excavation at inner distolateral margin.

***Labium*** (Fig. 31i, j). Glossa basally broad, narrowing toward apex; shorter than paraglossa; inner margin with ca. seven spine-like setae increasing in length distally; apex with two long and one medium, robust, pectinate setae; outer margin with four or five long, spine-like setae; ventral surface with short, fine, simple and short, spine-like setae. Paraglossa sub-rectangular, curved inward; apex rounded; with three rows of long, robust, distally pectinate setae in apical area and a row of four medium, simple setae in anteromedial area; dorsally with a row of three long, spine-like setae near inner margin. Labial palp with segment I 0.6 × length of segments II and III combined. Segment I ventrally with short, fine, simple setae. Segment II with hook-like distomedial protuberance; distomedial protuberance 0.8 × width of base of segment III; ventral surface with short, fine, simple setae; dorsally with one or two long, spine-like setae near outer margin. Segment III slightly pentagonal; apex slightly pointed; length 1.3 × width; ventrally covered with short, spine-like, simple setae and short, fine, simple setae.

***Hind protoptera*** absent.

***Foreleg*** (Fig. 32a, b). Ratio of foreleg segments 1.1:1.0:0.4:0.2. ***Femur*.** Length ca. 4 × maximum width. Dorsal margin with ten or eleven long, curved, spine-like setae; length of setae 0.28 × maximum width of femur. Apex rounded, with a pair of long, curved, spine-like setae and one or two short, stout setae and some fine, simple setae. Many stout, lanceolate setae scattered along ventral margin; femoral patch absent. ***Tibia*.** Dorsal margin with a partial row of short, spine-like setae and a row of fine, simple setae, on apex one longer, spine-like seta. Ventral margin with a row of short, curved, spine-like setae, on apex one longer, bipectinate, spine-like seta and a tuft of fine, simple setae. Anterior surface scattered with stout, lanceolate setae. Patellotibial suture present on basal 1/3. ***Tarsus*.** Dorsal margin with some fine, simple setae. Ventral margin with a row of curved, spine-like setae. Claw with one row of 11–13 denticles; distally pointed; with ca. three stripes; subapical setae absent.

***Terga*** (Fig. 32c). Surface with irregular rows of U-shaped scale bases and scattered fine, simple setae and micropores. Posterior margin of tergum IV with triangular spines, wider than long.

***Gills*** (Fig. 32d). Present on segments II–VII. Margin with small denticles intercalating fine simple setae. Tracheae partly extending from main trunk towards outer and inner margins. Gill IV as long as length of segments V and 1/3 VI combined. Gill VII little longer than length of segment VIII.

***Paraproct*** (Fig. 32e). Distally expanded, with 36–39 stout, marginal spines. Surface scattered with U-shaped scale bases and micropores. Cercotractor with numerous small, marginal spines.

##### Etymology.

Dedicated to Mr. Emmanuel Delocado (Philippines), entomologist and member of the AdMU Biodiversity Laboratory.

##### Distribution.

Philippines: Cebu and Leyte (Fig. 49b).

##### Biological aspects.

The specimens were collected at altitudes of 50 m and 700 m, partly on rock surface in riffles or runs.

##### Type material.

***Holotype*.** Philippines • larva; Cebu, Cantipla; 10°24'55"N, 123°49'05"E; 750 m; 30.X.1995; leg. Mendoza; on slide; GenBank: MT830976; GBIFCH 00654886; PNM. ***Paratypes*.** Philippines • 2 larvae; same data as holotype; 1 on slide; GBIFCH 00592307; ZSM; 1 in alcohol; GBIFCH 00515451; AdMU • 86 larvae; Leyte, Southern Leyte, Brgy. Malico, San Francisco, Taglibas River; 10°01'07"N, 125°12'35'E; 50 m; 19–20.X.2019; leg. Garces and Pelingen; 1 on slide; MT830977; GBIFCH 00763668; AdMU; 6 in alcohol; GBIFCH 00515467; ZSM; 79 in alcohol; GBIFCH 00515494; AdMU.

#### Labiobaetis freitagi sp. nov.

Taxon classificationAnimaliaEphemeropteraBaetidae

792E44C2-9537-525D-AB81-FA3519E6CDC0

http://zoobank.org/2BC2CBB7-4E28-423E-B02D-6CB35711BDCC

**Figure 33.**
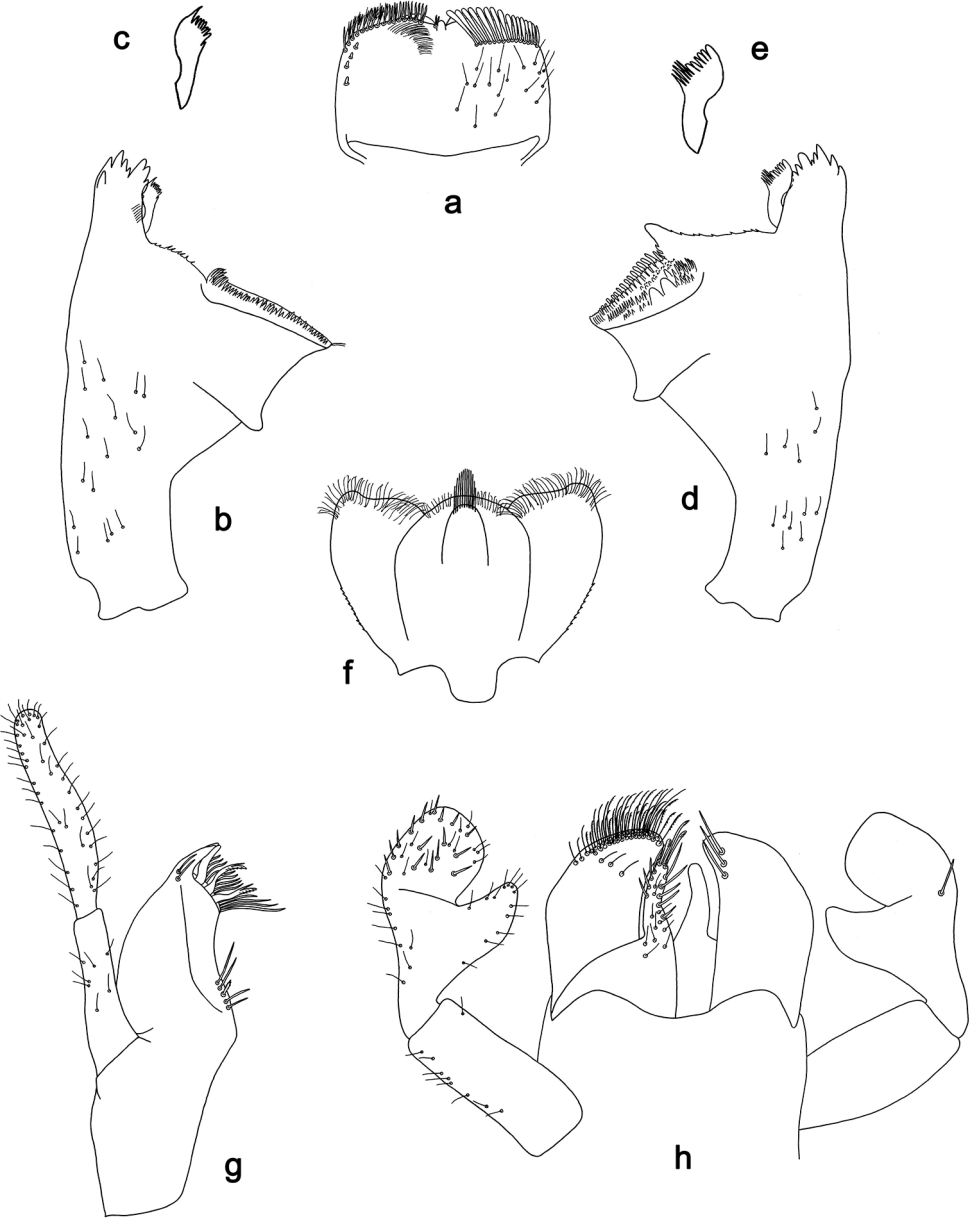
*Labiobaetis freitagi* sp. nov., larva morphology **a** labrum **b** right mandible **c** right prostheca **d** left mandible **e** left prostheca **f** hypopharynx and superlinguae **g** maxilla **h** labium.

**Figure 34.**
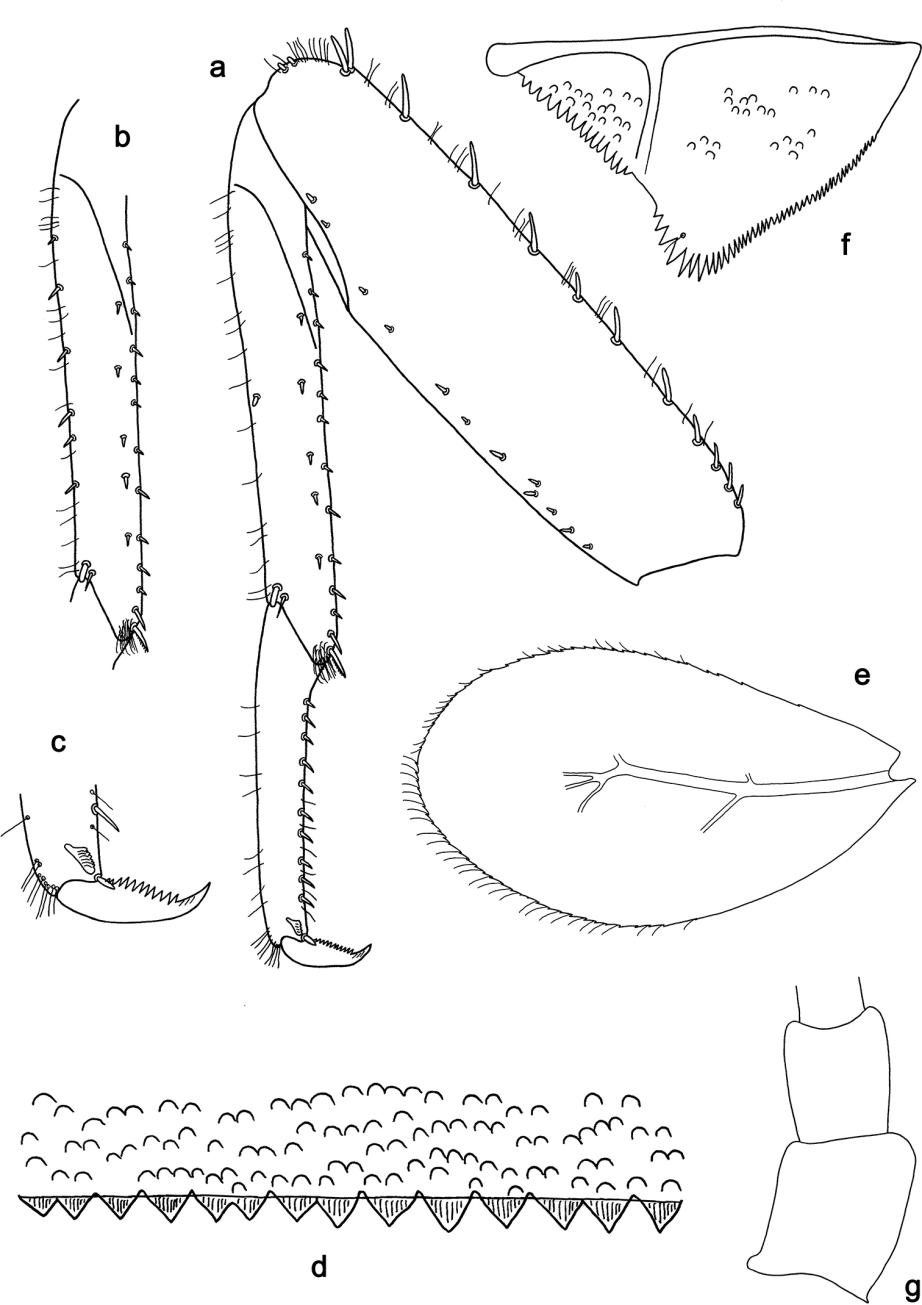
*Labiobaetis freitagi* sp. nov., larva morphology **a** foreleg **b** fore tibia **c** fore claw **d** tergum IV **e** gill IV **f** paraproct **g** antennal scape.

Figures 33 , 34 , 45a , 49b

##### Diagnosis.

**Larva.** Following combination of characters: A) dorsal surface of labrum with submarginal arc of ca. 17 long, clavate setae; B) labial palp segment II with a thumb-like distomedial protuberance, segment III sub-rectangular; C) left mandible without setae at apex of mola; D) fore femur rather slender, length 3.6 × maximum width, dorsal margin with ca. ten curved, spine-like setae; E) paraproct distally expanded, with 39–46 stout, marginal spines.

##### Description.

**Larva** (Figs 33, 34, 45a). Body length 3.1–4.8 mm. Cerci ca. 2/3 of body length. Paracercus ca. 2/3 of cerci length. Antenna approx. twice as long as head length.

***Colouration*.** Head, thorax, and abdomen dorsally light brown, fore protoptera light brown with bright striation. Head, thorax, and abdomen ventrally light brown, genae brown. Legs light brown, femur with a distomedial brown spot, dorsal and ventral margin and apex brown. Caudalii light brown.

***Antenna*** (Fig. 34g) with scape and pedicel subcylindrical, without distolateral process at scape.

***Labrum*** (Fig. 33a). Rectangular, length 0.7 × maximum width. Distal margin with medial emargination and a small process. Dorsally with medium to long, fine, simple setae scattered over surface; submarginal arc of setae composed of 16 or17 long, clavate setae. Ventrally with marginal row of setae composed of anterolateral long, feathered setae and medial long, bifid setae; ventral surface with ca. four short, spine-like setae near lateral and anterolateral margin.

***Right mandible*** (Fig. 33b, c). Incisor and kinetodontium fused. Incisor with five denticles; kinetodontium with three denticles, inner margin of innermost denticle with a row of thin setae. Prostheca robust, apically denticulate. Margin between prostheca and mola slightly convex, with minute denticles. Tuft of setae at apex of mola present.

***Left mandible*** (Fig. 33d, e). Incisor and kinetodontium fused. Incisor with five denticles; kinetodontium with three denticles. Prostheca robust, apically with small denticles and comb-shaped structure. Margin between prostheca and mola straight, with minute denticles towards subtriangular process. Subtriangular process long and slender, above level of area between prostheca and mola. Denticles of mola apically constricted. Tuft of setae at apex of mola absent.

Both mandibles with lateral margins almost straight. Basal half with fine, simple setae scattered over dorsal surface.

***Hypopharynx and superlinguae*** (Fig. 33f). Lingua shorter than superlinguae. Lingua longer than broad; medial tuft of stout setae well developed; distal half laterally expanded. Superlinguae distally slightly concave; lateral margin rounded; fine, long, simple setae along distal margin.

***Maxilla*** (Fig. 33g). Galea-lacinia ventrally with two simple, apical setae under canines. Inner dorsal row of setae with three denti-setae, distal denti-seta tooth-like, middle and proximal denti-setae slender, bifid and pectinate. Medially with one bipectinate, spine-like seta and four medium to long, simple setae. Maxillary palp 1.7 × as long as length of galea-lacinia; 2-segmented; palp segment II 1.5 × length of segment I; setae on maxillary palp fine, simple, scattered over surface of segments I and II; apex of last segment rounded, with slight excavation at inner distolateral margin.

***Labium*** (Fig. 33h). Glossa basally broad, narrowing toward apex; shorter than paraglossa; inner margin with ca. five spine-like setae increasing in length distally; apex with two long and one medium, robust, pectinate setae; outer margin with four spine-like setae; ventral surface with fine, simple, scattered setae. Paraglossa sub-rectangular, curved inward; apex rounded; with three rows of long, robust, distally pectinate setae in apical area and a row of three or four medium, simple setae in anteromedial area; dorsally with a row of three long, spine-like setae near inner margin. Labial palp with segment I 0.8 × length of segments II and III combined. Segment I ventrally with short, fine, simple setae. Segment II with thumb-like distomedial protuberance; distomedial protuberance 0.8 × width of base of segment III; ventral surface with short, fine, simple setae; dorsally with one long, spine-like seta near outer margin. Segment III subrectangular; apex rounded; length 1.0 × width; ventrally covered with short, spine-like, simple setae and short, fine, simple setae.

***Hind protoptera*** absent.

***Foreleg*** (Fig. 34a–c). Ratio of foreleg segments 1.2:1.0:0.6:0.2. ***Femur*.** Length ca. 4 × maximum width. Dorsal margin with ten or eleven long, curved, spine-like setae; length of setae 0.25 × maximum width of femur. Apex rounded, with a pair of long, curved, spine-like setae and some short stout and fine, simple setae. Many stout, lanceolate setae scattered along ventral margin; femoral patch absent. ***Tibia*.** Dorsal margin sometimes with a row of spine-like setae and always with a row of fine, simple setae, on apex two longer, spine-like setae. Ventral margin with a row of short, curved, spine-like setae, on apex some longer, partly bipectinate setae and a tuft of fine, simple setae. Anterior surface scattered with stout, lanceolate setae. Patellotibial suture present on basal 1/3. ***Tarsus*.** Dorsal margin with a row of fine, simple setae. Ventral margin with a row of curved, spine-like setae. Claw with one row of ten or eleven denticles; distally pointed; with ca. three stripes; subapical setae absent.

***Terga*** (Fig. 34d). Surface with irregular rows of U-shaped scale bases. Posterior margin of tergum IV with triangular spines, wider than long.

***Gills*** (Fig. 34e). Present on segments II–VII. Margin with small denticles intercalating fine simple setae. Tracheae partly extending from main trunk to inner and outer margins, pigmentation mainly limited to main trunk. Gill IV as long as length of segments V and 1/3 VI combined. Gill VII as long as length of segment VIII.

***Paraproct*** (Fig. 34f). Distally expanded with 39–46 stout, marginal spines. Surface scattered with U-shaped scale bases. Cercotractor with numerous small, marginal spines.

##### Etymology.

Dedicated to Prof. Hendrik Freitag (Philippines/Germany), collector of some material, for his outstanding contribution to freshwater biodiversity research in Southeast Asia.

##### Distribution.

Philippines: Palawan (Fig. 49b).

##### Biological aspects.

The specimens were collected at altitudes from sea level to 150 m, in leaf packs or submerged wood in runs or riffles.

##### Type material.

***Holotype*.** Philippines • larva; Palawan, Brooke’s Point, Mainit 7 Falls; 08°51'48"N, 117°47'45"E; 150 m; 2019; leg. Pelingen and Pangantihon; on slide; GBIFCH 00592322; PNM. ***Paratypes*.** Philippines • 22 larvae; same data as holotype; in alcohol; GenBank: MT830978; GBIFCH 00515497, GBIFCH 00763677; AdMU • 7 larvae; Palawan, P.Princesa Cabayugan District, Cabayugan River, near Nagsatayan Creek, S of Martarpi; 10°09'47"N, 118°50'37"E; 37 m; 05.VIII.2000; leg. Freitag; 1 on slide; GenBank: MT830978; GBIFCH 00763678; ZSM, temporarily stored in AdMU; 6 in alcohol; GBIFCH 00515498; PNM • 11 larvae; Palawan, Quezon, Isugod, Aramaywan River; 09°21'07"N, 118°08'26"E; 14 m; 2019; leg. Pangantihon and Pelingen; 1 on slide; GBIFCH 00592332; AdMU; 1 on slide; GBIFCH 00592333; ZSM, temporarily stored in AdMU; 6 in alcohol; GBIFCH 00515496; AdMU; 3 in alcohol; GenBank: MT830980; GBIFCH 00763682, GBIFCH 00515456; ZSM, temporarily stored in AdMU • 10 larvae; Palawan, Balabac, Suray River, Indalawan, near the road; 07°57'01"N, 117°04'29"E; 16 m; 2019; leg. Pangantihon and Pelingen; in alcohol; GenBank: MT830981; GBIFCH 00763683, GBIFCH 00515499; PCSD.

#### Labiobaetis pelingeni sp. nov.

Taxon classificationAnimaliaEphemeropteraBaetidae

DB80E3F6-108E-5014-B9E1-41F18917F852

http://zoobank.org/613FE6FB-4601-4A01-A07E-7FDF97AF55B1

**Figure 35.**
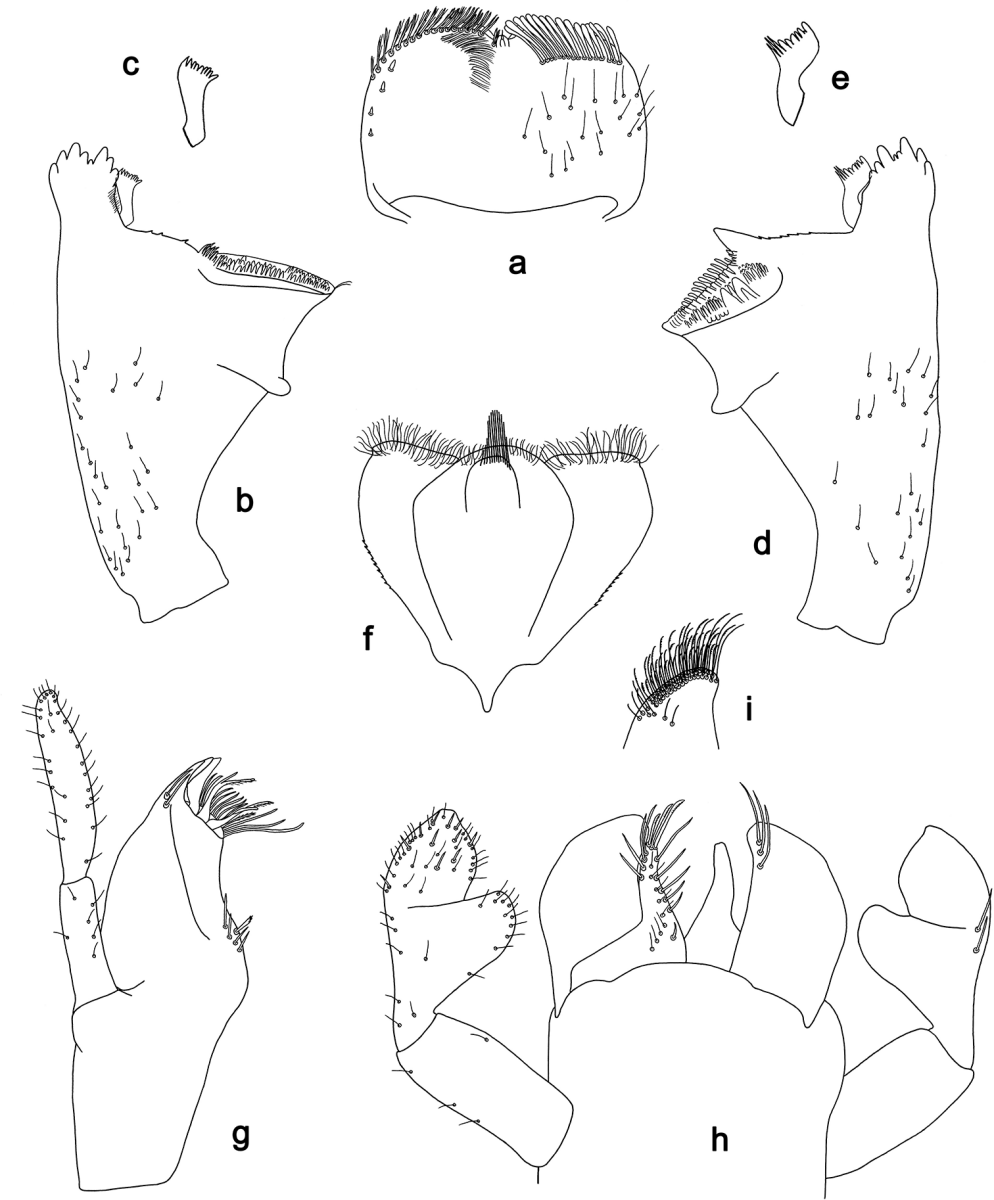
*Labiobaetis pelingeni* sp. nov., larva morphology **a** labrum **b** right mandible **c** right prostheca **d** left mandible **e** left prostheca **f** hypopharynx and superlinguae **g** maxilla **h** labium **i** apex of paraglossa.

**Figure 36.**
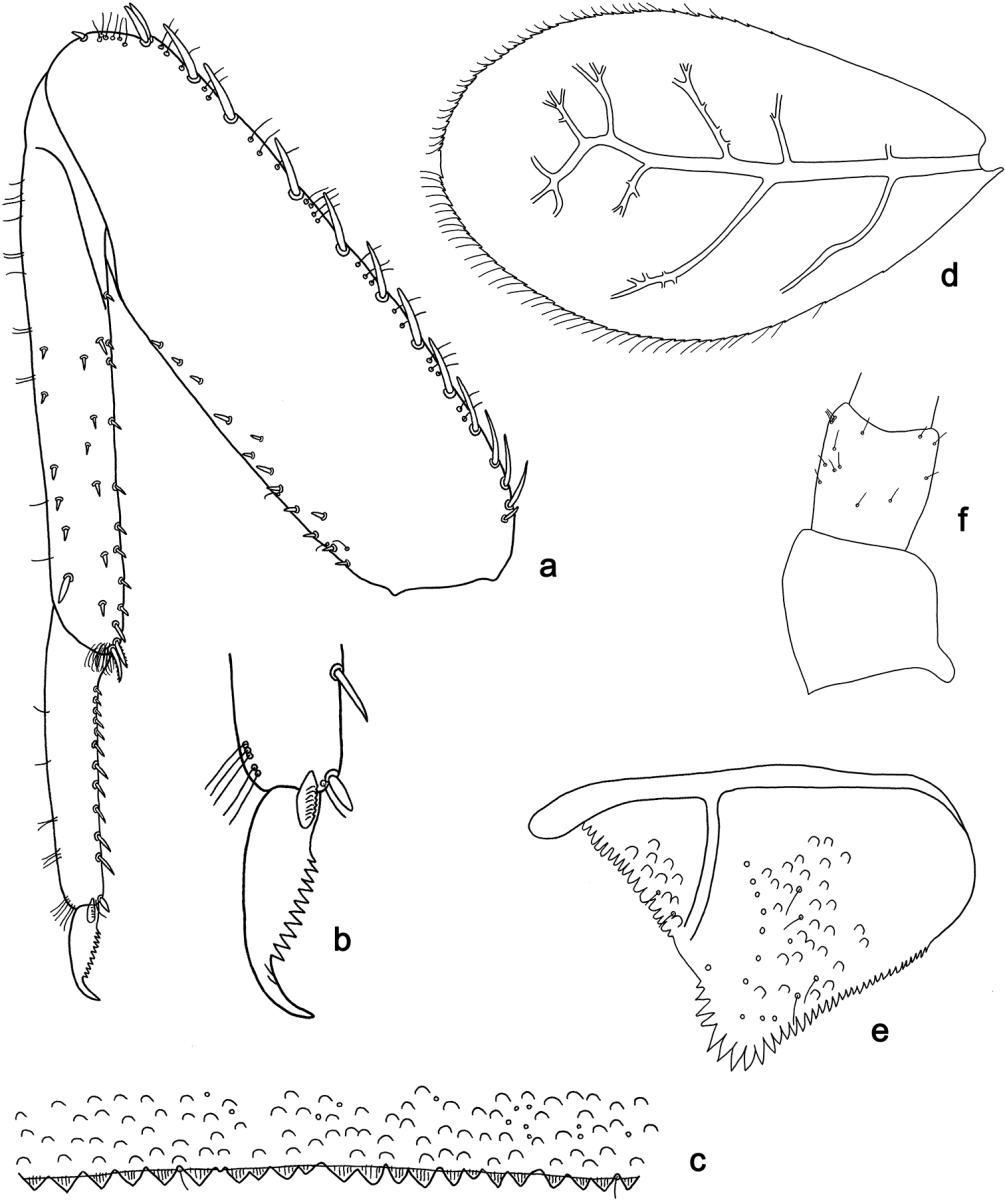
*Labiobaetis pelingeni* sp. nov., larva morphology **a** foreleg **b** fore claw **c** tergum IV **d** gill IV **e** paraproct **f** antennal scape.

Figures 35 , 36 , 45b , 49b

##### Diagnosis.

**Larva.** Following combination of characters: A) dorsal surface of labrum with submarginal arc of 15–17 long, clavate setae; B) labial palp segment II with a thumb-like distomedial protuberance, segment III slightly pentagonal; C) left mandible without setae at apex of mola; D) fore femur rather broad, length ca. 3 × maximum width, dorsal margin with 11–14 curved, spine-like setae; E) paraproct distally expanded, with ca. 35 stout, marginal spines.

##### Description.

**Larva** (Figs 35, 36, 45b). Body length 3.5–4.2 mm. Cerci ca. 1/2 of body length. Paracercus ca. 2/3 of cerci length. Antenna approx. twice as long as head length.

***Colouration*.** Head, thorax, and abdomen dorsally light brown, fore protoptera light brown. Head, thorax, and abdomen ventrally light brown. Legs light brown, femur with distomedial brown spot and brown apex. Caudalii light brown.

***Antenna*** (Fig. 36f) with scape and pedicel subcylindrical, without distolateral process at scape.

***Labrum*** (Fig. 35a). Rectangular, length 0.7 × maximum width. Distal margin with medial emargination and a small process. Dorsally with medium to long, fine, simple setae scattered over surface; submarginal arc of setae composed of 15–17 long, clavate setae. Ventrally with marginal row of setae composed of anterolateral long, feathered setae and medial long, bifid setae; ventral surface with ca. four short, spine-like setae near lateral and anterolateral margin.

***Right mandible*** (Fig. 35b, c). Incisor and kinetodontium fused. Incisor with five denticles; kinetodontium with three denticles, inner margin of innermost denticle with a row of thin setae. Prostheca robust, apically denticulate. Margin between prostheca and mola slightly convex, with minute denticles. Tuft of setae at apex of mola present.

***Left mandible*** (Fig. 35d, e). Incisor and kinetodontium fused. Incisor with five denticles; kinetodontium with three denticles. Prostheca robust, apically with small denticles and comb-shaped structure. Margin between prostheca and mola straight, with minute denticles towards subtriangular process. Subtriangular process long and slender, above level of area between prostheca and mola. Denticles of mola apically constricted. Tuft of setae at apex of mola absent.

Both mandibles with lateral margins almost straight. Basal half with fine, simple setae scattered over dorsal surface.

***Hypopharynx and superlinguae*** (Fig. 35f). Lingua approx. as long as superlinguae. Lingua longer than broad; medial tuft of stout setae well developed; distal half laterally expanded. Superlinguae distally straight; lateral margin rounded; fine, long, simple setae along distal margin.

***Maxilla*** (Fig. 35g). Galea-lacinia ventrally with two simple, apical setae under canines. Inner dorsal row of setae with three denti-setae, distal denti-seta tooth-like, middle and proximal denti-setae slender, bifid and pectinate. Medially with one bipectinate, spine-like seta and three or four medium to long, simple setae. Maxillary palp 1.3 × as long as length of galea-lacinia; 2-segmented; palp segment II 1.6 × length of segment I; setae on maxillary palp fine, simple, scattered over surface of segments I and II; apex of last segment rounded, with slight excavation at inner distolateral margin.

***Labium*** (Fig. 35h, i). Glossa basally broad, narrowing toward apex; shorter than paraglossa; inner margin with six spine-like setae increasing in length distally; apex with two long and one medium, robust, pectinate setae; outer margin with four spine-like setae increasing in length distally; ventral surface with fine, simple, scattered setae. Paraglossa sub-rectangular, curved inward; apex rounded; with three rows of long, robust, distally pectinate setae in apical area and two medium, simple setae in anteromedial area; dorsally with a row of three long, spine-like setae near inner margin. Labial palp with segment I 0.8 × length of segments II and III combined. Segment I ventrally with short, fine, simple setae. Segment II with thumb-like distomedial protuberance; distomedial protuberance 0.6 × width of base of segment III; ventral surface with short, fine, simple setae; dorsally with two long, spine-like, simple setae near outer margin. Segment III slightly pentagonal; apex slightly truncate; length 1.2 × width; ventrally covered with short, spine-like, simple setae and short, fine, simple setae.

***Hind protoptera*** absent.

***Foreleg*** (Fig. 36a, b). Ratio of foreleg segments 1.2:1.0:0.5:0.2. ***Femur*.** Length ca. 3 × maximum width. Dorsal margin with 11–14 long, curved, spine-like setae; length of setae 0.27 × maximum width of femur. Apex rounded; with a pair of long, curved, spine-like setae and one or two short, stout setae and some fine, simple setae. Many stout, lanceolate setae scattered along ventral margin; femoral patch absent. ***Tibia*.** Dorsal margin with a row of short, spine-like setae and fine simple setae, on apex one longer, spine-like seta. Ventral margin with a row of short, curved, spine-like setae, on apex some longer, partly bipectinate setae and a tuft of fine, simple setae. Anterior surface scattered with stout, lanceolate setae. Patellotibial suture present on basal 1/3. ***Tarsus*.** Dorsal margin with a row of fine, simple setae. Ventral margin with a row of curved, spine-like setae. Claw with one row of nine or ten denticles; distally pointed; with ca. two stripes; subapical setae absent.

***Terga*** (Fig. 36c). Surface with irregular rows of U-shaped scale bases and scattered micropores. Posterior margin of tergum IV with triangular spines, wider than long.

***Gills*** (Fig. 36d). Present on segments II–VII. Margin with small denticles intercalating fine simple setae. Tracheae extending from main trunk to inner and outer margins. Gill IV as long as length of segments V and 1/3 VI combined. Gill VII as long as length of segment VIII.

***Paraproct*** (Fig. 36e). Distally expanded, with ca. 35 stout, marginal spines. Surface scattered with U-shaped scale bases, fine, simple setae and micropores. Cercotractor with numerous small, marginal spines.

##### Etymology.

Dedicated to Mr. Arthien Lovell Pelingen (Philippines), entomologist and former project assistant of the AdMU Biodiversity Laboratory.

##### Distribution.

Philippines: Negros and Cebu (Fig. 49b).

##### Biological aspects.

The specimens were collected at altitudes of 50 m and 480 m, on submerged wood or in riverside pools.

##### Type material.

***Holotype*.** Philippines • larva; Negros Oriental, Valencia, Malaunay, small tributary; 09°18'17"N, 123°10'07"E; 480 m; 01.IX.2019; leg. Garces and Pelingen; on slide; GBIFCH 00592315; PNM. ***Paratypes*.** Philippines • 11 larvae; same data as holotype; 2 on slides; GenBank: MT830982; GBIFCH 00654901, GBIFCH 00592305; ZSM; 7 in alcohol; GBIFCH 00515490, GBIFCH 515491; AdMU; 2 in alcohol; GBIFCH 00515463; ZSM • 9 larvae; Cebu, Sogod, Bagatayam; 10°45'32"N, 123°59'49"E; 50 m.; 08.IX.1996; leg. Mendoza; 1 on slide; GenBank: MT830983; GBIFCH 00763672; AdMU; 5 in alcohol; GBIFCH 00515492; AdMU; 3 in alcohol; GBIFCH 515493, GBIFCH 00515462; ZSM.

### *Labiobaetis vallus* group of species (new group of species)

Following combination of characters: A) dorsal surface of labrum with submarginal arc of lanceolate setae; B) labial palp segment II with rather small, slender, thumb-like protuberance, distally bent upwards; C) hypopharynx with medial tuft of stout setae consisting of setae with minute apical serration; D) six pairs of gills; E) hind protoptera absent; F) no distolateral process at scape; G) paracercus short, ca. ¼ of cerci length.

#### Labiobaetis giselae sp. nov.

Taxon classificationAnimaliaEphemeropteraBaetidae

51FB48EA-7B5A-5CD5-818E-A16654A030E4

http://zoobank.org/9F0DC137-4C52-4B1C-A2DD-E2C40A8588DE

**Figure 37.**
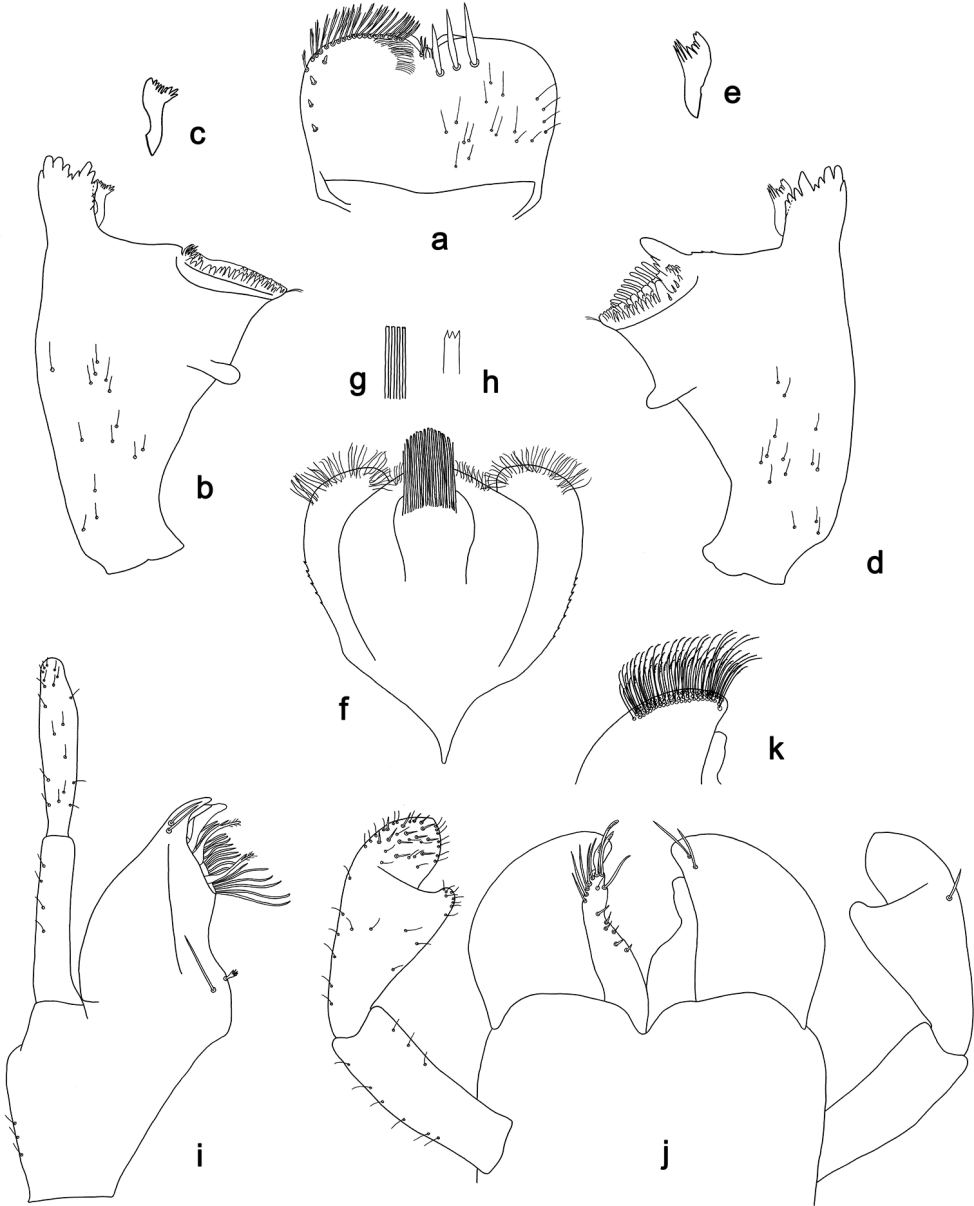
*Labiobaetis giselae* sp. nov., larva morphology **a** labrum **b** right mandible **c** right prostheca **d** left mandible **e** left prostheca **f** hypopharynx and superlinguae **g** hypopharynx, detail of tuft of stout setae **h** hypopharynx, seta of tuft of stout setae **i** maxilla **j** labium **k** apex of paraglossa.

**Figure 38.**
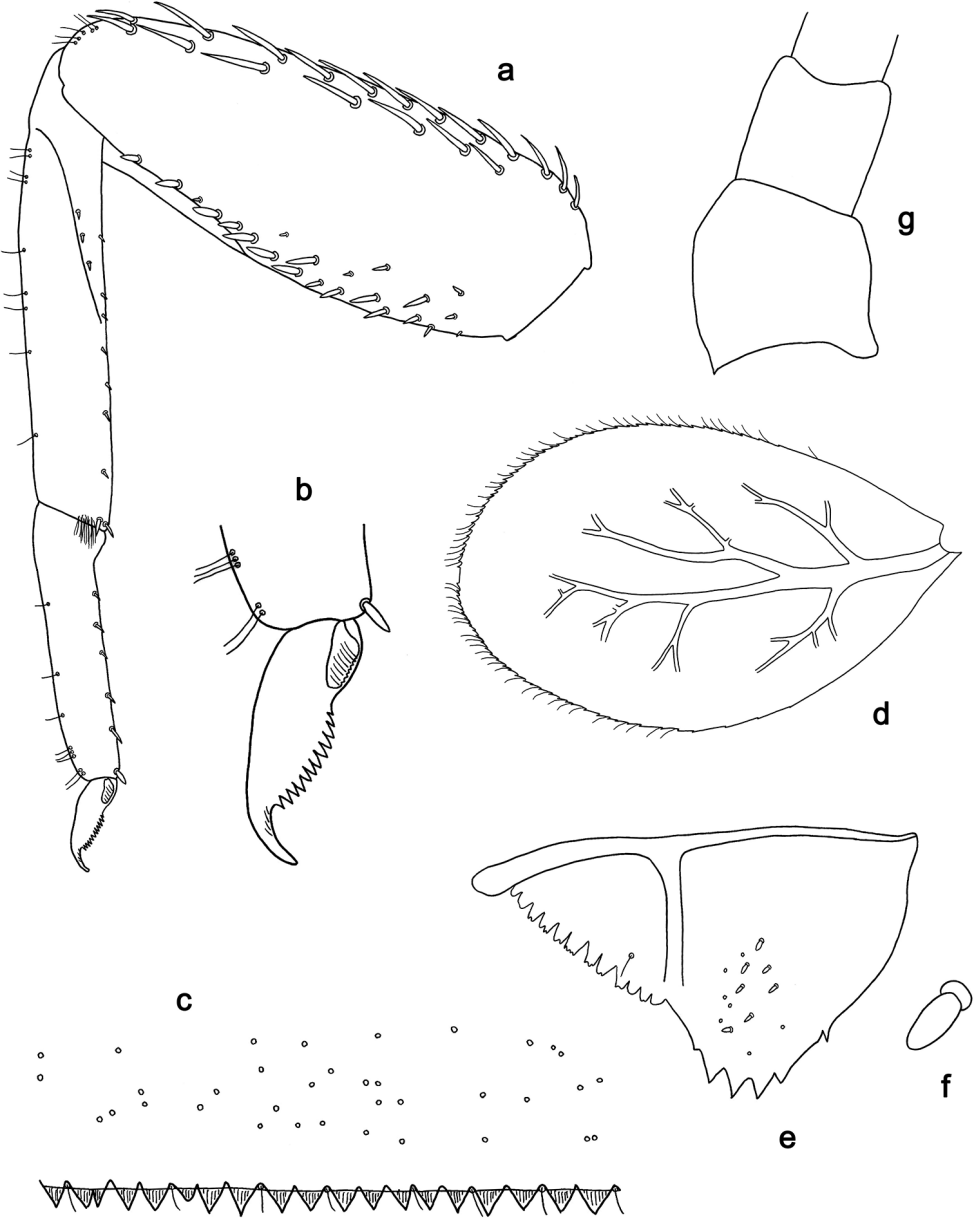
*Labiobaetis giselae* sp. nov., larva morphology **a** foreleg **b** fore claw **c** tergum IV **d** gill IV **e** paraproct **f** seta of paraproct **g** antennal scape.

Figures 37 , 38 , 45c , 49c

##### Diagnosis.

**Larva.** Following combination of characters: A) dorsal surface of labrum with submarginal arc of three long, lanceolate setae; B) labial palp segment II with a hook-like distomedial protuberance, segment III conical; C) hypopharynx with well-developed medial tuft of long, stout setae, setae apically with minute serration; D) fore femur rather broad, length 2.6 × maximum width, dorsal margin with ca. 12 long, curved, spine-like setae and a second row of spine-like setae near margin; E) paraproct distally expanded with ca. six stout, marginal spines; F) paracercus short, ca. ¼ of cerci length.

##### Description.

**Larva** (Figs 37, 38, 45c). Body length 3.4–4.5 mm. Cerci ca. 2/3 of body length. Paracercus ca. 1/4 of cerci length. Antenna approx. twice as long as head length.

***Colouration*.** Head and pronotum dorsally brown, meso- and metanotum light brown, fore protoptera light brown with brown apex. Abdominal segments II–VI dorsally dark brown, segments I and VII–X light brown, segments VII and X with brown markings as in Fig. 45c. Head and thorax ventrally light brown, abdominal segments II–VI ventrally dark brown, segments I and VII–X light brown, segment IX with brown anterior margin and segment X laterally darker. Femur ecru with a distomedial brown spot connected to brown streaks at dorsal and ventral margin and a basal brown area; tibia and tarsus light brown. Caudalii light brown.

***Antenna*** (Fig. 38g) with scape and pedicel subcylindrical, without distolateral process at scape.

***Labrum*** (Fig. 37a). Rectangular, length 0.6 × maximum width. Distal margin with medial emargination and a small process. Dorsally with medium, fine, simple setae scattered over surface; submarginal arc of setae composed of three long, lanceolate setae. Ventrally with marginal row of setae composed of anterolateral long, feathered setae and medial long, bifid setae; ventral surface with ca. four short, spine-like setae near lateral and anterolateral margin.

***Right mandible*** (Fig. 37b, c). Incisor and kinetodontium fused. Incisor with five denticles; kinetodontium with four denticles, inner margin of innermost denticle without a row of thin setae. Prostheca robust, apically denticulate. Margin between prostheca and mola straight. Tuft of setae at apex of mola present.

***Left mandible*** (Fig. 37d, e). Incisor and kinetodontium fused. Incisor with five denticles; kinetodontium with four denticles. Prostheca robust, apically with small denticles and comb-shaped structure. Margin between prostheca and mola straight, with minute denticles towards subtriangular process. Subtriangular process long and slender, above level of area between prostheca and mola. Denticles of mola apically constricted. Tuft of setae at apex of mola present.

Both mandibles with lateral margins slightly convex. Basal half with fine, simple setae scattered over dorsal surface.

***Hypopharynx and superlinguae*** (Fig. 37f–h). Lingua approx. as long as superlinguae. Lingua longer than broad; medial tuft of stout setae well developed, long, setae apically with minute serration; distal half laterally expanded. Superlinguae distally rounded; lateral margin rounded; fine, long, simple setae along distal margin.

***Maxilla*** (Fig. 37i). Galea-lacinia ventrally with two simple, apical setae under canines. Inner dorsal row of setae with three denti-setae, distal denti-seta tooth-like, middle and proximal denti-setae slender, bifid and pectinate. Medially with one spine-like, pectinate seta and one long, simple seta. Maxillary palp 1.6 × as long as length of galea-lacinia; 2-segmented; palp segment II 1.1 × length of segment I; setae on maxillary palp fine, simple, scattered over surface of segments I and II; apex of last segment rounded, with slight excavation at inner distolateral margin.

***Labium*** (Fig. 37j, k). Glossa basally broad, narrowing toward apex; shorter than paraglossa; inner margin with ca. four spine-like setae, distalmost seta much longer than other setae; apex with two long and one medium, robust, pectinate setae; outer margin with five long, spine-like setae; ventral surface with short, fine, simple and short, spine-like setae. Paraglossa sub-rectangular, curved inward; apex rounded; with three rows of long, robust, distally pectinate setae in apical area; dorsally with two or three long, spine-like setae near inner margin. Labial palp with segment I 0.9 × length of segments II and III combined. Segment I ventrally with short, fine, simple setae. Segment II with hook-like distomedial protuberance; distomedial protuberance 0.5 × width of base of segment III; ventral surface with short, fine, simple setae; dorsally with one long, spine-like seta near outer margin. Segment III conical; apex slightly pointed; length 1.0 × width; ventrally covered with short, spine-like, simple setae and short, fine, simple setae.

***Hind protoptera*** absent.

***Foreleg*** (Fig. 38a, b). Ratio of foreleg segments 1.2:1.0:0.6:0.2. ***Femur*.** Length ca. 3 × maximum width. Dorsal margin with ca. 12 long, curved, spine-like setae and a second row of long, spine-like setae near margin; length of setae 0.29 × maximum width of femur. Apex rounded, with a pair of curved, spine-like setae. Many stout, lanceolate setae scattered along ventral margin; femoral patch absent. ***Tibia*.** Dorsal margin with a row of fine, simple setae. Ventral margin with a row of short, curved, spine-like setae, on apex one longer, spine-like seta and a tuft of fine, simple setae. Anterior surface scattered with stout, lanceolate setae. Patellotibial suture present on basal half. ***Tarsus*.** Dorsal margin with a row of fine, simple setae. Ventral margin with a row of curved, spine-like setae. Claw with one row of 12 or 13 denticles; distally pointed; with ca. four stripes; subapical setae absent.

***Terga*** (Fig. 38c). Surface with scattered micropores. Posterior margin of tergum IV with triangular spines, approx. as long as wide.

***Gills*** (Fig. 38d). Present on segments II–VII. Margin with small denticles intercalating fine simple setae. Tracheae extending from main trunk to inner and outer margins. Gill IV as long as length of segments V and 1/3 VI combined. Gill VII as long as length of segments VIII and 1/3 IX combined.

***Paraproct*** (Fig. 38e, f). Distally expanded, with ca. six stout, marginal spines. Surface scattered with short, broad, lanceolate setae and micropores. Cercotractor with numerous broad, marginal spines, apically denticulate.

##### Etymology.

Dedicated to the late Mrs. Gisela Horzel (Germany), mother of one of the authors (TK).

##### Distribution.

Philippines: Luzon (Fig. 49c).

##### Biological aspects.

The specimens were collected at an altitude of 240 m on bottom gravel in runs or riffles.

##### Type material.

***Holotype*.** Philippines • larva; Luzon, Nueva Ecija, Pantabangan, Candaclan River; 15°46'48"N, 121°13'17"E; 240 m; 05.II.1998, leg. Mendoza; on slide; GBIFCH 00592280; PNM. ***Paratypes*.** Philippines • 4 larvae; same data as holotype; 2 on slides; GenBank: MT830984; GBIFCH 00654911, GBIFCH 00515469; ZSM; 2 in alcohol; GBIFCH 00515482; AdMU.

#### Labiobaetis mendozai sp. nov.

Taxon classificationAnimaliaEphemeropteraBaetidae

000DB263-B9CC-51A9-90E8-BFA10E5F5AE6

http://zoobank.org/58715368-B200-4AF6-87ED-38FB7CA90E58

**Figure 39.**
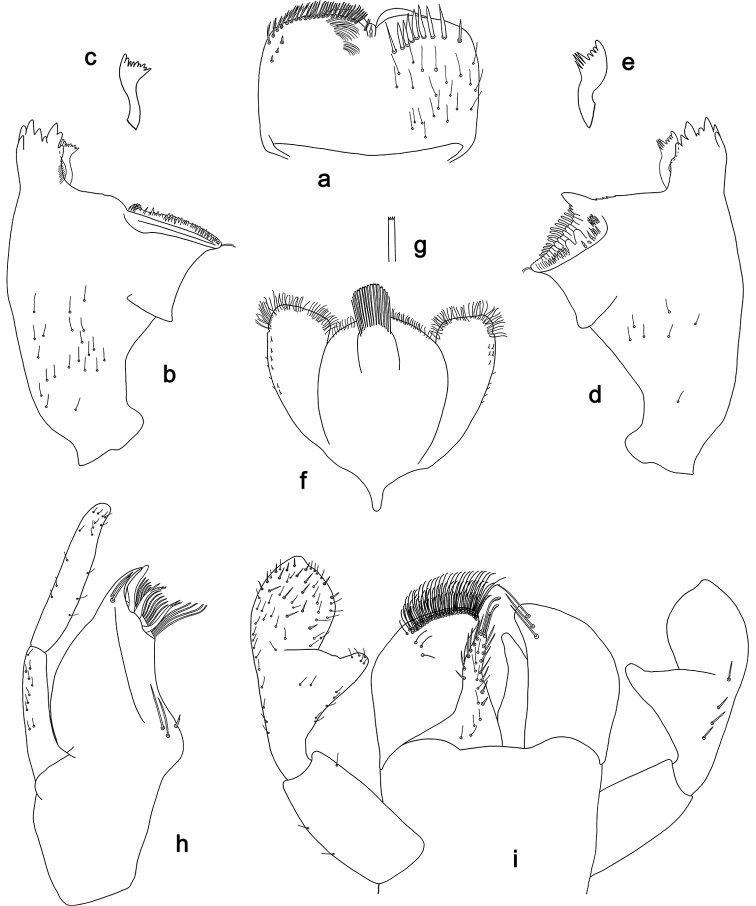
*Labiobaetis mendozai* sp. nov., larva morphology **a** labrum **b** right mandible **c** right prostheca **d** left mandible **e** left prostheca **f** hypopharynx and superlinguae **g** hypopharynx, seta of tuft of stout setae **h** maxilla **i** labium.

**Figure 40.**
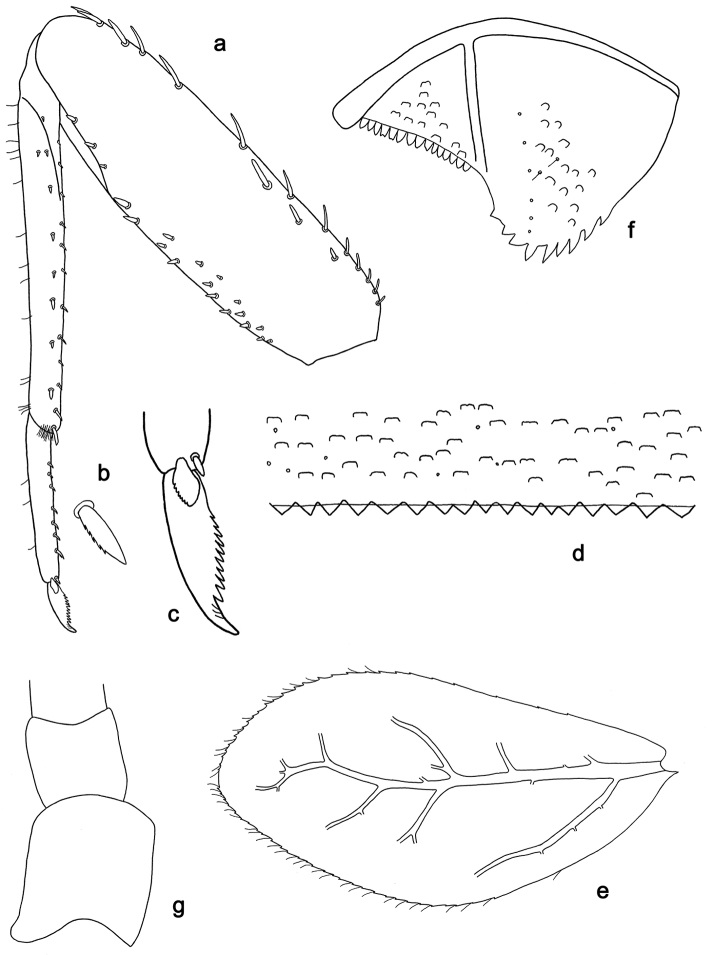
*Labiobaetis mendozai* sp. nov., larva morphology **a** foreleg **b** seta of ventral margin of tarsus **c** fore claw **d** tergum IV **e** gill IV **f** paraproct **g** antennal scape.

Figures 39 , 40 , 45d , 49c

##### Diagnosis.

**Larva.** Following combination of characters: A) dorsal surface of labrum with submarginal arc of one plus 5–8 long, lanceolate setae; B) labial palp segment II with a slender, thumb-like protuberance, segment III slightly pentagonal with small apical projection; C) hypopharynx with well-developed medial tuft of long, stout setae, setae apically with minute serration; D) fore femur length 3.4 × maximum width, dorsal margin with 8–11 curved, spine-like setae and at least a partial row of spine-like setae near margin; E) paraproct distally expanded, with ca. nine stout, marginal spines; F) paracercus short, ca. 1/4 of cerci length.

##### Description.

**Larva** (Figs 39, 40, 45d). Body length 3.2–4.6 mm. Cerci ca. 2/3 of body length. Paracercus ca. 1/4 of cerci length. Antenna approx. twice as long as head length.

***Colouration*.** Head, thorax, and abdomen dorsally brown with bright pattern as in Fig. 45d. Head, thorax, and abdomen light brown. Femur ecru with a distomedial brown spot connected to a brown streak at ventral margin and a light brown basal area. Tibia and tarsus light brown. Caudalii light brown.

***Antenna*** (Fig. 40g) with scape and pedicel subcylindrical, without distolateral process at scape.

***Labrum*** (Fig. 39a). Rectangular, length 0.7 × maximum width. Distal margin with medial emargination and a small process. Dorsally with medium to long, fine, simple setae scattered over surface; submarginal arc of setae composed of one plus 5–8 long, lanceolate setae, submedian seta and last seta of arc narrower. Ventrally with marginal row of setae composed of anterolateral long, feathered setae and medial long, bifid setae; ventral surface with ca. three short, spine-like setae near lateral and anterolateral margin.

***Right mandible*** (Fig. 39b, c). Incisor and kinetodontium fused. Incisor with five denticles; kinetodontium with three denticles, inner margin of innermost denticle with a row of thin setae. Prostheca robust, apically denticulate. Margin between prostheca and mola slightly convex. Tuft of setae at apex of mola present.

***Left mandible*** (Fig. 39d, e). Incisor and kinetodontium fused. Incisor with five denticles; kinetodontium with three denticles. Prostheca robust, apically with small denticles and comb-shaped structure. Margin between prostheca and mola straight, with minute denticles towards subtriangular process. Subtriangular process long and slender, above level of area between prostheca and mola. Denticles of mola apically constricted. Tuft of setae at apex of mola present.

Both mandibles with lateral margins slightly convex. Basal half with fine, simple setae scattered over dorsal surface.

***Hypopharynx and superlinguae*** (Fig. 39f, g). Lingua approx. as long as superlinguae. Lingua longer than broad; medial tuft of stout setae well developed, long, setae apically with minute serration; distal half laterally expanded. Superlinguae distally straight; lateral margin rounded; fine, long, simple setae along distal margin.

***Maxilla*** (Fig. 39h). Galea-lacinia ventrally with two simple, apical setae under canines. Inner dorsal row of setae with three denti-setae, distal denti-seta tooth-like, middle and proximal denti-setae slender, bifid and pectinate. Medially with one bipectinate, spine-like seta and two or three long, simple setae. Maxillary palp 1.4 × as long as length of galea-lacinia; 2-segmented; palp segment II 1.3 × length of segment I; setae on maxillary palp fine, simple, scattered over surface of segments I and II; apex of last segment rounded, with slight excavation at inner distolateral margin.

***Labium*** (Fig. 39i). Glossa basally broad, narrowing toward apex; shorter than paraglossa; inner margin with ca. seven spine-like setae increasing in length distally; apex with two long and one medium, robust, pectinate setae; outer margin with five or six spine-like setae increasing in length distally; ventral surface with fine, simple, scattered setae. Paraglossa sub-rectangular, curved inward; apex rounded; with three rows of long, robust, distally pectinate setae in apical area and three medium, simple setae in anteromedial area; dorsally with a row of three long, spine-like setae near inner margin. Labial palp with segment I 0.6 × length of segments II and III combined. Segment I ventrally with short, fine, simple setae. Segment II with slender, thumb-like, distomedial protuberance, distally bent upward; distomedial protuberance 0.8 × width of base of segment III; ventral surface with short, fine, simple setae; dorsally with a row of 2–4 medium, spine-like setae near outer margin. Segment III slightly pentagonal; apex with small projection; length 1.2 × width; ventrally covered with short, spine-like, simple setae and short, fine, simple setae.

***Hind protoptera*** absent.

***Foreleg*** (Fig. 40a–c). Ratio of foreleg segments 1.2:1.0:0.4:0.1. ***Femur*.** Length ca. 3 × maximum width. Dorsal margin with 8–11 curved, spine-like setae and at least a partial row of spine-like setae near margin; length of setae 0.27 × maximum width of femur. Apex rounded; with a pair of curved, spine-like setae. Many stout, lanceolate setae scattered along ventral margin; femoral patch absent. ***Tibia*.** Dorsal margin with a row of fine, simple setae. Ventral margin with a row of short, curved, spine-like setae, on apex one longer, spine-like seta and a tuft of fine, simple setae. Anterior surface scattered with stout, lanceolate setae. Patellotibial suture present on basal 1/3. ***Tarsus*.** Dorsal margin with a row of fine, simple setae. Ventral margin with a row of curved, spine-like setae with minute marginal pectination (pectination difficult to see). Claw with one row of ten denticles; distally pointed; with ca. three stripes; subapical setae absent.

***Terga*** (Fig. 40d). Surface with irregular rows of W-shaped scale bases and scattered micropores. Posterior margin of tergum IV with triangular spines, wider than long.

***Gills*** (Fig. 40e). Present on segments II–VII. Margin with small denticles intercalating fine simple setae. Tracheae extending from main trunk to inner and outer margins. Gill IV as long as length of segments V and half VI combined. Gill VII as long as length of segments VIII and 1/3 IX combined.

***Paraproct*** (Fig. 40f). Distally expanded, with ca. nine stout, marginal spines. Surface scattered with U-shaped scale bases and fine, simple setae. Cercotractor with numerous broad, marginal spines.

##### Etymology.

Dedicated to the late Mr. Joseph Mendoza (Philippines), outstanding insect collector.

##### Distribution.

Philippines: Mindanao (Fig. 49c).

##### Biological aspects.

The specimens were collected at altitudes from sea level to 120 m, on bottom gravel or submerged wood, or in leaf litter in side pools.

##### Type material.

***Holotype*.** Philippines • larva; Mindanao, Surigao del Sur, Tandag, Pangi, Pangi River; 09°06'18"N, 126°08'53"E; 10 m; 30.XI.2018, leg. Pangantihon; on slide; GBIFCH 00592272; PNM. ***Paratypes*.** Philippines • 5 larvae; same data as holotype; 1 in alcohol; GBIFCH 00515480; ZSM; 4 in alcohol; GBIFCH 00515481; AdMU • 2 larvae; Mindanao, Agusan del Sur, San Francisco, Bayogan, Tagkunayan Creek; 08°28'N, 125°59'E; 120 m; 05.II.1998; leg. Mendoza; 1 on slide; GBIFCH 00515476; AdMU; 1 on slide; GenBank: MT830985; GBIFCH 654894; ZSM • 1 larva; Mindanao, Surigao del Sur, Tandag, Buenavista River; 09°08'18"N, 126°08'45"E; 80 m; 03.XII.2018; leg. Panganthion; in alcohol; GBIFCH 00515478; ZSM.

### Key to the *Labiobaetis* species of the Philippines (larvae)^*^

**Table:** 

1	Dorsal surface of labrum with submarginal arc of simple setae (Fig. 2a)	**(numeratus group) 2**
–	Dorsal surface of labrum with submarginal arc of other types of setae	**7**
2	Setae at apex of mola of left mandible absent (Fig. 11d)	***L. lachicae* sp. nov.**
–	Setae at apex of mola of left mandible present	**3**
3	Hypopharynx with medial tuft of stout setae poorly developed (Fig. 2t); mola of right mandible proximally beginning with a double hump (Fig. 13b)	**4**
–	Hypopharynx with medial tuft of stout setae well developed, short (Fig. 2s); mola of right mandible proximally not beginning with a double hump	**5**
4	Posterior margin of tergum IV with triangular spines (Fig. 16c)	***L. sabordoi* sp. nov.**
–	Posterior margin of tergum IV with rounded spines (Fig. 14c)	***L. palawano* sp. nov.**
5	Left mandible with comb-shaped structure at base of mola (Fig. 5f, g)	**6**
–	Left mandible without comb-shaped structure at base of mola (Fig. 9d)	***L. camiguinensis* sp. nov.**
6	Paraproct with 18–21 marginal spines	***L. aldabae* sp. nov.**
–	Paraproct with 29–34 marginal spines plus a few submarginal spines	***L. acei* sp. nov.**
7	Dorsal surface of labrum with submarginal arc of clavate setae (Fig. 2c)	**(*sumigarensis* group) 8**
–	Dorsal surface of labrum with submarginal arc of other types of setae	**13**
8	Labial palp segment II enlargement pronounced hook-like with inner margin straight, segment III oblong and apically slightly truncate (Fig. 28c)	***L. sumigarensis***
–	Labial palp different	**9**
9	Labial palp segment II elongate thumb-like with distal margin strongly concave and inner margin concave (Figs 31i, 33h)	**10**
–	Labial palp segment II broad thumb-like with distal margin not or only slightly concave (Fig. 29i)	**11**
10	Labial palp segment III apically slightly pointed (Fig. 31i); maxillary palp longer than galea-lacinia (ca. 1.3×) (Fig. 31h)	***L. delocadoi* sp. nov.**
–	Labial palp segment III apically rounded (Fig. 33h); maxillary palp much longer than galea-lacinia (ca. 1.7×) (Fig. 33g)	***L. freitagi* sp. nov.**
11	Paraproct distally not expanded (Fig. 27c); distolateral process at scape poorly developed (Fig. 2g)	***L. molawinensis***
–	Paraproct distally expanded (Fig. 30e); distolateral process at scape absent (Fig. 2f)	**12**
12	Maxillary palp with distolateral excavation (Fig. 29h); dorsal surface of labrum with submarginal arc of 17–21 clavate setae (Fig. 29a)	***L. baganii* sp. nov.**
–	Maxillary palp with slight distolateral excavation (Fig. 35g); dorsal surface of labrum with submarginal arc of 15–17 clavate setae (Fig. 35a)	***L. pelingeni* sp. nov.**
13	Dorsal surface of labrum with submarginal arc of lanceolate setae (Fig. 2e)	**(*vallus* group) 14**
–	Dorsal surface of labrum with submarginal arc of other types of setae	**15**
14	Dorsal surface of labrum with submarginal arc of ca. three lanceolate setae; row of fine setae at innermost denticle of kinetodontium of right mandible absent (Fig. 37b)	***L. giselae* sp. nov.**
–	Dorsal surface of labrum with submarginal arc of one plus ca. eight lanceolate setae; row of fine setae at innermost denticle of kinetodontium of right mandible present (Fig. 39b)	***L. mendozai* sp. nov.**
15	Dorsal surface of labrum with submarginal arc of feathered setae (Fig. 2b), feathers may be reduced or strongly reduced; labial palp segment II enlargement thumb-like, segment III conical (Fig. 2j); femoral patch absent	**(*operosus* group) 16**
–	Dorsal surface of labrum with submarginal arc of dendritic setae (Fig. 2d); labial palp segment II enlargement narrow, elongate thumb-like, segment III apically rounded (Fig. 3j); femoral patch present (Fig. 4a)	**(*dendrisetis* group) *L. dalisay* sp. nov.**
16	Dorsal surface of labrum with submarginal arc of setae with reduced or strongly reduced feathers (Figs 17b, 21b)	**17**
–	Dorsal surface of labrum with submarginal arc of feathered setae, feathers not reduced (Fig. 19a)	**18**
17	Feathered setae of submarginal arc on dorsal surface of labrum strongly reduced, with few lateral branches only (Fig. 17b); hind protoptera well developed (Fig. 18g)	***L. gamay* sp. nov.**
–	Feathered setae of submarginal arc on dorsal surface of labrum moderately reduced (Fig. 21b); hind protoptera of medium size (Fig. 22g)	***L. tagbanwa* sp. nov.**
18	Labial palp segment II enlargement narrow thumb-like (Fig. 19h); row of fine setae at innermost denticle of kinetodontium of right mandible absent (Fig. 19b)	***L. pangantihoni* sp. nov.**
–	Labial palp segment II enlargement broad thumb-like (Fig. 23h); row of fine setae at innermost denticle of kinetodontium of right mandible present (Fig. 23b)	**19**
19	Fore tarsus dorsoventrally concave (broadened) (Fig. 24a); spines at posterior margin of tergum IV shorter than wide (Fig. 24c)	***L. valdezorum* sp. nov.**
–	Fore tarsus dorsoventrally parallel sided (Fig. 26a); spines at posterior margin of tergum IV mainly longer than wide (Fig. 26c)	***L. wantzeni* sp. nov.**

### Distribution

The material treated in this study was collected in many different locations across the Philippine archipelago, including most of the big islands as well as some smaller islands (Figs 48, 49). There are still many regions in the Philippines as well as in Southeast Asia where no sampling of mayflies has yet been done and many species known to date are from a single population only. This implies that the current diversity and distribution must be still considered very preliminary. In terms of altitude, the *Labiobaetis* species of the Philippines were found from sea level to mountain areas up to 1,820 m. The GPS coordinates of the locations of examined material are given in Table 2.

**Table 2.** GPS coordinates of locations of examined specimens.

Species	Species group	Locality	GPS coordinates
*L. dalisay* sp. nov.	*dendrisetis* gr.	Philippines: Luzon	15°45'48''N, 121°25'21''E
			15°46'48''N, 121°13'17''E
			16°21'33"N, 120°30'31"E
*L. acei* sp. nov.	*numeratus* gr.	Philippines: Luzon	17°03'53"N, 121°05'10"E
			16°59'37"N, 121°02'51"E
*L. aldabae* sp. nov.	*numeratus* gr.	Philippines: Luzon	14°08'N, 121°31'E
			15°46'48''N, 121°13'17''E
			14°32'47''N, 121°13'42''E
			16°57'17''N, 120°38'52' E
			16°34'11"N, 120°50'12"E
			16°21'33"N, 120°30'31"E
			16°40'58"N, 120°56'59"E
		Philippines: Negros	09°18'17''N, 123°10'07''E
*L. camiguinensis* sp. nov.	*numeratus* gr.	Philippines: Camiguin	09°06'39''N, 124°43'45''E
			09°09'25''N, 124°43'57''E
*L. lachicae* sp. nov.	*numeratus* gr.	Philippines: Mindanao	08°28'N, 125°59'E
			09°03'33''N, 126°05'57''E
			09°06'18''N, 126°08'53''E
*L. palawano* sp. nov.	*numeratus* gr.	Philippines: Busuanga	12°03'46''N, 120°13'25''E
			12°01'45''N, 120°12'19''E
		Philippines: Palawan	10°09'47''N, 118°50'37''E
			10°01'26''N, 119°04'37''E
*L. sabordoi* sp. nov.	*numeratus* gr.	Philippines: Negros	09°18'N, 123°14'E
		Philippines: Romblon	12°33'38''N, 122°07'19''E
			12°20'40''N, 122°40'37''E
*L. gamay* sp. nov.	*operosus* gr.	Philippines: Mindoro	12°37'06''N, 121°23'49''E
			12°37'18"N, 121°22'58"E
		Philippines: Luzon	16°54'38"N, 120°28'40"E
			16°59'32"N, 120°32'21"E
			16°39'27"N, 120°25'55"E
*L. pangantihoni* sp. nov.	*operosus* gr.	Philippines: Palawan	07°57'39''N, 117°02'59''E
			07°57'39''N, 117°02'59''E
			09°21'07''N, 118°08'26''E
			09°18'25''N, 118°07'42''E
*L. tagbanwa* sp. nov.	*operosus* gr.	Philippines: Palawan	09°41'20''N, 118°37'29''E
			09°26'55''N, 118°26'44''E
			09°22'33''N, 118°08'41''E
*L. valdezorum* sp. nov.	*operosus* gr.	Philippines: Negros	09°18'N, 123°14'E
			09°17'N, 123°13'E
		Philippines: Cebu	10°28'13"N, 123°52'26"E
*L. wantzeni* sp. nov.	*operosus* gr.	Philippines: Camiguin	09°06'39''N, 124°43'45''E
			09°12'N, 124°41'E
			09°09'25''N, 124°43'57''E
		Philippines: Mindanao	08°09'42''N, 124°42'28''E
*L. molawinensis*	*sumigarensis* gr.	Philippines: Luzon	14°09'53''N, 121°14'48''E
			14°10'05''N, 121°11'44''E
			14°08'N, 121°31'E
			15°45'21''N, 121°34'46''E
*L. sumigarensis*	*sumigarensis* gr.	Philippines: Luzon	16°59'37''N, 121°02'51''E
*L. baganii* sp. nov.	*sumigarensis* gr.	Philippines: Mindanao	09°10'15''N, 125°40'55''E
			09°20'40''N, 125°30'50''E
			08°28'N, 125°59'E
			09°11'34''N, 125°36'34''E
		Philippines: Camiguin	09°06'39''N, 124°43'45''E
*L. delocadoi* sp. nov.	*sumigarensis* gr.	Philippines: Cebu	10°24'55''N, 123°49'05''E
		Philippines: Leyte	10°01'07''N, 125°12'35'E
*L. freitagi* sp. nov.	*sumigarensis* gr.	Philippines: Palawan	08°51'48''N, 117°47'45''E
			10°09'47''N, 118°50'37''E
			09°21'07''N, 118°08'26''E
			07°57'01''N, 117°04'29''E
*L. pelingeni* sp. nov.	*sumigarensis* gr.	Philippines: Negros	09°18'17''N, 123°10'07''E
		Philippines: Cebu	10°45'32"N, 123°59'49"E
*L. giselae* sp. nov.	*vallus* gr.	Philippines: Luzon	15°46'48''N, 121°13'17''E
*L. mendozai* sp. nov.	*vallus* gr.	Philippines: Mindanao	09°08'18''N, 126°08'45''E
			08°28'N, 125°59'E
			09°06'18''N, 126°08'53''E

**Figure 41.**
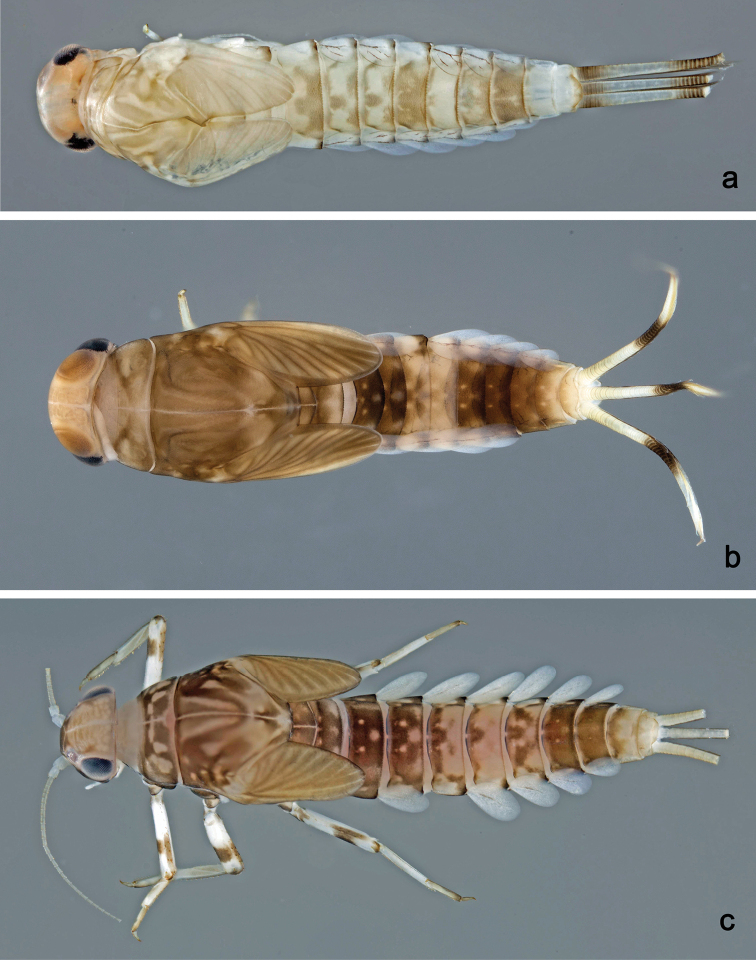
Habitus, larvae, dorsal view **a***Labiobaetis dalisay* sp. nov. **b***Labiobaetis acei* sp. nov. **c***Labiobaetis aldabae* sp. nov.

### Genetics

COI sequences were obtained from all 18 new species (Table 1). The genetic distances (K2P) of the species in the Philippines are between 15% and 27%, and therefore much higher than 3.5%, which is generally considered as a likely maximal value for intraspecific divergence (Hebert et al. 2003, Ball et al. 2005, Zhou et al. 2010) (Table 3). Very limited genetic distances (between 0% and 3%) were found between specimens of the same species, as in *L. acei* sp. nov., *L. palawano* sp. nov., *L. sabordoi* sp. nov., *L. gamay* sp. nov., *L. tagbanwa* sp. nov., *L. valdezorum* sp. nov., *L. wantzeni* sp. nov., *L. baganii* sp. nov., *L. delocadoi* sp. nov., *L. freitagi* sp. nov. and *L. pelingeni* sp. nov. An exception is *L. aldabae* sp. nov., where we found a genetic distance up to 5% between different locations on the same island (Luzon) and 6% between locations on different islands (Luzon and Negros).

**Table 3.** Intraspecific (bold) and interspecific genetic distances of the new species (COI; Kimura 2-parameter; %; mean; minimum-maximum).

		1	2	3	4	5	6	7	8	9	10	11	12	13	14	15	16	17	18
1	*L. dalisay* sp. nov.	**0**																	
2	*L. acei* sp. nov.	18	**0**																
3	*L. aldabae* sp. nov.	20	16	**3**															
		20–22	15–16	**0–6**															
4	*L. camiguinensis* sp. nov.	23	19	16	**0**														
5	*L. lachicae* sp. nov.	22	18	18	6	**0**													
6	*L. palawano* sp. nov.	22	17	18	15	16	**2**												
		21–22	16–17	17–18	14–15	15–16													
7	*L. sabordoi* sp. nov.	22	17	19	20	18	16	**1**											
		21–22		18–19		17–18	15–16												
8	*L. gamay* sp. nov.	25	21	20	20	21	20	22	**0**										
		24–25	21–22	19–21			19–20	22–23	**0–1**										
9	*L. pangantihoni* sp. nov.	22	15	17	17	18	17	17	20	**0**									
				16–17			16–18		20–21										
10	*L. tagbanwa* sp. nov.	22	20	17	19	19	20	19	13	20	**1**								
				17–18			19–20	18–19			**0–1**								
11	*L. valdezorum* sp. nov.	22	22	20	21	22	22	23	18	23	17	**0**							
				19–21			21–22	22–23	18–19		17–18								
12	*L. wantzeni* sp. nov.	24	19	18	21	23	20	21	21	19	21	20	**1**						
				17–19			19–21	20–21					**0–2**						
13	*L. baganii* sp. nov.	22	18	18	20	21	18	19	21	20	19	24	21	**2**					
				17–19	19–20	20–21			20–21	19–20	18–20								
14	*L. delocadoi* sp. nov.	24	24	23	23	23	21	23	23	22	23	24	23	24	**2**				
		23–24	23–24	21–25	22–23	22–23	20–21	22–24	22–24	21–22	22–23			23–24					
15	*L. freitagi* sp. nov.	26	25	22	23	23	23	23	26	23	26	27	26	22	23	**0**			
				21–22	22–23	23–24	23–24	22–23	26–27	22–23	25–26	26–27			22–24	**0–1**			
16	*L. pelingeni* sp. nov.	24	19	19	22	19	20	22	24	21	22	23	24	21	23	23	**3**		
		23–24	18–19	18–21	21–22		19–20	21–22	23–24		21–22	23–24	22–24	20–21	22–23	22–24			
17	*L. giselae* sp. nov.	21	18	18	21	20	23	20	22	19	20	20	17	21	21	23	22	**0**	
				17–19			22–23				19–20	20–21	17–18	20–21	20–21	22–23	21–22		
18	*L. mendozai* sp. nov.	23	21	19	20	20	19	22	22	23	19	20	24	22	24	27	21	21	**0**
							18–19				18–19	20–21	23–25		23–24	26–27			

**Figure 42.**
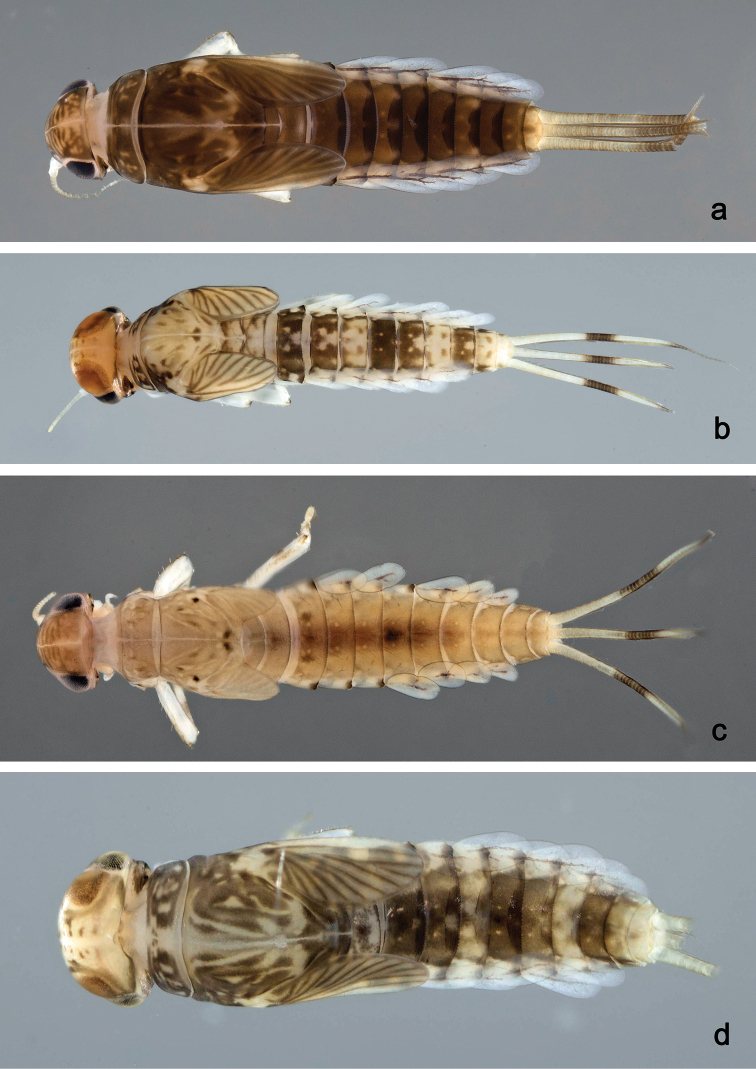
Habitus, larvae, dorsal view **a***Labiobaetis camiguinensis* sp. nov. **b***Labiobaetis lachicae* sp. nov. **c***Labiobaetis palawano* sp. nov. **d***Labiobaetis sabordoi* sp. nov.

## Discussion

### Assignment to *Labiobaetis*

For the assignment of the new species to *Labiobaetis* we refer to Kluge and Novikova (2014), Müller-Liebenau (1984) and McCafferty and Waltz (1995). *Labiobaetis* is characterized by a number of derived characters, some of which are not found in other taxa (Kluge and Novikova 2014): antennal scape sometimes with a distolateral process (Fig. 2h); maxillary palp two segmented with excavation at inner distolateral margin of segment II, excavation may be poorly developed or absent (Fig. 2n–p); labium with paraglossae widened and glossae diminished; labial palp segment II with distomedial protuberance (Fig. 2i–m). All these characters vary and may be secondarily lost (Kluge and Novikova 2014). The concept of *Labiobaetis* is also based on additional characters, summarized and discussed in Kaltenbach and Gattolliat (2018, 2019).

**Figure 43.**
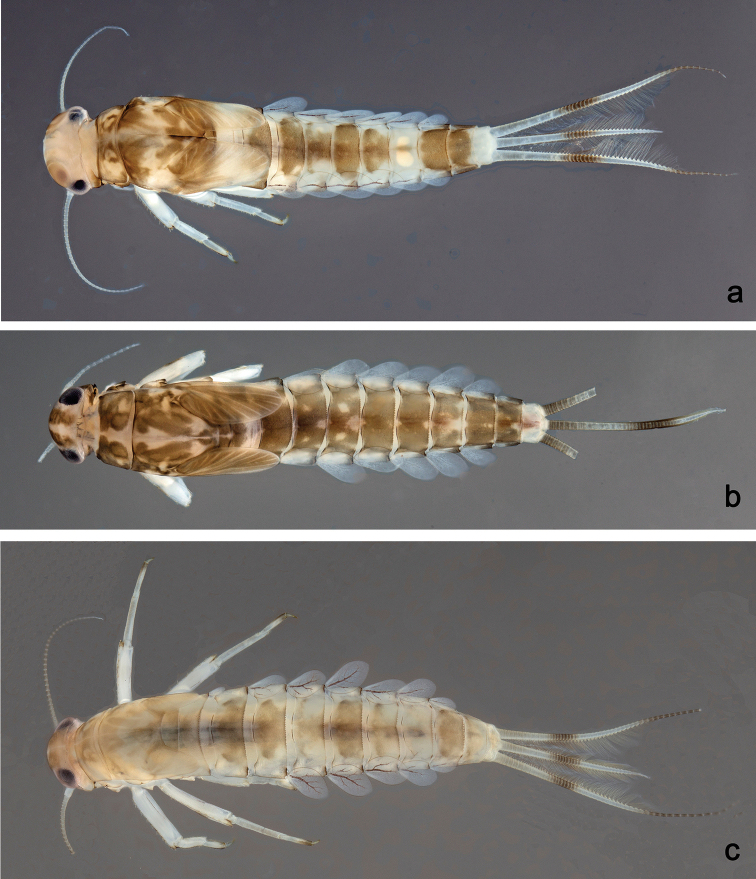
Habitus, larvae, dorsal view **a***Labiobaetis gamay* sp. nov. **b***Labiobaetis pangantihoni* sp. nov. **c***Labiobaetis tagbanwa* sp. nov.

From the 16 species of *Labiobaetis* (or previously assigned to *Pseudocloeon*) only known at the imaginal stage, one was described from the Philippines (Mindanao; *Pseudocloeonboettgeri Ulmer*, 1924). As the identification of the imaginal stage of *Labiobaetis* is generally very difficult, we consider it unrealistic to safely associate the larval stage with old type material at the imaginal stage. In this case, rearing material will provide little help. Furthermore, the generic assignment of the species remains questionable. Therefore, we did not take this species into account in our study and wait for an eventual clarification of its status in the future by using ancient DNA methods.

**Figure 44.**
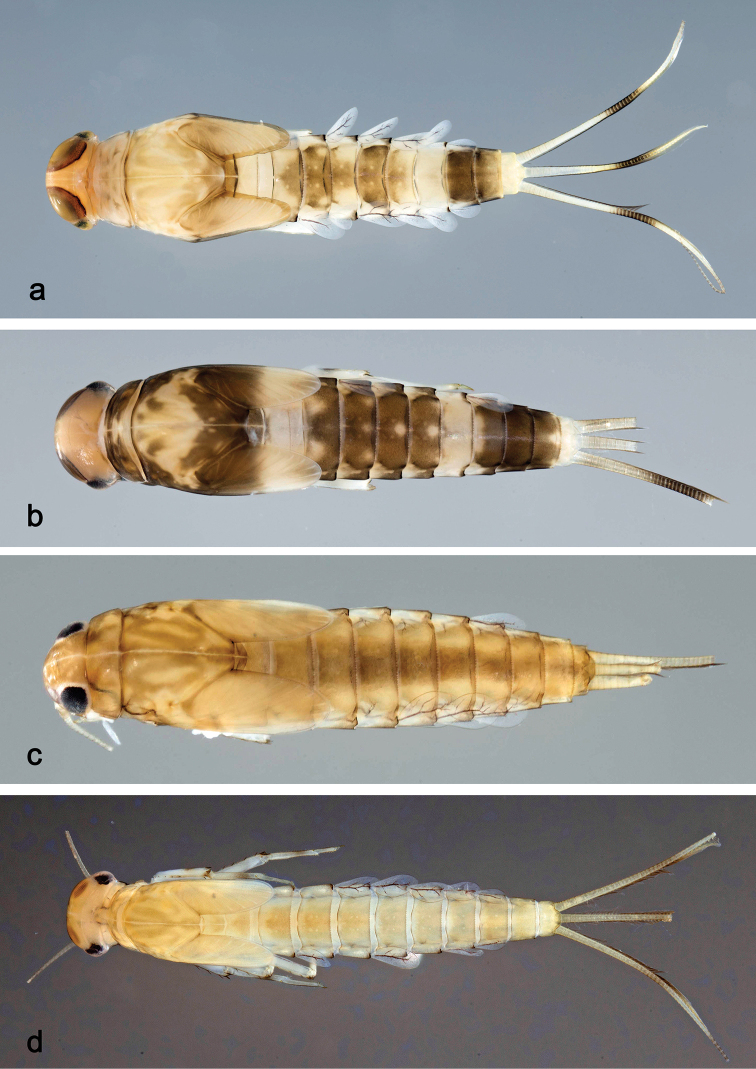
Habitus, larvae, dorsal view **a***Labiobaetis valdezorum* sp. nov. **b***Labiobaetis wantzeni* sp. nov. **c***Labiobaetis baganii* sp. nov. **d***Labiobaetis delocadoi* sp. nov.

### Species groups

The morphological groups within *Labiobaetis* are primarily a working tool but could also serve as a basis for future studies on the generic delimitation and phylogeny of this genus. The inclusion of nuclear gene sequences may prove that some are natural groups. Most of the species in the Philippines belong to three different groups only, six to the *numeratus* group, five to the *operosus* group, and six to the *sumigarensis* group. These groups are widespread and highly diversified in Asia. Species of the *numeratus* group are also known from Sri Lanka, Malaysia, Indonesia and China, species of the *operosus* group from Malaysia and Indonesia and species of the *sumigarensis* group from India, Sri Lanka, Malaysia, Indonesia, Brunei, China and Taiwan (Müller-Liebenau 1984; Müller-Liebenau and Hubbard 1985; Kang et al. 1994; Shi and Tong 2014; Kubendran et al. 2015; Kaltenbach and Gattolliat 2019, 2020). None of these groups are known from New Guinea (Kaltenbach and Gattolliat 2018).

**Figure 45.**
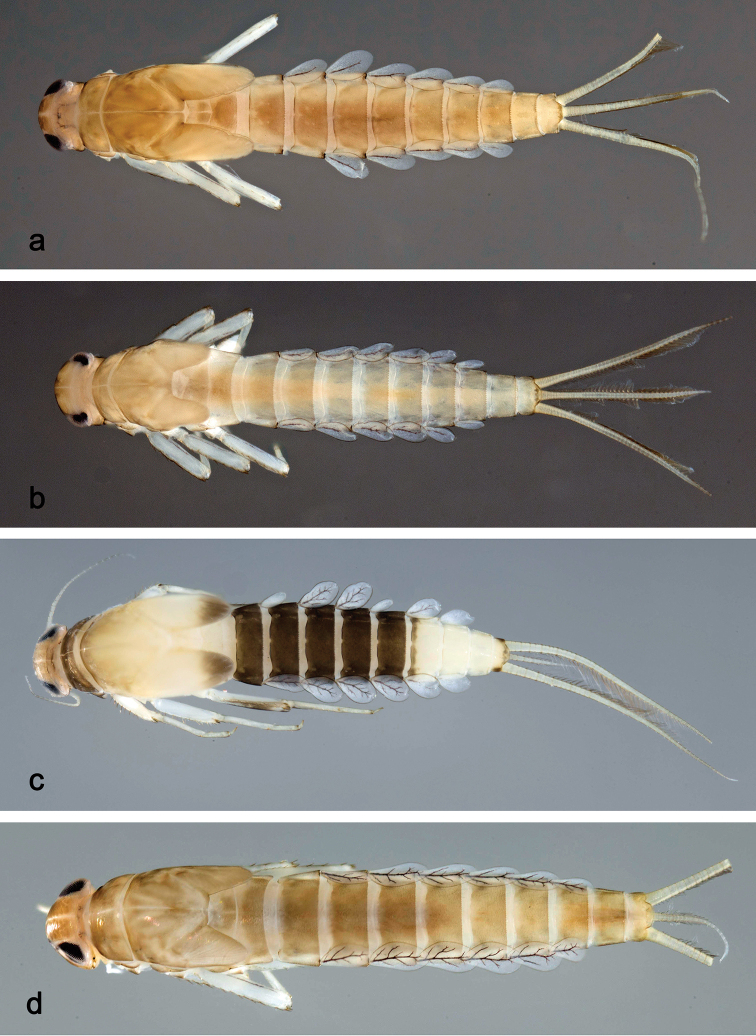
Habitus, larvae, dorsal view **a***Labiobaetis freitagi* sp. nov. **b***Labiobaetis pelingeni* sp. nov. **c***Labiobaetis giselae* sp. nov. **d***Labiobaetis mendozai* sp. nov.

Interestingly, a few of these newly described species share characters with the fauna of New Guinea. *Labiobaetis dalisay* sp. nov. shares the dendritic setae on the dorsal surface of the labrum with *L. dendrisetis* Kaltenbach & Gattoliat from New Guinea; it also has a similar labial palp and maxillary palp and seven pairs of gills (Kaltenbach and Gattolliat 2018). So far, *L. dendrisetis* is the only species with seven pairs of gills in New Guinea. Therefore, we erect the *dendrisetis* group of species including these two species. However, there are also important differences between the two species: *L. dalisay* sp. nov. has a well-developed scape process, hind protoptera and a femoral patch, while *L. dendrisetis* is missing these characters. Because of these important differences, especially the absence of a femoral patch in *L. dendrisetis*, we consider this group to be preliminary. Further species with dendritic setae either from the Philippines or from New Guinea may clarify the validity of this group in the future.

**Figure 46.**
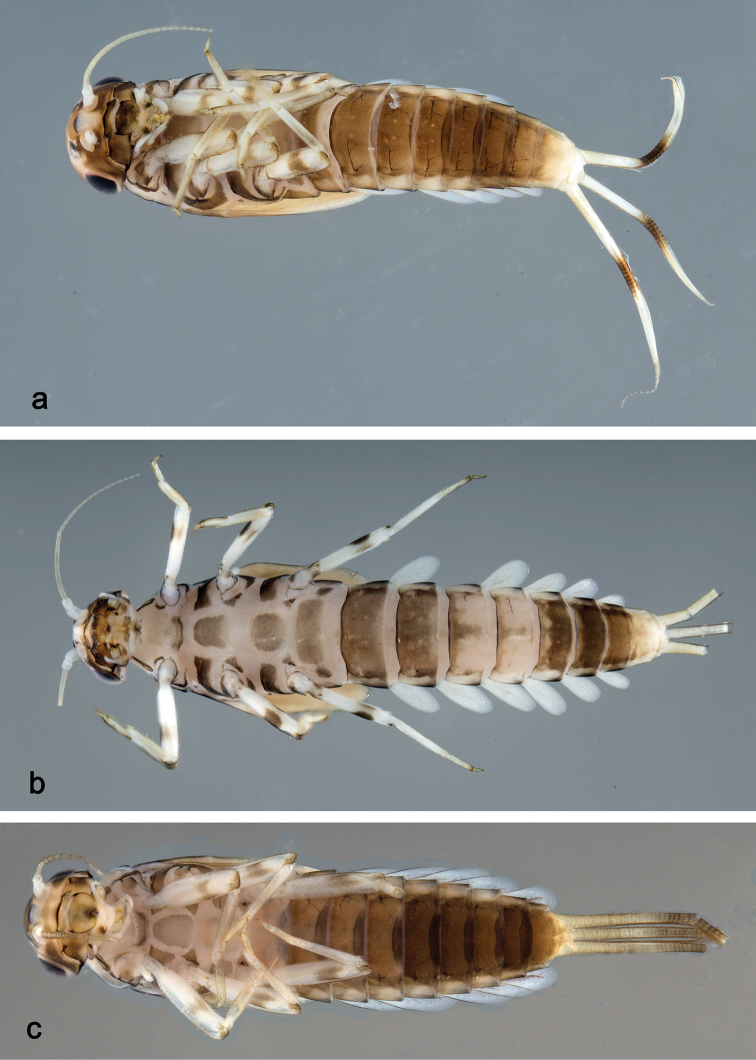
Habitus, larvae, ventral view **a***Labiobaetis acei* sp. nov. **b***Labiobaetis aldabae* sp. nov. **c***Labiobaetis camiguinensis* sp. nov.

*Labiobaetis giselae* sp. nov. and *L. mendozai* sp. nov. share the lanceolate setae on the dorsal surface of the labrum with *L. vallus* Kaltenbach & Gattolliat from New Guinea, have a similar labial palp and also no scape process, no hind protoptera and six pairs of gills (Kaltenbach and Gattolliat 2018). We therefore erect the *vallus* group of species to include these three species. The lanceolate, apically pointed setae on the dorsal surface of the labrum as well as the short paracercus (unknown in *L. vallus*) are unusual in *Labiobaetis*, but the protogonostyli developing under the larval cuticle of male late instar larvae of *L. mendozai* sp. nov. are folded as in the *Labiobaetis* type (Kluge 2004: fig. 29I) and all three species have a distolateral protuberance on segment II of the labial palp, which is an important character of *Labiobaetis*. Therefore, we have no doubt concerning the assignment of these species to *Labiobaetis*.

**Figure 47.**
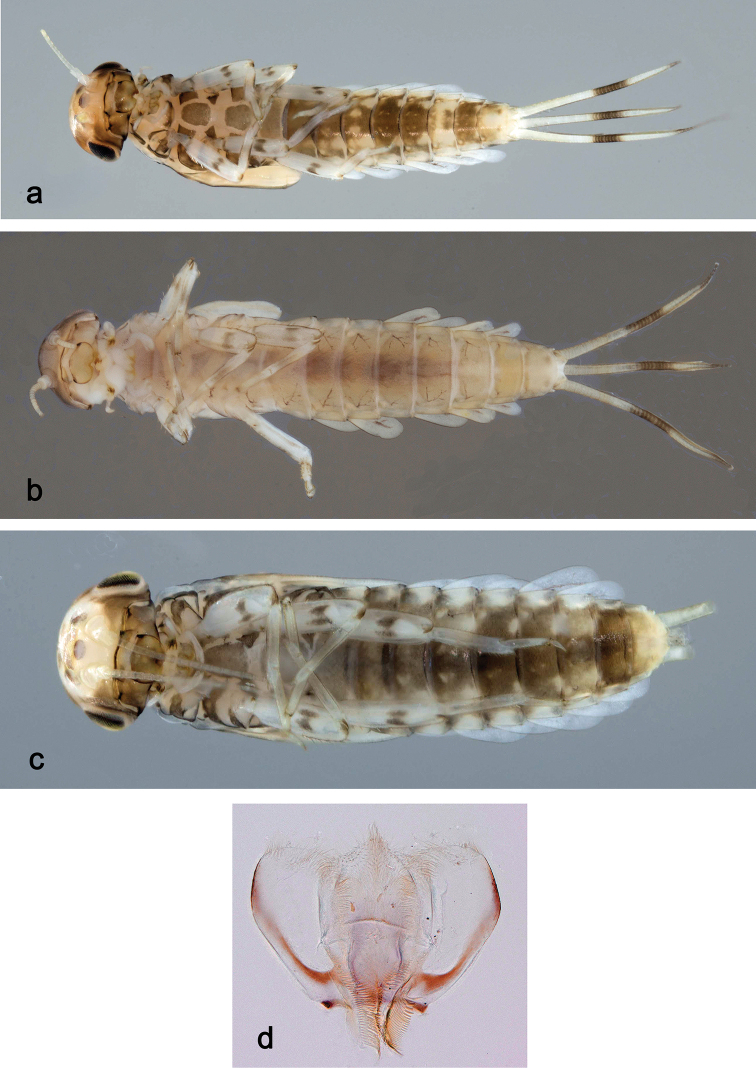
Habitus, larvae, ventral view **a***Labiobaetis lachicae* sp. nov. **b***Labiobaetis palawano* sp. nov. **c***Labiobaetis sabordoi* sp. nov. **d** Hypopharynx and superlinguae of *Labiobaetis camiguinensis* sp. nov.

### Genetic distance

**Figure 48.**
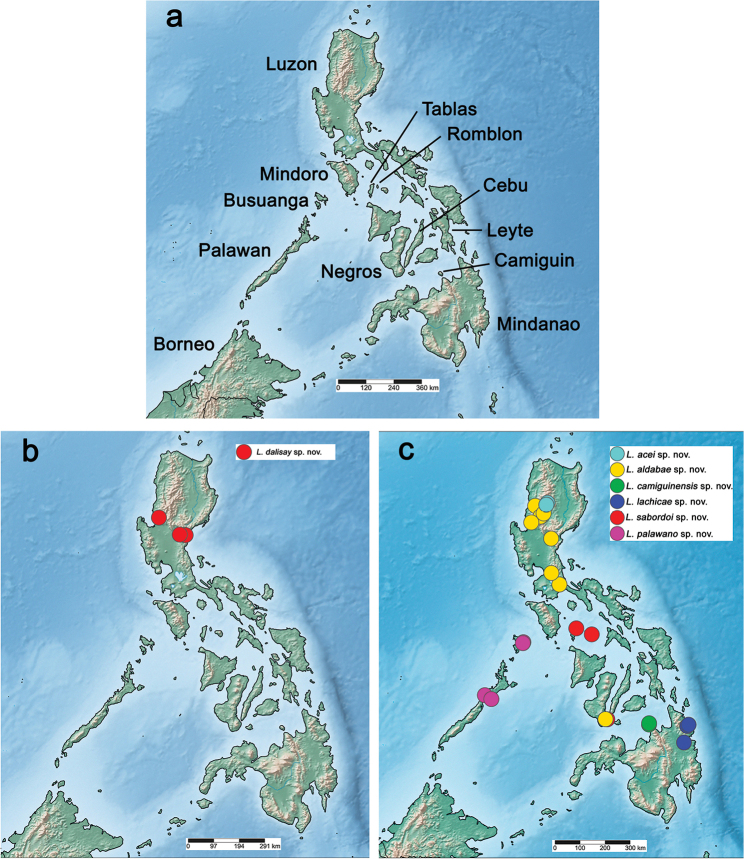
Distribution of *Labiobaetis* in the Philippines **a** Philippines, overview **b** Group *dendrisetis***c** Group *numeratus*.

In general, the genetic distances between the different species of *Labiobaetis* in the Philippines are rather high, between 15% and 27% (K2P, Table 3), which is in line with the genetic distances found in New Guinea (average 22%; Kaltenbach and Gattolliat 2018), Indonesia (11%–24%; Kaltenbach and Gattolliat 2019) and Borneo (19%–25%; Kaltenbach and Gattolliat 2020). Ball et al. (2005) reported a mean interspecific, congeneric distance of 18% for mayflies from the United States and Canada. The intraspecific distances are mostly very low as expected, ranging from 0 % to 3% (K2P). This result is certainly biased as it is based on a limited number of sequenced specimens per species, which were often from a single population. The exception is *L. aldabae* sp. nov. with up to 5% genetic distance between different locations on the same island (Luzon) and 6% between locations on Luzon and Negros (Fig. 48c). Here, the larger genetic distance may be explained by a possible isolation of some locations in northern mountainous areas of Luzon, while others may be connected between themselves and to other locations in the South, as well as by the geographic distance and probably stronger isolation between the islands of Luzon and Negros. Interestingly, the location with a genetic distance of 5% to other locations in Luzon has a genetic distance of only 2% to the sequenced specimen in Negros. Intraspecific distances of 4%–6% were also reported in some cases for *Labiobaetis* species in New Guinea, Indonesia and Borneo (Kaltenbach and Gattolliat 2018, 2019, 2020), as well as in aquatic beetles in the Philippines (Komarek and Freitag 2020). Ball et al. (2005) also reported a case with 6% intraspecific distance in a mayfly in North America and intraspecific K2P distances of more than 3.5% are not uncommon within Plecoptera as well (Gill et al. 2015; Gattolliat et al. 2016).

In addition to the species described in this paper, we obtained nine COI sequences with clearly interspecific genetic distance to other specimens with the same morphology. To give a more complete view on the diversity, including this cryptic diversity, we are presenting them as Molecular Operational Taxonomic Units (MOTUs) based on genetic evidence only (COI; Table 4). MOTUs were originally defined and used to investigate and cluster the enormous diversity of small organisms like nematodes or foraminifera (Floyd et al. 2002, Blaxter et al. 2005, Morard et al. 2016). All identified MOTUs of *Labiobaetis* belong to the dominant species groups of the Philippines (group *numeratus*, *operosus*, and *sumigarensis*). Because of the absence of morphological support, they remain as species hypotheses for now without further treatment in this paper. Additional material and investigations will be necessary to confirm their status in the future.

**Table 4.** Molecular Operational Taxonomic Units (MOTUs) of *Labiobaetis* in the Philippines.

MOTU denomination	Species group	Locality	Specimens catalog #	GenBank #	GenSeq
				(COI)	Nomenclature
L. cf. lachicae sp. nov. I	*numeratus* group	Philippines: Mindanao	GBIFCH 00654893	MT830932	genseq-4 COI
L. cf. aldabae sp. nov. I	*numeratus* group	Philippines: Luzon	GBIFCH 00654905	MT830934	genseq-4 COI
L. cf. wantzeni sp. nov. I	*operosus* group	Philippines: Mindanao	GBIFCH 00654876	MT830931	genseq-4 COI
L. cf. wantzeni sp. nov. II	*operosus* group	Philippines: Mindanao	GBIFCH 00763665	MT830937	genseq-4 COI
L. cf. wantzeni sp. nov. III	*operosus* group	Philippines: Mindanao	GBIFCH 00763666	MT830938	genseq-4 COI
L. cf. baganii sp. nov. I	*sumigarensis* group	Philippines: Camiguin	GBIFCH 00654887	MT830933	genseq-4 COI
L. cf. delocadoi sp. nov. I	*sumigarensis* group	Philippines: Negros	GBIFCH 00654890	MT830935	genseq-4 COI
L. cf. pelingeni sp. nov. I	*sumigarensis* group	Philippines: Luzon	GBIFCH 00763654	MT830936	genseq-4 COI
L. cf. molawinensis I	*sumigarensis* group	Philippines: Mindanao	GBIFCH 00763673	MT830939	genseq-4 COI

### Biogeography and endemicity

As mentioned above, of the 21 species of *Labiobaetis* known from the Philippines, six belong to the *numeratus* group, five to the *operosus* group and six to the *sumigarensis* group. All these groups are widespread and highly diversified in Southeast Asia. Members of *sumigarensis* and *operosus* group are also known from Borneo (Kaltenbach and Gattolliat 2020) and members of *numeratus* group from Sumatra and Sulawesi (Kaltenbach and Gattolliat 2019). From Taiwan only one species of the *sumigarensis* group and other species without relation to the Philippines are known (Kang et al. 1994; Kang and Yang 1996) and none of these groups are represented in New Guinea (Kaltenbach and Gattolliat 2018). This points to the direction of a single or very limited colonisation events for each of these groups in the past from Southeast Asia, probably facilitated by the at least partial land bridges between the Philippines and Borneo during Pleistocene (Brown and Diesmos 2010), and followed by local radiation in the Philippines. Additionally, there could have been a limited stepping-stone exchange between the Philippines and New Guinea, as we found members of the group *vallus* and *dendrisetis* on both these archipelagos (Kaltenbach and Gattolliat 2018) and both groups are not known from anywhere else.

Based on the present data, all the Philippine *Labiobaetis* species are endemic to the Philippines. Moreover, most species (14) are restricted to one island. Although partly due to localities missing in this study, island endemics are quite common among aquatic insect species of the Philippines (Freitag and Balke 2011; Freitag 2013; Freitag and Zettel 2013; Komarek and Freitag 2014, 2020; Vidal et al. 2017; Garces et al. 2018, 2020; Pelingen and Freitag 2020). This island endemic pattern suggests allopatry as a major driver of speciation, as already discussed for *Labiobaetis* in New Guinea and in Indonesia (Kaltenbach and Gattolliat 2018 and citations therein, Kaltenbach and Gattolliat 2019).

In a few cases, we have more than one species of the same species group occuring on the same island: *L. acei* sp. nov. and *L. aldabae* sp. nov. from *numeratus* group on Luzon; *L. sumigarensis* and *L. molawinensis* from *sumigarensis* group on Luzon. Moreover, we have additional diversity as MOTUs partly sharing the same species groups and islands (Table 4). Although the high diversity in Luzon can be considered as sampling bias given that most expeditions and course projects of the Ateneo laboratory were done in Luzon, it may also be due to the diversity of sampling habitats included. Among the material examined, samples from Luzon have the highest altitudes (up to 1,800 m). This indicates that there are certainly other mechanisms of speciation involved as well, such as differentiation along elevational and environmental gradients or rising and falling sea levels between mid to late Pleistocene with subsequent separation and re-connection of islands (Brown and Diesmos 2010). In a recent study of the structuration of the mayfly community on three neighbouring volcanos in Sumatra, elevation was found to be the only factor driving the within-species genetic structuring of two species of Baetidae and an important factor for two others (Gueuning et al. 2017). On the same volcanos, Kaltenbach et al. (2020) reported two different species of *Procerobaetis* Kaltenbach & Gattolliat, 2020, at different elevations and ecological conditions, which points to the direction that these factors could be drivers of speciation. In the caddisfly genus *Hydropsyche* Pictet, 1834 Mey (2003) reported adaptive radiation of the *hamifera* group from spring brooks in the highest mountains to the slowly flowing sections in the lowlands in Luzon that gave rise to the high species diversity on the island.

We also have cases of species, which are distributed on more than one island: *L. baganii* sp. nov. and *L. wantzeni* sp. nov. both on Mindanao and Camiguin and with a genetic distance of 2% between the islands; *L. sabordoi* sp. nov. on Negros and Romblon (K2P 1%); *L. aldabae* sp. nov. on Luzon and Negros (K2P 2%–6%); *L. gamay* sp. nov. on Luzon and Mindoro (K2P 0%–1%); *L. pelingeni* sp. nov. on Negros and Cebu (K2P 3%); *L. delocadoi* sp. nov. on Cebu and Leyte (K2P 2%). Based on the small genetic distances and the mostly close geographic distances, these cases suggest either current or remnant intra-archipelagic dispersal crossing sea channels. Interestingly, most of these island pairs do not belong to the same intra-Philippine biogeographic region (Greater Luzon, Greater Mindoro, Romblon, West Visayas, Camiguin, and Greater Mindanao) (Ong et al. 2002). Even the smaller islands (Camiguin, Romblon) included here were never connected by aggregate to islands complexes during Pleistocene. We can also expect that additional data may increase the number of species present on more than one island.

Four species in Palawan offer an interesting zoogeographic affinity based on morphology. The morphology of *L. freitagi* sp. nov. (*sumigarensis* group) is closer to *L. delocadoi* sp. nov. than to the species from Borneo (Kaltenbach and Gattolliat 2020). The morphology of *L. palawano* sp. nov. (*numeratus* group) presents more affinities with *L. sabordoi* sp. nov., *L. acei* sp. nov. and *L. aldabae* sp. nov. than with the species from Indonesia (Kaltenbach and Gattolliat 2019). The morphologies of *L. tagbanwa* sp. nov. and *L. pangantihoni* sp. nov. (*operosus* group) do not show more similarities with other species from the Philippines than to *L. dayakorum* Kaltenbach & Gattolliat from Borneo and *L. paraoperosus* Kaltenbach & Gattolliat from Sumatra (Kaltenbach and Gattolliat 2019, 2020). This suggests that at least *L. freitagi* sp. nov. and *L. palawano* sp. nov. are part of the local radiation in the Philippines and are not remaining species from a possible colonisation across the Palawan land bridge during Pleistocene.

**Figure 49.**
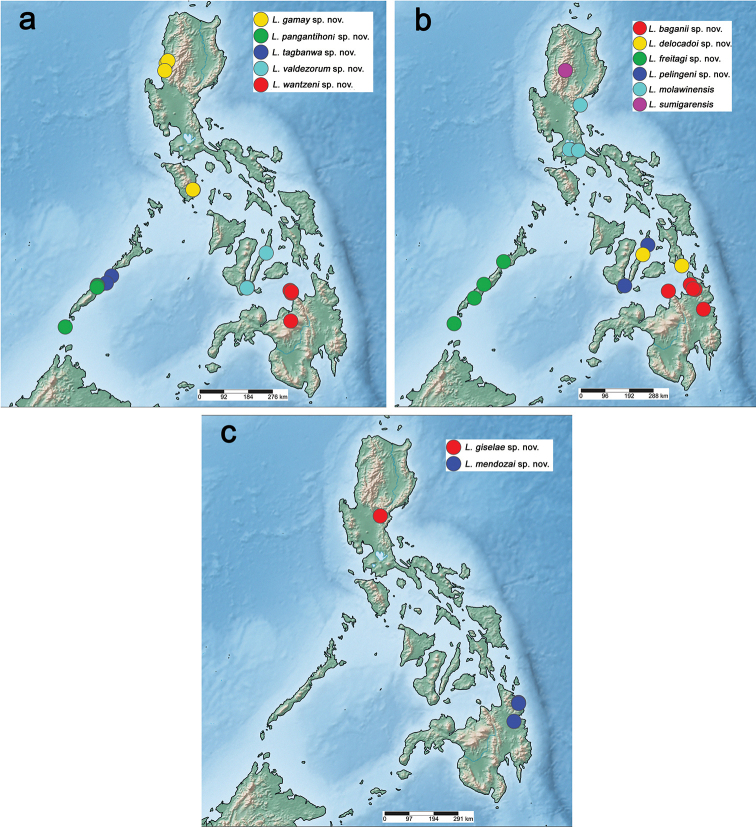
Distribution of *Labiobaetis* in the Philippines **a** Group *operosus***b** Group *sumigarensis***c** Group *vallus*.

The number of sampled localities and different habitats is still limited and there are regions without any collection activities so far (Figs 48, 49). High-altitude gradients can be found on several islands in the Philippines, incl. the under-sampled Mindanao and Palawan. In addition, we have nine species hypotheses based on genetics only (MOTUs, Table 4), which may be confirmed as valid species in the future. Therefore, we can expect that the number of *Labiobaetis* species in the Philippines will continue to increase substantially with further collections.

## Supplementary Material

XML Treatment for Labiobaetis dalisay

XML Treatment for Labiobaetis acei

XML Treatment for Labiobaetis aldabae

XML Treatment for Labiobaetis camiguinensis

XML Treatment for Labiobaetis lachicae

XML Treatment for Labiobaetis palawano

XML Treatment for Labiobaetis sabordoi

XML Treatment for Labiobaetis gamay

XML Treatment for Labiobaetis pangantihoni

XML Treatment for Labiobaetis tagbanwa

XML Treatment for Labiobaetis valdezorum

XML Treatment for Labiobaetis wantzeni

XML Treatment for Labiobaetis molawinensis

XML Treatment for Labiobaetis sumigarensis

XML Treatment for Labiobaetis baganii

XML Treatment for Labiobaetis delocadoi

XML Treatment for Labiobaetis freitagi

XML Treatment for Labiobaetis pelingeni

XML Treatment for Labiobaetis giselae

XML Treatment for Labiobaetis mendozai

## Footnotes

Some of the species may be difficult to identify below the species group level, more than one good slide is recommended
